# PTEN deficiency confers sensitivity to ATR inhibitor–based treatment in high-grade serous ovarian cancer

**DOI:** 10.1172/JCI197645

**Published:** 2026-07-07

**Authors:** Jie Hao, Bose Kochupurakkal, Timothy B. Branigan, Ozge Sezin Somuncu, Renyan Liu, Heta Jadhav, Alexandre André B. A. da Costa, Yuqing Jiao, Jenny Z. Yu, David B. Martignetti, Golbahar Sadatrezaei, Sirisha Mukkavalli, Prafulla C. Gokhale, Su-Chun Cheng, Steven J. Skates, Dimitrios Nasioudis, Panagiotis A. Konstantinopoulos, Joyce F. Liu, Stephanie Gaillard, Robert L. Giuntoli, Lainie Martin, Janos Tanyi, Nawar Latif, Ian Heller, Fiona Simpkins, Kalindi Parmar, Alan D. D’Andrea, Geoffrey I. Shapiro

**Affiliations:** 1 Department of Medical Oncology, Dana-Farber Cancer Institute (DFCI), Boston, Massachusetts, USA.; 2 Department of Medicine, Brigham and Women’s Hospital and Harvard Medical School, Boston, Massachusetts, USA.; 3 Center for DNA Damage and Repair, DFCI, Boston, Massachusetts, USA.; 4 Department of Radiation Oncology, DFCI and Harvard Medical School, Boston, Massachusetts, USA.; 5 Belfer Center for Applied Cancer Science, DFCI, Boston, Massachusetts, USA.; 6 Department of Data Science, DFCI and Harvard H.T. Chan School of Public Health, Boston, Massachusetts, USA.; 7 Massachusetts General Hospital Biostatistics and Harvard Medical School, Boston, Massachusetts, USA.; 8 Ovarian Cancer Research Center, Perelman School of Medicine, University of Pennsylvania, Philadelphia, Pennsylvania, USA.; 9 Departments of Oncology and Gynecology and Obstetrics, Johns Hopkins University School of Medicine, Baltimore, Maryland, USA.

**Keywords:** Cell biology, Clinical Research, Oncology, Cancer, DNA repair, Drug therapy

## Abstract

Ataxia telangiectasia and Rad3-related (ATR) inhibition is under evaluation for the treatment of high-grade serous ovarian cancer (HGSOC) to reverse acquired resistance to poly (ADP-ribose) polymerase (PARP) inhibition and to exacerbate chemotherapy-induced replicative stress. Here, we define PTEN deficiency as a predictive biomarker for the response to ATR inhibition, as monotherapy and in combination with PARP inhibition or gemcitabine. In response to ATR inhibition and compared with PTEN-proficient cells, PTEN-deficient cells are prone to (a) uncoupling of DNA polymerase and helicase activities, leading to excessive ssDNA and replication stress; (b) cytoplasmic sequestration of checkpoint kinase 1 (CHK1), compromising cell-cycle checkpoint control with reduced compensatory effects by ataxia-telangiectasia mutated (ATM) and DNA–dependent proteinase K (DNA-PK), leading to mitotic catastrophe; and (c) reduced DNA repair protein RAD51 homolog 1 (RAD51) recruitment, exacerbating replication fork instability, also leading to lethality. Retrospective analyses revealed that patients with HGSOC who expressed low PTEN levels experienced greater clinical benefit on ATR inhibitor–based trials than did those with high PTEN levels. These results justify prospective trials evaluating ATR inhibition as a therapeutic strategy for PTEN-deficient tumors.

## Abstract 2

Hao et al. have demonstrated that PTEN deficiency confers sensitivity to ATR inhibitor-based treatment in preclinical models and clinical trials of high-grade serous ovarian cancer.

## Graphical abstract

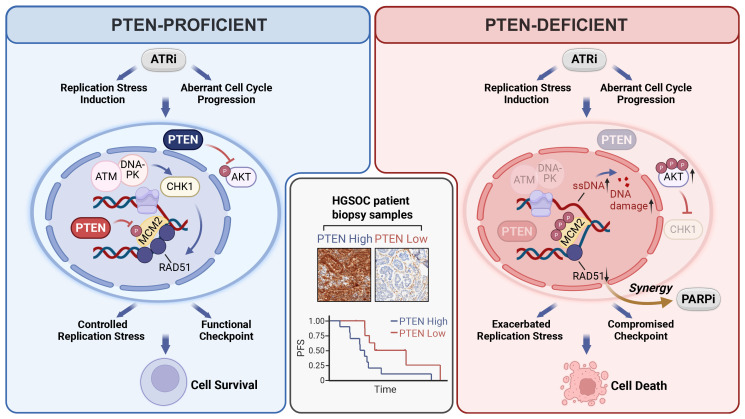

## Introduction

High-grade serous ovarian cancer (HGSOC), characterized by high mortality rates, poses significant treatment challenges due to late diagnosis and aggressive biology (1). More than 50% of these cancers harbor homologous recombination (HR) repair deficiency (2). Among this subset, inhibitors of poly (ADP-ribose) polymerase (PARP) have provided substantial clinical benefit, particularly for patients with breast cancer gene 1/-2–deficient (BRCA1/2-deficient) cancers, who are treated in the maintenance setting following a response to platinum-based chemotherapy (3, 4). PARP inhibitors exert synthetic lethality in HR repair–deficient tumors by a variety of mechanisms, including inhibition of base excision repair, PARP trapping, and induction of ssDNA gaps (5, 6). Nonetheless, intrinsic and acquired PARP inhibitor resistance remains a significant unmet medical need, also mediated by heterogeneous mechanisms, so that the development of strategies to reverse PARP inhibitor resistance is therefore of high priority.

In HGSOC, inhibitors of the ataxia telangiectasia and RAD3-related–checkpoint kianse 1–Wee1-like protein kinase (ATR-CHK1-WEE1) axis have produced clinical responses either as monotherapy or when combined with PARP inhibition in PARP inhibitor–resistant tumors (7–11). In preclinical models and in clinical samples, these agents have been shown to reverse restored HR repair, destabilize replication forks, and induce ssDNA gap formation, thereby resensitizing cells to PARP inhibition (12–16).

In addition, the ATR pathway is an essential component of the cellular response to replication stress, ensuring integrity of replication forks and preventing cells from entering mitosis with incompletely replicated genomes (17, 18). Consequently, the addition of ATR pathway inhibitors to chemotherapy agents such as gemcitabine has exacerbated replication stress and promoted premature mitotic entry accompanied by increased DNA damage, translating to improved clinical outcomes (19–21).

Despite the promise of these approaches, ATR inhibition is routinely complicated by significant anemia (22–24). ATR pathway inhibitor combinations have also been limited by overlapping hematologic toxicities, frequently requiring attenuation of full doses or schedules of both agents (8, 9, 24). Therefore, identifying predictive biomarkers for the ATR inhibitor response, either as monotherapy or in combination, could optimize patient selection strategies and minimize potential toxicities. Although ataxia telangiectasia mutated (ATM) deficiency has long been considered as a possible biomarker for ATR inhibition based on expected synthetic lethality (24, 25), confirmed response rates for ATR inhibitors in ATM-deficient tumors have not been robust (26–28).

CRISPR screens performed in both WT and *Atm*-KO mouse embryonic stem cells suggest that loss of the *PTEN* tumor suppressor may also confer sensitivity to ATR inhibition (29). However, the mechanistic basis for a synthetic lethal interaction between PTEN deficiency and ATR inhibition remains unclear. PTEN exhibits both protein and lipid phosphatase activities (30, 31), but the extent to which loss of these functions sensitizes to ATR inhibition is yet to be elucidated. Resolving these mechanistic questions will enable more effective use of *PTEN* loss as a potential biomarker for ATR inhibitor–based therapies.

Here, we interrogated PTEN deficiency in concert with ATR inhibition used alone or in combination with PARP inhibition or gemcitabine in HGSOC models. Compared with PTEN-proficient cells, in response to ATR inhibition, cells with PTEN deficiency showed increased replication stress related to uncoupling of DNA polymerase and helicase activities, compromised cell-cycle checkpoint function, and reduced DNA repair protein RAD51 homolog 1-mediated (RAD51-mediated) replication fork stabilization. Furthermore, retrospective analyses indicated more favorable outcomes with ATR inhibitor–based treatment among patients with HGSOC harboring PTEN deficiency, suggesting a promising biomarker as these strategies are further developed in treatment-resistant populations.

## Results

### Deficiency of PTEN protein and lipid phosphatase activities confers ATR inhibitor sensitivity.

**Figure 1:**
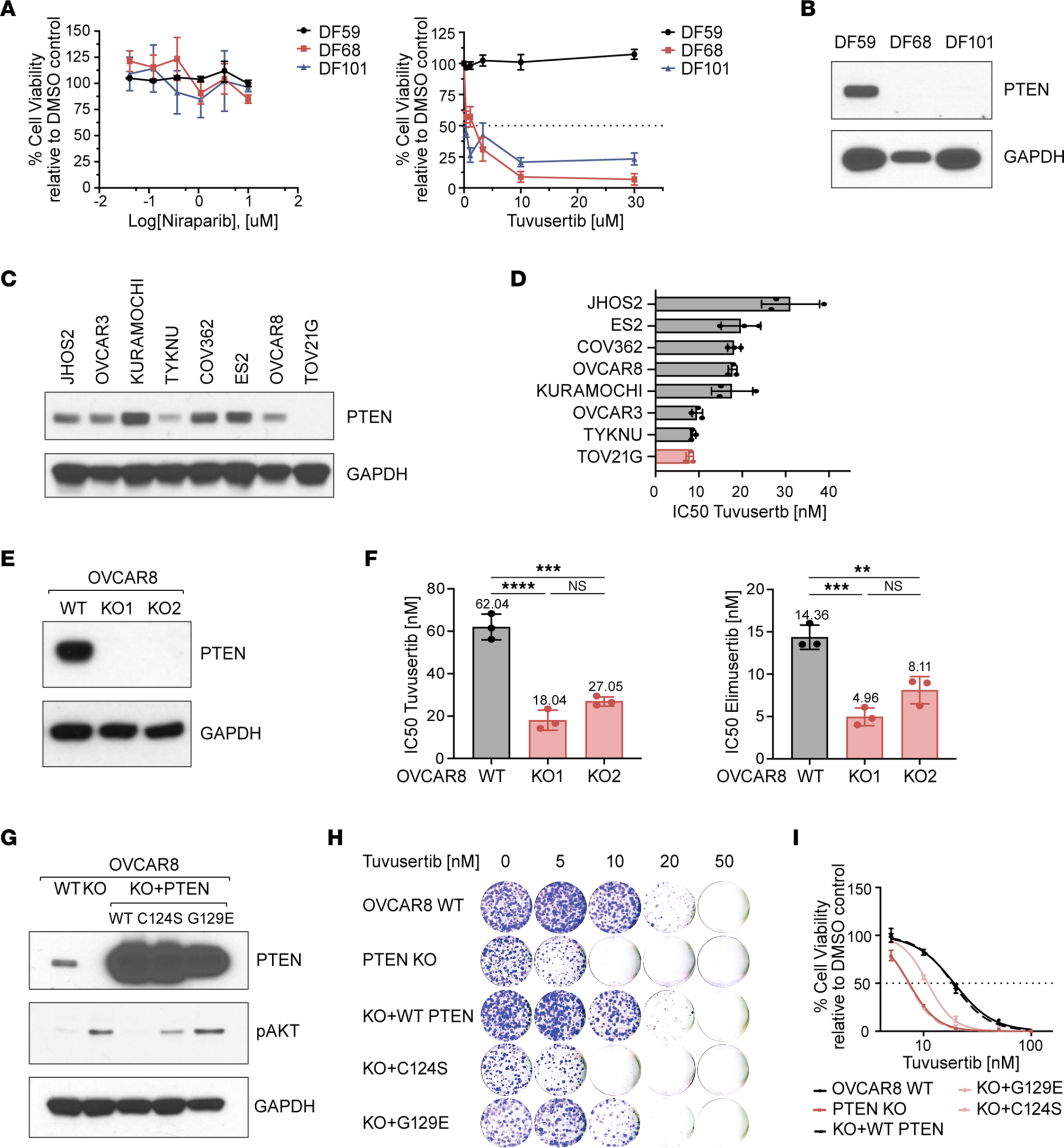
Deficiency of PTEN protein and lipid phosphatase activities confers ATR inhibitor sensitivity. (**A**) PARP inhibitor–resistant PDXOs of ovarian cancer exhibit variable sensitivity to the ATR inhibitor tuvusertib. Data represent the mean ± SD. (**B**) Western blot analysis of PTEN expression in PDXO models showing PTEN deficiency in tuvusertib-sensitive models. (**C**) Western blot analysis of PTEN expression in ovarian cancer cell lines. (**D**) Colony formation assay evaluating tuvusertib sensitivity across ovarian cancer cell lines. PTEN-deficient TOV21G cells show the highest sensitivity. Data represent the mean ± SD. (**E**) Western blot analysis of lysates from WT and PTEN CRISPR-KO cells, confirming PTEN loss in KO clones. (**F**) CellTiter-Glo (CTG) assay showing increased sensitivity of PTEN-KO clones to ATR inhibition. Data represent the mean ± SD. **P < 0.01, ***P < 0.001, and ****P < 0.0001, by 1-way ANOVA with Tukey’s post hoc test. (**G**) Reexpression of WT and mutant PTEN in PTEN-KO cells via lentiviral transduction. (**H**) Colony formation assay demonstrating enhanced sensitivity of PTEN-KO cells to tuvusertib and the rescue effect of PTEN reexpression. (**I**) Quantification of colony formation assays using ImageJ. Data represent the mean ± SD.

To evaluate the effects of ATR inhibition in PARP inhibitor-resistant HGSOC models, we treated organoid cultures generated from 3 previously characterized *BRCA1-*mutant patient-derived xenograft (PDX) models (DF59, DF68, and DF101). These PDX-derived organoids (PDXOs) were resistant to PARP inhibition (Figure 1A, left), consistent with the known restoration of HR in these models (14, 32). However, these PDXOs had variable sensitivity to ATR inhibition. Compared with DF59, the PDXOs DF68 and DF101 exhibited markedly higher sensitivity to the ATR inhibitor tuvusertib (M1774) (Figure 1A, right). DF68 and DF101, but not DF59, were reported to have *PTEN* copy loss based on whole-exome sequencing of the parental PDX models (14). Consistent with these sequencing data, we did not detect PTEN protein expression in the DF68 or DF101 PDXOs by Western blotting (Figure 1B), raising the possibility that PTEN loss increased sensitivity to ATR inhibitor monotherapy.

To further examine the association of PTEN deficiency with ATR inhibitor sensitivity, we tested PTEN protein expression and tuvusertib sensitivity in 10 additional ovarian cancer cell lines (Figure 1, C and D, and Supplemental Figure 1, A–D; supplemental material available online with this article; https://doi.org/10.1172/JCI197645DS1). TOV21G, an ovarian cancer cell line with known *PTEN* deletion (33, 34), had no PTEN expression (Figure 1C) and was the most sensitive cell line (Figure 1D). Additionally, we examined PTEN expression and ATR inhibitor sensitivity in the *BRCA2*-mutant PEO1 and PEO4 cell line pair, which originated from the same patient; however, PEO4 carries a *BRCA2* reversion mutation conferring PARP inhibitor resistance (35, 36). Notably, PEO4 cells exhibited higher PTEN expression compared with PEO1 cells. Cell viability assays demonstrated that PTEN^lo^-expressing PEO1 cells were approximately 5 times more sensitive to tuvusertib than PTEN^hi^- expressing PEO4 cells, as confirmed by colony formation assays (Supplemental Figure 1, A–D). Furthermore, HGSOC cell lines in the DepMap datasets harboring *PTEN* mutations were also more sensitive to the ATR inhibitors ceralasertib (AZD6738), VE-821, and AZ20, as reflected by more negative log_2_ fold-change values compared with control treatment (Supplemental Figure 1E). Together, these data suggest that ATR inhibitor sensitivity increased with PTEN loss.

To directly assess the effect of PTEN loss on ATR inhibitor sensitivity, we generated PTEN-KO cells from the OVCAR8 cell line using CRISPR/Cas9 technology. Western blots confirmed the absence of PTEN expression in KO clones (Figure 1E). Use of the isogenic pair allowed us to control for confounding baseline genomic alterations that drive replication stress, including *TP53* mutation, *BRCA1* methylation, *CCNE1* gain, and *MYC* amplification (33, 37–39). Drug sensitivity assays revealed that PTEN-KO cells had significantly lower IC_50_ values for ATR inhibitors than did PTEN-proficient parental cells (Figure 1F). We further confirmed the effects of PTEN KO in *BRCA1/2* WT TYKNU cells, in which PTEN deficiency also increased sensitivity to ATR inhibition (Supplemental Figure 1F). Additionally, PTEN KO in PEO4 conferred tuvusertib sensitivity (Supplemental Figure 1G), similar to that observed in PEO1 cells.

PTEN has both protein phosphatase and lipid phosphatase activities. To determine which of these activities influenced ATR inhibitor sensitivity, we reexpressed WT PTEN and 2 PTEN mutants (C124S and G129E) in PTEN-KO cells using lentiviral vectors. Both protein and lipid phosphatase activities are impaired in the C124S mutant, whereas the G129E mutant is only deficient in lipid phosphatase activity (30). Cells expressing these PTEN mutants were analyzed for tuvusertib sensitivity alongside cells with reexpressed WT PTEN. Western blots confirmed the expression of WT or mutant PTEN and the expected effects on phosphorylated AKT (pAKT) expression (Figure 1G). Colony formation assays indicated that WT PTEN fully restored resistance to tuvusertib, whereas the C124S mutant did not (Figure 1, H and I). The G129E mutant partially restored resistance, suggesting that loss of both protein and lipid phosphatase functions of PTEN contributed to ATR inhibitor sensitivity.

### Loss of PTEN leads to uncoupling of helicase and polymerase activities at the replication fork after ATR inhibition, resulting in the accumulation of ssDNA and replication stress.

ATR inhibitors induce replication fork dysregulation and compromise cell-cycle checkpoints to promote the accumulation of ssDNA and replication stress that triggers cell death (17, 18). ATR, as well as ATM, will slow replication fork progression under replication stress by phosphorylating S108 on the MCM2 subunit of the replicative MCM helicase (40), effectively acting as replication fork brakes. Upon ATR inhibition, the DNA polymerase stalls as demonstrated by nucleotide incorporation in fiber assays, leading to ssDNA accumulation unless the helicase is simultaneously slowed through alternative pathways. Similar to ATR, PTEN, as a protein phosphatase, can deactivate MCM2 by dephosphorylating the activating S41 site (41), raising the possibility that PTEN-deficient cells will be unable to restrict MCM2 during ATR inhibitor exposure, leading to greater accumulation of ssDNA and exacerbated replication stress.

**Figure 2:**
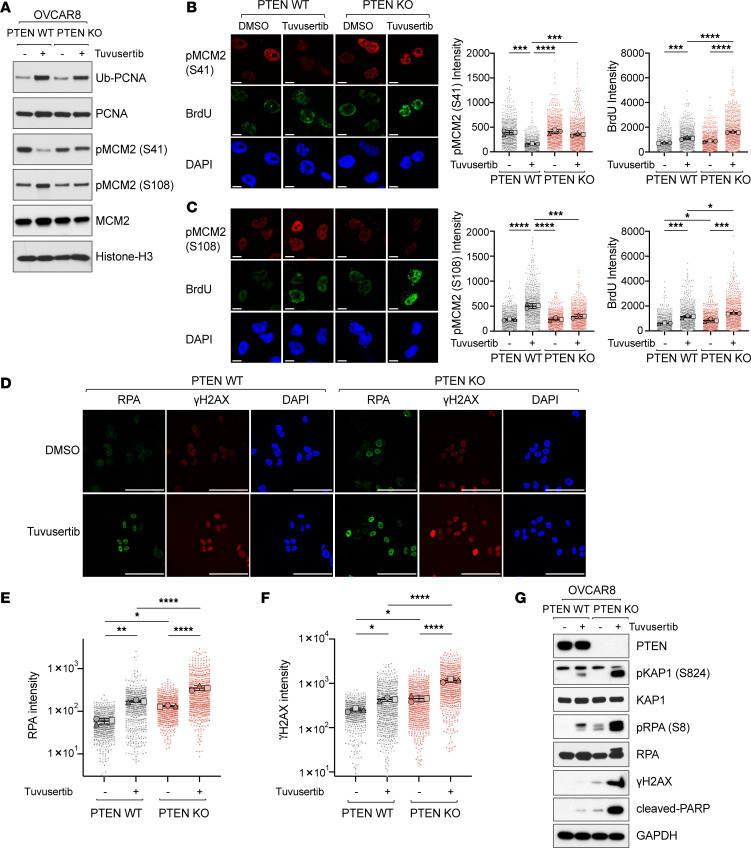
Loss of PTEN leads to uncoupling of helicase and polymerase activities at the replication fork after ATR inhibition, resulting in the accumulation of ssDNA and replication stress. (**A**) Western blot analysis of chromatin-bound fractions from WT and PTEN-KO cells after ATR inhibitor treatment, showing the expression of pMCM2 and Ub-PCNA. (**B** and **C**) Cells were labeled with BrdU (10 μM) for 48 hours, followed by treatment with DMSO or tuvusertib (1 μM) for 16 hours. Cells were then pre-extracted and subjected to native BrdU immunostaining to detect ssDNA. Samples were costained separately with antibodies against either pMCM2 S41 or pMCM2 S108. Original magnification, ×63. Scale bars: 10 μm. Fluorescence intensities were quantified using CellProfiler. (**D**) Immunofluorescence staining of RPA and γH2AX in OVCAR8 PTEN WT and PTEN-KO cells treated with DMSO or tuvusertib (1 μM) for 24 hours, following pre-extraction. Increased RS and DNA damage were observed in PTEN-deficient cells upon ATR inhibition. Scale bars: 100 μm. (**E** and **F**) Quantification of RPA and γH2AX fluorescence intensities using CellProfiler. Each dot represents an individual cell from 3 independent experiments, with at least 200 cells analyzed per experiment. Symbols represent the mean of each biological replicate. (**G**) Western blot analysis of lysates from OVCAR8 and OVCAR8-PTEN-KO cells treated with tuvusertib (1 μM) for 24 hours. ATR inhibition induces increased replication stress and DNA damage in PTEN-deficient cells. Data are presented as the mean ± SD (*n* = 3 independent experiments per group). Statistical significance was assessed using biological replicate means by 1-way ANOVA with Tukey’s multiple-comparison test (*P < 0.05, **P < 0.01, ***P < 0.001, and ****P < 0.0001).

To determine whether MCM2 regulation was altered in PTEN-deficient cells, we examined MCM2 pS41 and pS108 levels after ATR inhibition by Western blotting (Figure 2A). In both PTEN WT and PTEN-KO cells, we noted induction of ubiquitinated proliferating cell nuclear antigen (Ub-PCNA), a marker of stalled replication forks. However, pMCM2 S41 levels were reduced after ATR inhibition in PTEN WT, but not PTEN-KO, cells. In parallel, MCM2 pS108 levels were upregulated only in WT cells but not in KO cells, indicating a lack of MCM2 restriction after ATR inhibition in PTEN-deficient cells.

To define the catalytic requirements underlying PTEN-mediated regulation of MCM2 phosphorylation, we reconstituted PTEN-deficient cells with WT PTEN or separation-of-function mutants. Reexpression of WT PTEN fully restored physiological MCM2 phosphorylation dynamics in response to ATR inhibition (Supplemental Figure 2A). As expected, the catalytically inactive C124S mutant failed to restore suppression of activating pMCM2 S41 phosphorylation or induction of inhibitory pMCM2 S108 phosphorylation. In contrast, the G129E mutant, which has protein phosphatase but not lipid phosphatase activity, restored the reduction in pMCM2 S41 phosphorylation but failed to promote phosphorylation at the inhibitory S108 site. These findings reveal a dual regulatory mechanism whereby the protein phosphatase activity of PTEN was sufficient to restrain S41 phosphorylation, whereas its lipid phosphatase activity was required to facilitate S108 phosphorylation.

The hyperactivation of MCM2 after ATR inhibition in PTEN-deficient cells was accompanied by a reduction in fork speed (Supplemental Figure 2B). Although the reduction in fork speed was less pronounced in PTEN-KO cells compared with WT cells, persistent MCM2 activation after ATR inhibition in PTEN-KO cells resulted in uncoupling of helicase and polymerase activities with unrestrained DNA unwinding, whereas DNA polymerase activity was impeded.

To validate these findings at the single-cell level and to determine whether impaired MCM2 regulation was associated with ssDNA accumulation, we performed immunofluorescence costaining for pMCM2 S41 or pMCM2 S108 together with native BrdU (Figure 2, B and C). In PTEN-proficient cells, ATR inhibition reduced pMCM2 S41 and increased pMCM2 S108 staining, consistent with our Western blot results (Figure 2A) and with adaptive modulation of helicase activity under replication stress conditions. In contrast, PTEN-deficient cells failed to appropriately downregulate pMCM2 S41 or induce pMCM2 S108 following ATR inhibition. Notably, native BrdU staining was significantly increased in PTEN-deficient cells after ATR inhibition compared with PTEN-proficient cells, indicating enhanced accumulation of ssDNA, supporting the contention that PTEN loss impairs adaptive MCM2 phosphorylation dynamics and is associated with exacerbated replication stress following ATR inhibition.

To determine whether this specific dysregulation was sufficient to drive ssDNA accumulation, we expressed MCM2 phosphomutants in PTEN-proficient cells (Supplemental Figure 2C). Following ATR inhibition, cells expressing either the S41D phosphomimetic mutant or the S108A nonphosphorylatable mutant exhibited significantly increased native BrdU intensity compared with WT MCM2-expressing controls (Supplemental Figure 2D). Strikingly, the S41D/S108A double mutant produced markedly augmented accumulation of ssDNA, effectively phenocopying the elevated BrdU levels observed in PTEN-deficient cells. These data demonstrate that coordinated regulation of MCM2 phosphorylation at S41 and S108 was required to restrict helicase activity and prevent excessive DNA unwinding, and that disruption of this balance caused by PTEN deficiency was sufficient to drive excessive ssDNA accumulation under ATR inhibition.

In addition to native BrdU staining (Figure 2, B and C), we also quantified ssDNA levels using chromatin-bound replication protein A (RPA), a ssDNA-binding protein in PTEN WT and -KO cells treated with tuvusertib. As with native BrdU, the basal level of RPA on chromatin was higher in PTEN-deficient cells than in proficient cells and was further increased by ATR inhibition (Figure 2, D and E). Taken together, these results show that, in response to ATR inhibition, PTEN loss led to induction of pathological replication structures characterized by the accumulation of ssDNA resulting from MCM2 dysregulation.

Because these ssDNA structures resulting from impaired replication induced by ATR inhibition are converted into double-stranded breaks, we also assessed DNA damage and replication stress markers in response to tuvusertib treatment. Higher levels of phosphorylated histone H2AX (γH2AX) were induced by ATR inhibition in PTEN-KO cells compared with PTEN WT cells, as shown by immunofluorescence and Western blotting (Figure 2, D, F, and G). In concentration-dependent and time-course experiments, expression of γH2AX, as well as the replication stress markers phosphorylated Krüppel-associated box (KRAB)-associated protein 1 (pKAP1) and pRPA, was increased at lower concentrations of tuvusertib and earlier after treatment in PTEN-KO cells (Supplemental Figure 2, E and F). Basal levels of pRPA and cleaved PARP in addition to γH2AX were higher in PTEN-KO cells and were further increased after ATR inhibition (Figure 2G), suggesting that a higher degree of replication stress and cell death was induced by ATR inhibition in PTEN-KO cells compared with WT cells. Additionally, PTEN-deficient TOV21G cells exhibited a higher degree of replication stress (pRPA and pKAP1) and DNA damage (γH2AX) after tuvusertib treatment compared with the PTEN-proficient cell lines (Supplemental Figure 2G). Consequently, loss of PTEN predisposed cells to a higher degree of replication stress and DNA damage in response to ATR inhibition, consistent with dysregulation of MCM2 and replication fork dynamics.

### PTEN loss alters CHK1 nuclear localization, compromises cell-cycle checkpoint control, and reduces ATM and DNA-PK–mediated compensation after ATR inhibition.

**Figure 3:**
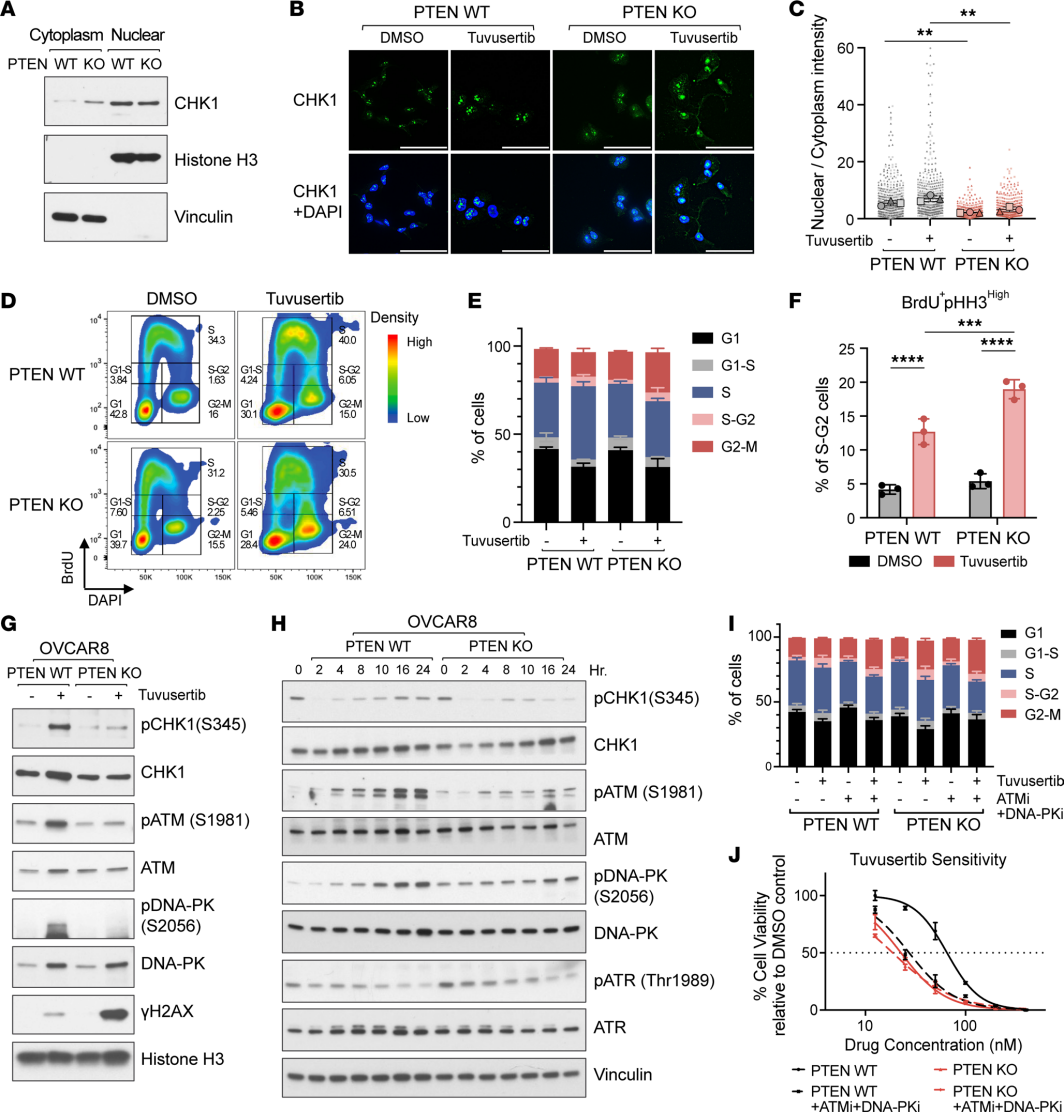
PTEN loss alters CHK1 nuclear localization, compromises cell-cycle checkpoint control, and reduces ATM and DNA-PK–mediated compensation after ATR inhibition. (**A**) Western blot analysis of nuclear and cytoplasmic fractions from WT and PTEN-KO cells showing CHK1 protein localization and distribution. (**B**) Immunofluorescence staining for CHK1 in PTEN WT and PTEN-KO cells to confirm subcellular localization changes. Scale bars: 100 μm. (**C**) Quantification of CHK1 nuclear-to-cytoplasmic fluorescence intensity ratios using CellProfiler. Each dot represents an individual cell from 3 independent experiments, with at least 200 cells analyzed per experiment. Symbols represent the mean of each biological replicate. Data are presented as the mean ± SD (*n* = 3 independent experiments per group). **P < 0.01; statistical significance was assessed using biological replicate means by 1-way ANOVA with Tukey’s multiple-comparison test. (**D**) PTEN-proficient and -deficient cells were treated with tuvusertib (1 μM, 24 h) and pulsed with BrdU (10 μM, 30 min). Cells were stained for pHH3, BrdU, and DAPI. Flow cytometric results for BrdU and DAPI are shown. (**E**) Cell-cycle distribution of PTEN WT and PTEN-KO cells, categorized by G_1_, G_1_/S, S, S/G_2_, and G_2_/M phases. (**F**) Quantification of BrdU^+^ and pHH3^hi^ cells, representing populations undergoing potential mitotic catastrophe. Data represent the mean ± SD. ***P < 0.001 and ****P < 0.0001, by 1-way ANOVA with Tukey’s post hoc test. (**G**) Western blot analysis of chromatin-bound fractions from cells treated with DMSO or tuvusertib (1 μM, 24 h), confirming reduced ATM and DNA-PK–mediated adaptation in PTEN-deficient cells upon ATR inhibition. (**H**) Western blot analysis of whole-cell lysates from cells treated with DMSO or tuvusertib (1 μM) over a 24-hour time course, demonstrating reduced ATM and DNA-PK compensation in PTEN-deficient cells following ATR inhibition. (**I**) Cell-cycle distribution of PTEN WT and PTEN-KO cells treated with DMSO, tuvusertib, or tuvusertib combined with ATM and DNA-PK inhibitors (ATMi + DNA-PKi). (**J**) CTG assay showing tuvusertib sensitivity in PTEN WT and PTEN-KO cells with or without ATMi + DNA-PKi. Data represent the mean ± SD.

The ATR/CHK1 pathway enforces a checkpoint at the S/G_2_ transition to ensure the completion of DNA replication, but compensation by ATM and DNA–dependent proteinase K (DNA-PK) is a common mechanism by which cells avoid lethality from ATR inhibition (42, 43). ATM and DNA-PK phosphorylate CHK1 at S317 and S345, similar to ATR, to limit cell-cycle progression and the accumulation of replication stress. Phosphorylation of CHK1 at S317 and S345 promotes the retention of CHK1 in the nucleus, supporting its cell-cycle checkpoint functions. In contrast, CHK1 is sequestered in the cytoplasm of PTEN-deficient cells, as loss of the lipid phosphatase activity of PTEN promotes AKT-mediated phosphorylation of CHK1 and its cytoplasmic localization (44). Consistent with this model of CHK1 localization, we observed greater enrichment of CHK1 in the cytoplasmic fraction in PTEN-KO OVCAR8 cells compared with PTEN WT cells (Figure 3A). However, it is unclear how ATR inhibition affects CHK1 localization and whether, in PTEN-deficient cells, PTEN loss or compensatory ATM or DNA-PK activities are dominant in determining CHK1 localization after ATR inhibition.

To determine whether the localization of CHK1 is altered by ATR inhibition in PTEN-KO cells, we subjected tuvusertib-treated PTEN-KO and WT cells to immunofluorescence, and the nuclear-to-cytoplasmic ratio of CHK1 was quantified using CellProfiler (Figure 3, B and C). The baseline nuclear-to-cytoplasmic ratio for CHK1 was significantly lower in PTEN-deficient cells. The nuclear-to-cytoplasmic ratio for CHK1 increased after tuvusertib treatment in both PTEN WT and -KO cells, although the degree of increase in PTEN-KO cells was significantly less than in PTEN WT cells. These results indicate that CHK1 nuclear localization after ATR inhibition was impaired by PTEN loss.

We evaluated CHK1 localization in PTEN-deficient cells reconstituted with WT or PTEN catalytic mutants to define the catalytic activities of PTEN underlying CHK1 localization (Supplemental Figure 3A). Reexpression of WT PTEN fully restored nuclear enrichment of CHK1 following ATR inhibition, but neither the catalytically inactive C124S mutant nor the lipid phosphatase–deficient G129E mutant rescued CHK1 nuclear localization. These findings indicate that the lipid phosphatase activity of PTEN was required to promote CHK1 nuclear retention and prevent its cytoplasmic sequestration during replication stress.

The relative reduction in nuclear CHK1 after tuvusertib treatment in PTEN-deficient cells compared with WT cells raised the possibility that PTEN loss compromised cell-cycle checkpoints in the absence of ATR signaling. ATR-CHK1–mediated checkpoint control at the S/G_2_ transition ensures completion of genome duplication before mitosis and prevents DNA replication, detected by BrdU incorporation, from occurring in high phospho–histone H3 (pHH3^hi^) mitotic cells. Consequently, when ATR signaling was inhibited, PTEN WT cells accumulated in the S phase, while PTEN-KO cells progressed to the G_2_/M phase (Figure 3, D and E), suggesting that the S/G_2_ transition was compromised in PTEN-deficient cells. Additionally, we found that there were more BrdU^+^ cells with high pHH3 levels in PTEN-deficient OVCAR8 cells after ATR inhibitor exposure (Figure 3F), indicating that these cells were entering mitosis without completing DNA replication, potentially leading to mitotic catastrophe.

To determine if the dysregulated cell-cycle progression observed in PTEN-KO cells after ATR inhibition coincided with impaired ATM- and DNA-PK–mediated compensation, we assessed the levels of pATM and pDNA-PK after tuvusertib treatment. PTEN-KO cells had reduced levels of pATM and pDNA-PK and increased levels of γH2AX after 24 hours of ATR inhibitor treatment compared with PTEN WT cells (Figure 3G), suggesting that ATM/DNA-PK–mediated compensation after ATR inhibition was compromised. In both PTEN WT and -KO cells, phosphorylation of the activating S345 site on CHK1 was absent 2 hours after ATR inhibition (Figure 3H). pCHK1 levels remained low in PTEN-KO cells during the course of ATR inhibitor exposure; in contrast, in PTEN WT cells, pCHK1 levels approached baseline levels after 16 hours of ATR inhibition. These results demonstrate that PTEN WT but not PTEN-KO cells were able to establish robust ATM and DNA-PK–mediated compensation during loss of ATR signaling.

To determine whether this impaired compensation by ATM and DNA-PK following ATR inhibition contributes to the cell-cycle checkpoint defect and ATR inhibitor hypersensitivity observed in PTEN-deficient cells, both ATM and DNA-PK were inhibited during tuvusertib treatment in PTEN WT and -KO cells. Inhibition of ATM and DNA-PK during tuvusertib treatment increased the G_2_/M population in PTEN WT cells to levels comparable to those observed in PTEN-KO cells (Figure 3I and Supplemental Figure 3B), indicating PTEN loss compromised ATM and DNA-PK–mediated compensation following ATR inhibition. In line with the altered cell-cycle distribution, inhibition of ATM and DNA-PK sensitized PTEN WT cells to tuvusertib, recapitulating the hypersensitive phenotype of PTEN-deficient cells (Figure 3J). In contrast, ATM and DNA-PK inhibition only minimally enhanced ATR inhibitor sensitivity in PTEN-KO cells, suggesting that this compensatory pathway was already compromised in the absence of PTEN. Taken together, these data suggest that PTEN loss prevented the nuclear localization of CHK1 after ATR inhibition, compromised the S/G_2_ cell-cycle checkpoint leading to premature mitotic entry, and impaired compensatory ATM and DNA-PK activity in response to a loss of ATR signaling.

### PTEN loss increases cell death and impairs cellular proliferation after combined ATR and PARP inhibition.

**Figure 4:**
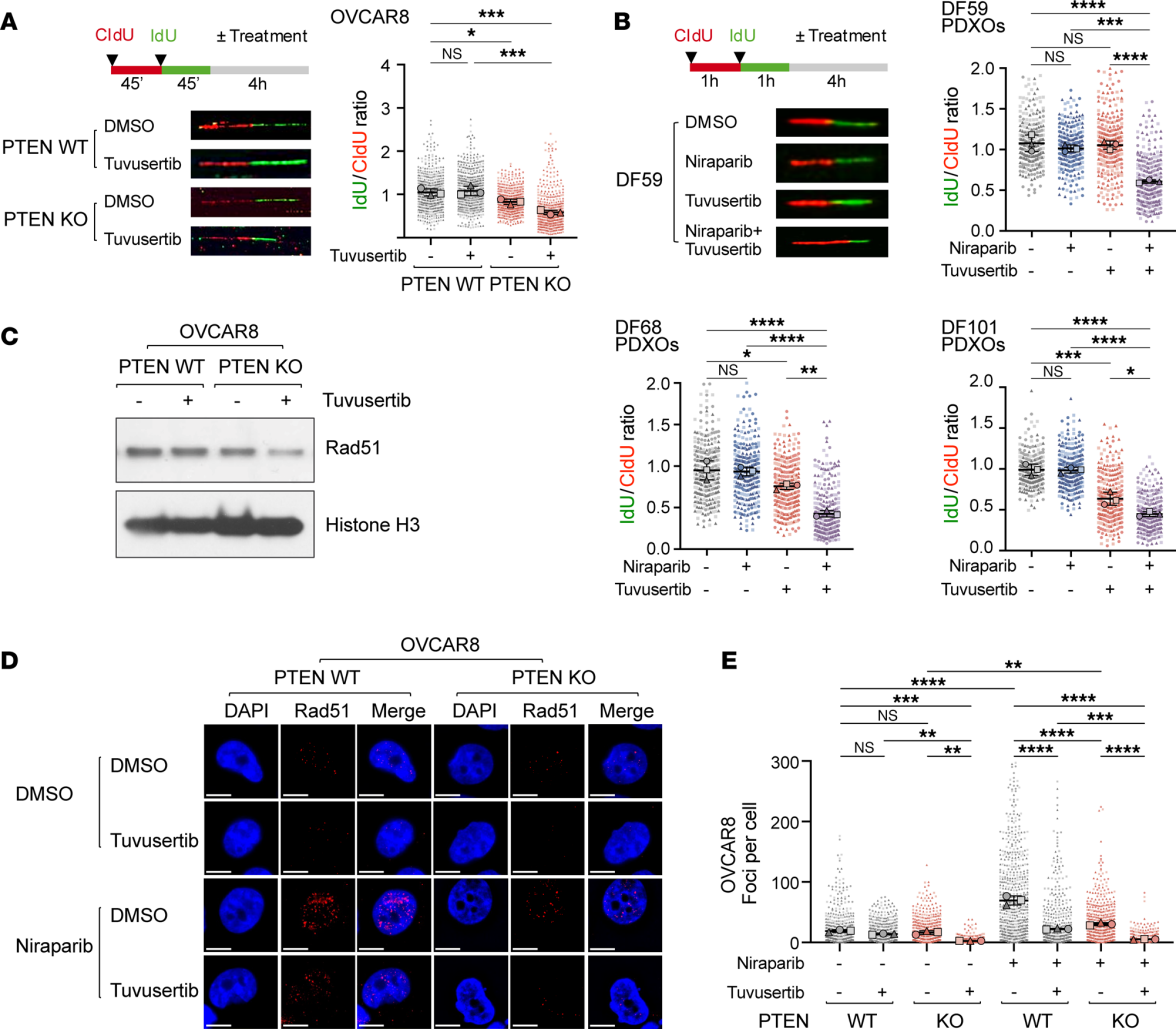
PTEN loss impairs RAD51-mediated fork protection following ATR inhibition. (**A**) DNA fiber assay assessing replication fork stability in PTEN WT and PTEN-KO cells. IdU and CldU track lengths were measured using ImageJ, and the IdU/CldU ratio was calculated to indicate fork stability. (**B**) DNA fiber assay in 3 organoid models treated with niraparib, tuvusertib, or their combination. ATR inhibition induced fork instability only in PTEN-deficient models. (**C**) Western blot analysis of chromatin-bound fractions from PTEN WT and PTEN-KO cells following ATR inhibitor treatment, showing reduced RAD51 accumulation in PTEN-deficient cells. (**D**) Immunofluorescence staining for RAD51 foci in OVCAR8 PTEN WT and PTEN-KO cells treated with DMSO, niraparib, tuvusertib, or their combination. Scale bars: 10 μm. (**E**) Quantification of RAD51 foci per cell. Each dot represents an individual DNA fiber (**A** and **B**) or cell (**E**) from 3 independent experiments. Symbols represent the mean value of each biological replicate. Data are presented as the mean ± SD (*n* = 3 independent experiments per group). At least 100 DNA fibers (**A** and **B**) or 200 cells (**E**) were analyzed per experiment. Statistical analyses were performed using biological replicate means and assessed by 1-way ANOVA with Tukey’s multiple-comparison test (*P < 0.05, **P < 0.01, ***P < 0.001, and ****P < 0.0001).

The increased G_2_/M progression in PTEN-KO cells after ATR inhibition raised the possibility that ATR inhibition could induce replication fork instability in PTEN-deficient cells, as mitotic kinase activity prior to the completion of DNA replication triggers replication fork disassembly and collapse (45). As demonstrated by DNA fiber assays, OVCAR8 PTEN-KO cells have lower replication fork stability at baseline compared with WT cells, with further compromise after ATR inhibition (Figure 4A). The effects of ATR inhibition also extended to *BRCA1*-mutant PARP inhibitor-resistant HGSOC organoid models that demonstrated stable replication forks at baseline. Tuvusertib monotherapy only compromised replication fork stability in the PTEN-deficient DF68 and DF101 models but did not compromise fork stability in the PTEN-proficient DF59 model (Figure 4B, tuvusertib monotherapy treatment vs. vehicle).

RAD51 is a CHK1 substrate that plays a critical role in stabilizing replication forks. Given that PTEN-deficient cells have less nuclear CHK1 and diminished CHK1 activation after ATR inhibition, we expected and observed a greater reduction in chromatin-associated RAD51 levels in both OVCAR8 and TYKNU PTEN-KO cells after tuvusertib treatment (Figure 4C and Supplemental Figure 4A).

To further evaluate RAD51 function, we assessed the formation of RAD51 foci in response to tuvusertib. Consistent with the Western blots of chromatin-associated RAD51 (Figure 4C and Supplemental Figure 4A), there were significantly fewer foci only in PTEN-KO cells after ATR inhibition (OVCAR8 RAD51 immunofluorescence shown in Figure 4D with quantification in Figure 4E, left 4 treatment groups; TYKNU quantification in Supplemental Figure 4B). Together, these findings suggest that PTEN loss impaired RAD51-mediated fork protection following ATR inhibition. These results also extended to the formation of RAD51 foci after PARP inhibitor exposure. In response to the PARP inhibitor niraparib, PTEN WT cells mounted a robust accumulation of RAD51 foci that was attenuated but not completely extinguished by ATR inhibition. In contrast, PTEN-KO cells exhibited diminished formation of RAD51 foci in response to PARP inhibition that were further reduced by the addition of ATR inhibition, resulting in a mean of fewer than 5 foci per cell (OVCAR8 shown in Figure 4, D and E, right-hand 4 treatment groups; TYKNU quantification in Supplemental Figure 4B). The loss of RAD51 foci after ATR inhibition in PTEN-KO cells suggests that ATR inhibition may sensitize PTEN-deficient cells to PARP inhibition to a greater degree than PTEN-expressing cells.

The markedly diminished formation of RAD51 foci in PTEN-KO cells following combined ATR and PARP inhibition is expected to translate to reduced replication fork stability. To test this possibility, we assessed fork stability in the PDXO models after treatment with tuvusertib combined with the PARP inhibitor niraparib. Replication fork stability, reduced by ATR inhibitor monotherapy, was further compromised in the PTEN-deficient DF68 and DF101 models by niraparib in the presence of tuvusertib. Notably, fork stability was also compromised by combination treatment in the DF59 model (Figure 4B, combined tuvusertib and niraparib treatments compared with controls).

We also examined ssDNA gap formation in response to these treatments. In ssDNA gap assays, only combination treatment, and not monotherapy, induced ssDNA gaps in the 3 organoid models, irrespective of PTEN expression (Supplemental Figure 4C). The induction of ssDNA gaps along with fork instability in the combination-treated samples suggests that combined ATR and PARP inhibitor therapy synergized through the dysregulation of multiple replication fork pathways.

**Figure 5:**
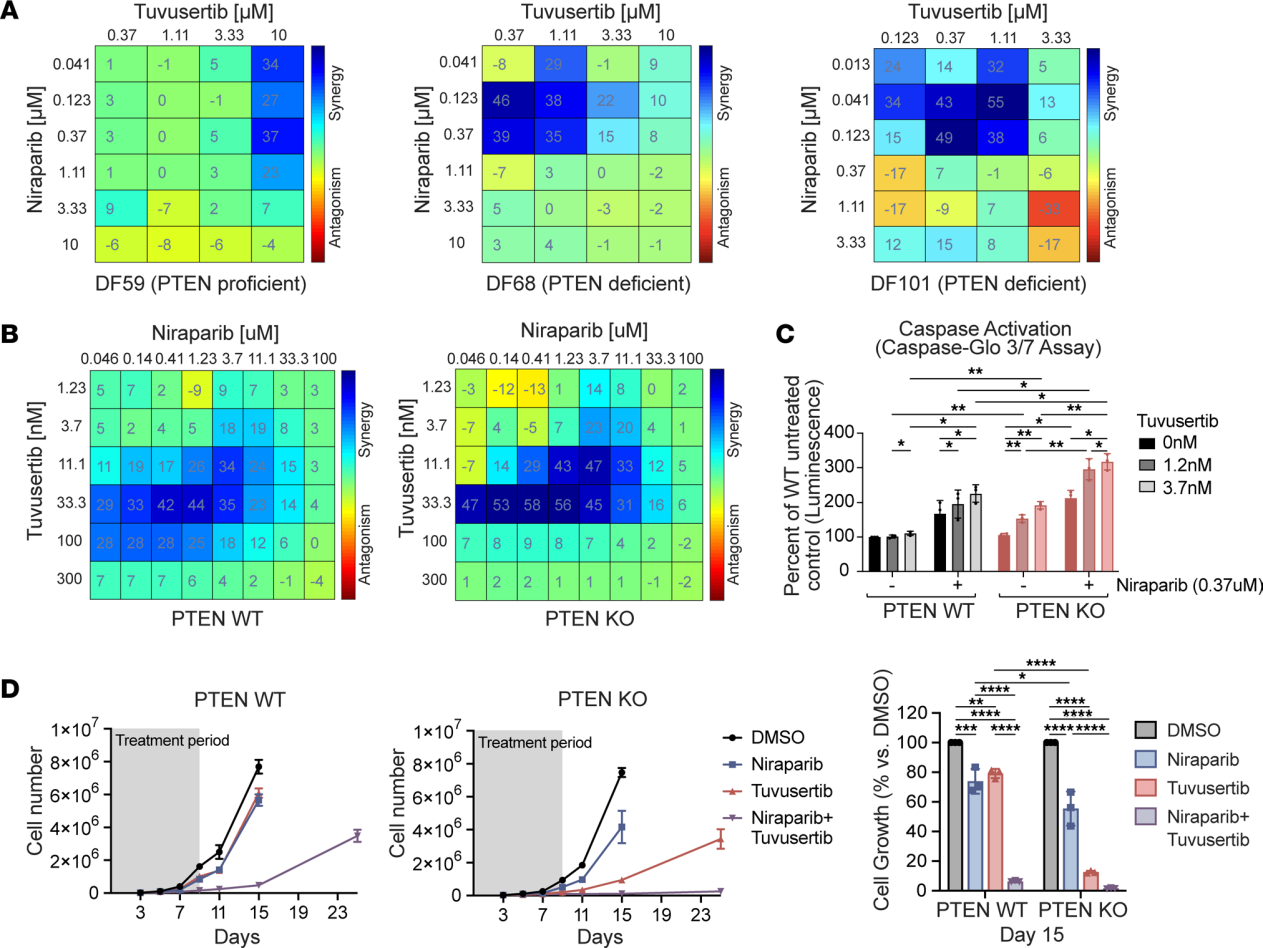
PTEN loss increases cell death and impairs cellular proliferation after combined ATR and PARP inhibition. (**A** and **B**) Bliss synergy plots of niraparib and tuvusertib in PDXO models and OVCAR8 cells. (**C**) Caspase activation assay quantifying apoptosis in OVCAR8 PTEN WT and PTEN-KO cells treated with tuvusertib (0, 1.2, 3.7 nM) with or without niraparib (0.37 μM). Luminescence intensity reflects caspase activity. Data represent the mean ± SD. Statistical significance was determined by 1-way ANOVA with Tukey’s post hoc test (*P < 0.05 and **P < 0.01). (**D**) Washout experiment in PTEN-proficient and PTEN-deficient cells. After 9 days of treatment, drugs were washed off, and cell regrowth was monitored until day 25. Cell numbers were counted at each time point. Data represent the mean ± SD. Statistical significance was determined by 1-way ANOVA with Tukey’s post hoc test (*P < 0.05, **P < 0.01, ***P < 0.001, and ****P < 0.0001).

Consistent with the biological effects on replication fork dynamics, tuvusertib and niraparib combined were synergistic across the models. However, in the PTEN-proficient DF59 model, synergism was only seen at high tuvusertib concentrations, whereas effects in the DF68 and DF101 PTEN-deficient models were observed at substantially lower concentrations (Figure 5A). Similarly, PTEN-deficient cell lines were also more responsive to combined ATR and PARP inhibition, with enhanced synergy in PTEN-KO OVCAR8 cells compared with PTEN WT cells (Figure 5B). The enhanced synergistic effects translated to a greater degree of apoptosis in response to tuvusertib alone or when combined with niraparib in PTEN-KO cells compared with WT cells (Figure 5C). Taken together, our results indicate that PTEN deficiency conferred sensitivity to ATR inhibition but also greater sensitivity to combined ATR and PARP inhibition in HGSOC models.

In addition to greater effects of combination treatment during drug exposure, we hypothesized that the greater degree of apoptosis may translate to more prolonged effects on tumor cell proliferation in PTEN-deficient versus -proficient cells. To assess this possibility, we conducted washout experiments in OVCAR8 PTEN WT and -KO cells and assessed cell number changes during the treatment period and after drug washout (Figure 5D). As shown previously, PTEN-deficient cells were more sensitive to ATR inhibitor monotherapy than were PTEN-proficient cells. After exposure to combination therapy, the growth of both PTEN-proficient and PTEN-deficient tumor cells was suppressed, but regrowth occurred quickly after treatment removal in PTEN-proficient cells, whereas the inhibitory effect on tumor cell proliferation persisted much longer in PTEN-deficient cells.

### PTEN deficiency confers increased sensitivity to ATR and combined ATR/PARP inhibition in vivo.

**Figure 6:**
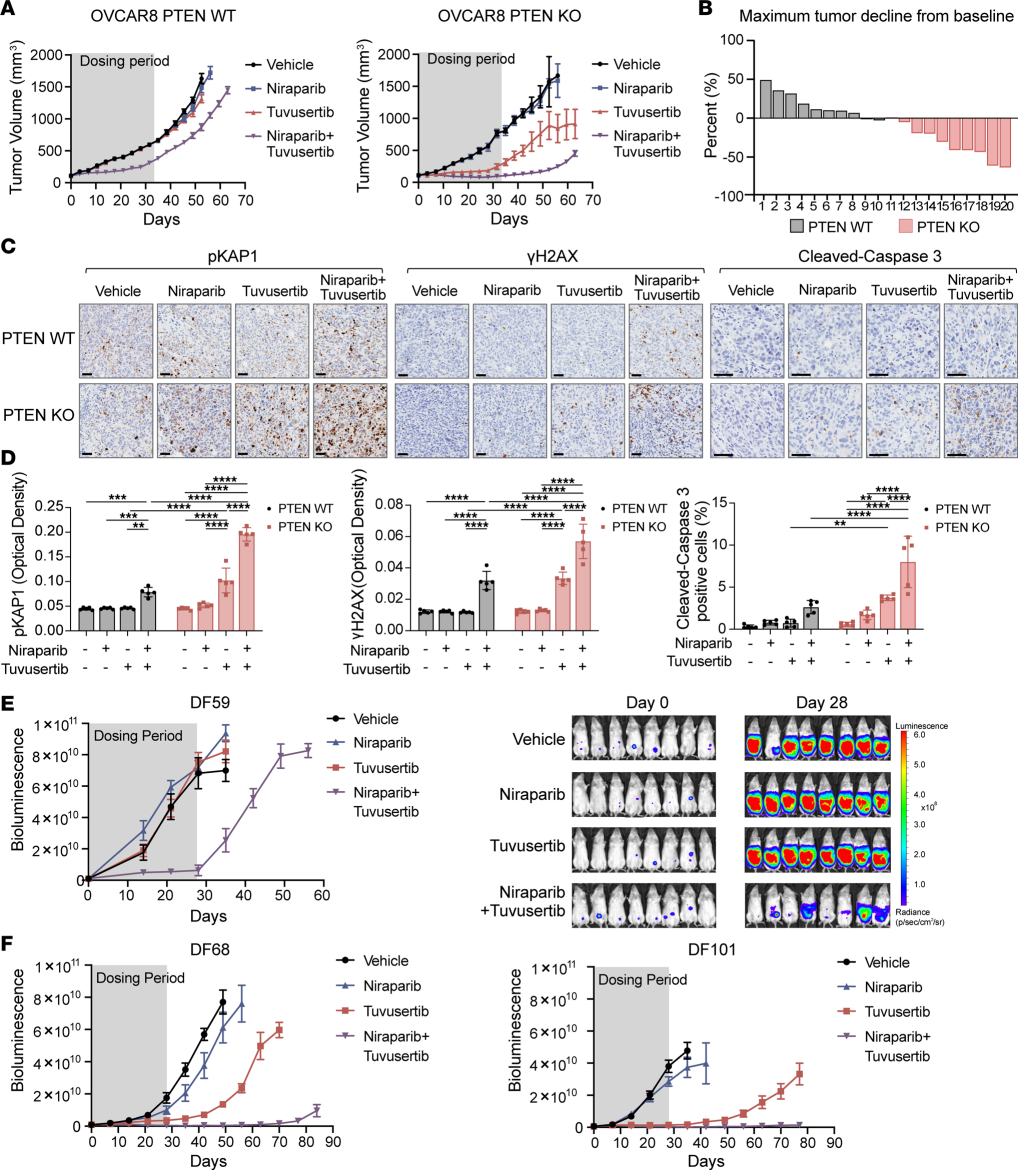
PTEN deficiency confers increased sensitivity to ATR and combined ATR/PARP inhibition in vivo. (**A**) In vivo xenograft study using OVCAR8 PTEN WT and PTEN-KO cells implanted into nude mice, treated with vehicle, niraparib (45 mg/kg), tuvusertib (25 mg/kg), or their combination for 28 days. PTEN-KO tumors exhibited increased sensitivity to ATR inhibition alone and substantial tumor regression with combination therapy. Tumor growth inhibition persisted longer in the PTEN-KO models after treatment cessation. Data represent the mean ± SEM. (**B**) Waterfall plot depicting tumor regression in PTEN WT and PTEN-KO groups after combination treatment. PTEN-KO tumors showed a greater size reduction, with 9 of 10 PTEN-KO mice showing tumor growth regression and 8 of 10 PTEN WT mice exhibiting tumor growth inhibition. (**C** and **D**) IHC staining of xenograft tumors for pKAP1, γH2AX, and cleaved caspase 3. Tuvusertib alone and in combination with niraparib induced higher levels of replication stress and DNA damage markers, as well as increased apoptosis, in PTEN-deficient tumors compared with PTEN-proficient tumors. Scale bars: 50 μm. Data represent the mean ± SD. Statistical significance was determined by 1-way ANOVA with Tukey’s post hoc test (**P < 0.01, ***P < 0.001, and ****P < 0.0001). (**E** and **F**) Efficacy of niraparib, tuvusertib, and their combination in PDX models with PTEN proficiency (DF59) and deficiency (DF68, DF101). PTEN-deficient models exhibited greater sensitivity to tuvusertib monotherapy and sustained tumor growth inhibition following treatment cessation. Data represent the mean ± SEM.

To evaluate the efficacy of combined ATR and PARP inhibition in vivo, we conducted 4-arm experiments using PTEN WT and -KO OVCAR8 xenografts (Figure 6A). Combination therapy resulted in profound tumor growth inhibition in both PTEN-proficient and -deficient tumors; however, regression was primarily observed in mice bearing PTEN-deficient tumors (Figure 6B). Nine of 10 PTEN-deficient xenografts showed a reduction in tumor size during exposure to the combination therapy, whereas 8 of 10 PTEN-proficient xenografts showed some degree of tumor growth. These results confirmed that PTEN-deficient tumors were more sensitive to ATR inhibitor monotherapy and combined ATR/PARP inhibition. Notably, tumor relapse occurred immediately after the dosing period in PTEN-proficient tumors, whereas antitumor effects of the combination therapy persisted much longer in PTEN-deficient xenografts.

Levels of pKAP1, γH2AX, and cleaved caspase 3 were analyzed in these tumors by IHC to assess replication stress, DNA damage, and apoptosis induced by these treatments (Figure 6, C and D). Tuvusertib alone and in combination with niraparib induced higher levels of pKAP1 and γH2AX in PTEN-deficient tumors compared with PTEN-proficient tumors. We confirmed these results by Western blotting, which also showed higher levels of pRPA at baseline and after ATR inhibitor monotherapy or combination treatment in PTEN-deficient tumors (Supplemental Figure 5). The increased replication stress and DNA damage observed in PTEN-deficient tumors translated to a greater degree of apoptosis in response to ATR inhibition, which was further increased in combination with PARP inhibition, as demonstrated by IHC for cleaved caspase 3 (Figure 6, C and D). As in our in vitro studies, the greater degree of induction of apoptosis in PTEN-deficient cells probably contributed to the persistent antitumor effects of combination treatment after the cessation of dosing (Figure 5D and Figure 6A).

We performed similar experiments using PTEN-proficient (DF59) or PTEN-deficient (DF68 and DF101) PDX models. Consistent with the results in organoid models derived from these PDXs, as well as with the OVCAR8 xenograft data, the PTEN-deficient models DF68 and DF101 showed greater sensitivity to tuvusertib monotherapy than did the DF59 PDX model (Figure 6, E and F). During the treatment period, combined tuvusertib and niraparib produced marked tumor growth inhibition in all 3 PDXs, so that the combination overcame resistance to both monotherapies in the DF59 model. However, upon cessation of dosing, DF59 tumors immediately resumed growth, whereas the PTEN-deficient models demonstrated prolonged tumor growth inhibition well beyond the treatment period. As with OVCAR8 xenografts, IHC showed that the PTEN-deficient DF68 and DF101 models had higher basal levels of pKAP1 and γH2AX, which were further increased by ATR inhibition or combined ATR and PARP inhibition (Supplemental Figure 6A). These findings were further confirmed by Western blotting of xenograft lysates (Supplemental Figure 6B). Additionally, combined treatment increased pKAP1 and γH2AX levels in the PTEN-proficient DF59 model, while PARP inhibition reduced PAR levels in both the DF59 monotherapy and combination therapy cohorts (Supplemental Figure 6, C and D). Consistent with our xenograft model results, the PTEN-deficient DF101 model exhibited a greater degree of apoptosis in response to tuvusertib, either as a monotherapy or in combination with niraparib, compared with the PTEN-proficient DF59 model (Supplemental Figure 6E).

### PTEN deficiency confers benefit to ATR inhibitor–based treatment in a clinical trial of combined ATR and PARP inhibition.

**Figure 7:**
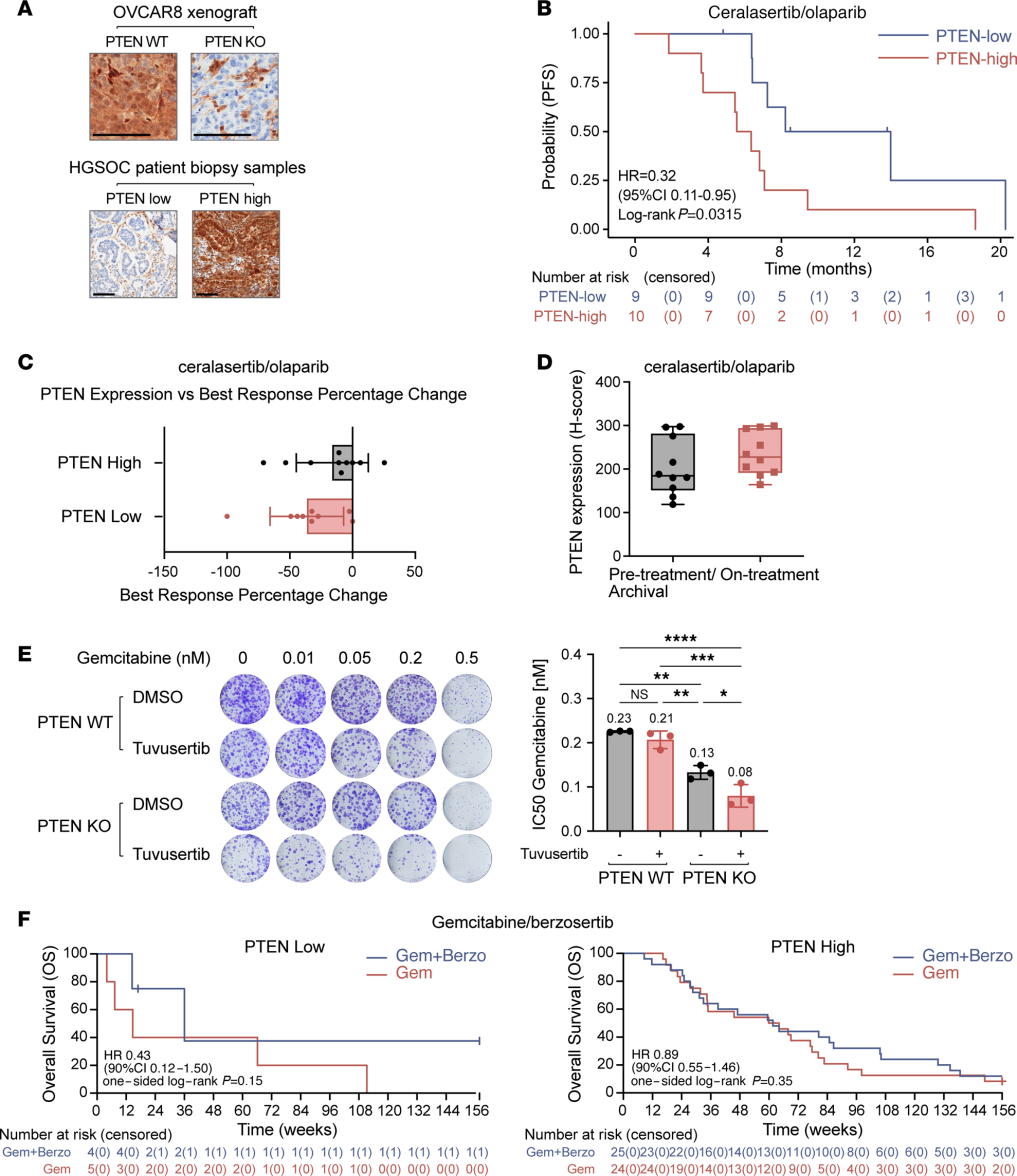
PTEN deficiency confers benefit to ATR inhibitor-based treatment in clinical trials. (**A**) Representative images of PTEN IHC in HGSOC patient biopsy samples. OVCAR8 WT and PTEN-KO xenograft tumors were used to optimize the staining protocol. Scale bars: 100 μm. (**B**) Kaplan-Meier survival curves from the CAPRI trial showing that patients with low PTEN expression had improved PFS. (**C**) Tumor regression percentages plotted for patients based on PTEN expression. Tumors with low PTEN levels showed greater reductions in size. (**D**) PTEN expression levels in 10 matched archival or pre- and on-treatment biopsy pairs, suggesting potential adaptive changes in response to combination therapy. (**E**) Colony formation assay showing that combining ATR inhibitor with gemcitabine significantly reduced cell viability in PTEN-KO cells compared with PTEN-WT cells. Statistical significance was determined by 1-way ANOVA with Tukey’s post hoc test (*P < 0.05, **P < 0.01, ***P < 0.001, and ****P < 0.0001). (**F**) Kaplan-Meier survival curves from a phase II study of gemcitabine (Gem) with or without the ATR inhibitor berzosertib (Berzo) in platinum-resistant ovarian cancer. Patients were stratified by PTEN expression levels. In patients with high PTEN expression, adding ATRi to gemcitabine did not improve overall survival, whereas in patients with low PTEN expression, the combination greatly improved survival compared with gemcitabine alone.

To explore whether patients with HGSOC expressing low levels of PTEN derive greater benefit from ATR inhibition than those with higher levels of expression, we analyzed PTEN expression in archival, pre-treatment and on-treatment biopsies in patients with platinum-sensitive HGSOC, irrespective of homologous recombination deficiency (HRD) status, who were treated with a combination of the ATR inhibitor ceralasertib combined with olaparib (Combination ATR and PARP Inhibitor [CAPRI]; NCT03462342) (46). PTEN staining was optimized using OVCAR8 WT and PTEN-KO cells (Figure 7A). Across 8 patients in whom we could compare archival and pre-treatment biopsies, there were no statistically significant differences in the levels of PTEN between the 2 groups. Among the 19 patients for whom archival or pre-treatment PTEN was assessable by IHC, those with tumors with lower PTEN expression had improved progression-free survival (PFS) compared with those with PTEN^hi^-expressing tumors (Figure 7B). In addition, patients with tumors expressing low levels of PTEN had a greater degree of tumor reduction (Figure 7C). Interestingly, in 10 matched archival or pre- and on-treatment biopsy pairs, PTEN expression levels tended to rise, suggesting a possible adaptation to combination therapy (Figure 7D). This adaptation also occurred in in vitro experiments in response to PARP or combined PARP and ATR inhibition (Supplemental Figure 7A). Taken together, these data suggest that patients with HGSOC with low PTEN expression experienced improved clinical benefit with combined ATR and PARP inhibitor treatment compared with those with higher PTEN levels.

### PTEN deficiency confers benefit to ATR inhibitor–based treatment in combination with gemcitabine.

ATR inhibition is also being developed as a strategy to exacerbate chemotherapy-induced replication stress. To determine whether low PTEN expression confers broader sensitivity to ATR inhibitor–based therapies beyond combined ATR and PARP inhibition, we tested the combination of tuvusertib and gemcitabine using OVCAR8 cells. In PTEN-KO OVCAR8 cells, tuvusertib significantly increased sensitivity to gemcitabine (Figure 7E). We did not observe this effect in PTEN WT cells. Furthermore, tuvusertib alone, and in combination with gemcitabine, induced higher levels of replication stress and DNA damage biomarkers, including pKAP1, pRPA, and γH2AX, in PTEN-deficient cells compared with PTEN-proficient cells (Supplemental Figure 7B). These findings suggest that ATR inhibition exacerbated gemcitabine-induced replication stress in PTEN-deficient cells, leading to cell death.

To investigate whether the observed replication stress is associated with aberrant DNA helicase activity and DNA polymerase-helicase uncoupling, we examined MCM2 pS41 and pS108 levels after tuvusertib and gemcitabine treatments. Ub-PCNA levels were increased in both PTEN WT and -KO cells upon combination therapy, indicating replication fork stalling (Supplemental Figure 7C). However, MCM2 pS41 levels failed to decrease following tuvusertib and gemcitabine combination treatments in PTEN-KO cells in contrast to PTEN WT cells. Furthermore, unlike in PTEN WT cells, MCM2 pS108 levels were not increased in PTEN-KO cells after combined tuvusertib and gemcitabine treatment. These differential effects on MCM2 phosphorylation suggest higher MCM2 activity after combined ATR inhibition with gemcitabine treatment in PTEN-deficient cells, similar to the MCM2 dysregulation observed with ATR inhibitor monotherapy (Figure 2A). Collectively, these findings suggest that PTEN-deficient cells had increased uncoupling of DNA polymerase and helicase, leading to more replication stress induced by ATR inhibition in combination with gemcitabine.

Finally, we assessed PTEN expression in archival samples from patients with platinum-resistant HGSOC enrolled in a randomized phase II trial of gemcitabine with or without the ATR inhibitor berzosertib (National Cancer Institute [NCI] CTEP 9944; NCT02595892), in which patients receiving the combination experienced a benefit in PFS and numerically improved overall survival (OS) (19, 20). Of the 70 patients enrolled, archival samples for PTEN IHC were available from 58 patients. For patients with tumors expressing high levels of PTEN, the addition of berzosertib did not significantly improve OS (HR, 0.89). In contrast, for patients with tumors expressing low levels of PTEN, there was a trend toward improvement in OS for those receiving the combination therapy (HR, 0.43; Figure 7F), suggesting that low PTEN levels may confer clinical benefit more broadly with ATR inhibitor–based therapies.

## Discussion

ATR inhibition has been considered a plausible strategy for ATM-deficient tumors for which preclinical data indicate synthetic lethality, or for tumors under a high degree of replication stress driving an ATR-dependent state for viability (24, 25, 47, 48). Despite this biology, the response rates to ATR inhibitor monotherapy have been low, due in part to difficulty in defining ATM deficiency, but also because of compensation by other phosphatidylinositol 3-kinase-related kinase family members, including ATM and DNA-PK (42, 43). Additionally, although ATR inhibitor–based combinations with PARP inhibition or gemcitabine have shown considerable promise in HGSOC (9, 19, 20, 46), overlapping hematologic toxicities have resulted in the use of attenuated doses. This highlights the importance of defining novel biomarkers of sensitivity for ATR inhibitor–based treatment, either as monotherapy or in combination, to improve patient selection strategies and to identify populations more likely to achieve clinical benefit even if reduced doses are used. Here, we have identified PTEN deficiency as a potential biomarker for ATR inhibitor sensitivity, either alone or in combination with PARP inhibition or gemcitabine. Our results corroborate a previously reported CRISPR screen that identified loss of *PTEN* expression among genes whose loss conferred ceralasertib sensitivity in mouse embryonic stem cells (29), and we build on these findings by demonstrating that loss of protein and lipid phosphatase activities of PTEN each contributed to the synthetic lethality between PTEN deficiency and ATR inhibition by distinct mechanisms.

Compared with PTEN-proficient counterparts, PTEN-deficient models exhibited higher basal levels of replication stress and DNA damage that were further exacerbated by ATR inhibition. Although both PTEN WT and -KO cells had fork stalling as denoted by Ub-PCNA levels, MCM2 in PTEN-deficient cells was neither dephosphorylated at the activating MCM2-dependent Ser41 site, nor phosphorylated at the inhibitory, ATR/ATM-dependent Ser108 site. Consequently, helicase and polymerase activities were uncoupled in PTEN-deficient cells following ATR inhibition, leading to successive helicase-mediated unwinding and excessive ssDNA, demonstrated by native BrdU and RPA immunofluorescence. This unconstrained fork progression provides a molecular basis for the chromosomal instability previously linked to nuclear PTEN loss (49), which is consistent with the higher basal levels of DNA damage observed in our PTEN-deficient models.

PTEN deficiency also impaired the cellular response to replication stress. PTEN-deficient cells had reduced nuclear CHK1 levels, leading to aberrant cell-cycle progression and impaired compensation by ATM and DNA-PK. Reduced ATM compensation probably contributed to the hyperactivation of MCM2, as Ser108 on MCM2 was phosphorylated in PTEN WT cells but not in PTEN-deficient cells, where ATM compensation was reduced. Importantly, the reduced activation of compensatory pathways mediated by ATM and DNA-PK in PTEN-deficient cells led to unresolved replication stress and increased cell death. Combination therapy therefore induced higher levels of apoptosis in PTEN-deficient cells, as evidenced by increased caspase activation and cleaved caspase 3 staining, translating to greater and more prolonged tumor regression in vivo.

ATR inhibition induced the hallmarks of PARP inhibitor sensitivity in PTEN-deficient cells, where it caused unstable forks and reduced RAD51 recruitment to chromatin. Notably, the relationship between PTEN deficiency, RAD51 expression, and PARP inhibitor monotherapy sensitivity is complex, and PTEN depletion has produced variable results in the sensitization of models of endometrial and prostate cancer to PARP inhibition (50–53). Nonetheless, irrespective of the PARP inhibitor monotherapy response, the antitumor activity of combined ATR and PARP inhibition was consistently accentuated in PTEN-deficient cells.

PTEN-deficient tumors may derive greater benefit from ATR inhibitor–based treatments than those with PTEN-proficient tumors based on the retrospective analyses we conducted of HGSOC trials utilizing ceralasertib combined with olaparib (CAPRI) or berzosertib combined with gemcitabine, underscoring the potential of PTEN as a biomarker for patient selection. In the CAPRI study, 1 patient with high expression of PTEN but prolonged PFS had a tumor harboring an *ATM* mutation and *AKT3* copy number gain, events that may have predisposed the individual to beneficial effects of ATR inhibition independent of the patient’s PTEN status. Even with inclusion of this patient, there was still a statistically significant difference in PFS between patients with tumors harboring low versus high expression.

If PTEN is considered a biomarker in future trials of ATR inhibitor–based treatment, in the case of HGSOC, IHC analysis will be critical, since only a minority (~6%) of tumors carry *PTEN* genomic alterations predicting complete PTEN protein loss (54), whereas loss of expression may occur in 19%–38% of cases (55, 56) through diverse mechanisms, including epigenetic silencing. Although our in vitro models utilized genetic deletion to define the underlying mechanism, our retrospective clinical cohorts capture this broader population by using IHC to assess PTEN levels, supporting the concept that functional PTEN depletion, whether genomic or epigenetic, drives a shared therapeutic vulnerability to ATR inhibition. In our retrospective analyses of the ceralasertib/olaparib and gemcitabine/berzosertib trials, we used cohort-specific H-scores to dichotomize low and high expressors of PTEN. In the gemcitabine/berzosertib study, archival samples were used, whereas in the ceralasertib/olaparib trial, pre-treatment biopsies were obtained after prior chemotherapy exposure. Since PTEN expression may change as an adaptation to replicative stress, H-scores defining relative PTEN deficiency may vary depending on prior treatment and may need to be assessed in significantly larger cohorts than those examined here.

It will be of interest to determine whether our results extend to other cancer types, including endometrial cancer, prostate cancer, triple-negative breast cancer, and leiomyosarcoma, in which the rates of *PTEN* mutation and deep deletion are higher (57), and in which ATR inhibitor–based treatments are also of significant interest in both HR repair–proficient and –deficient populations. In fact, 2 PTEN-deficient triple-negative breast cancer cell lines have been reported to have a lower IC_50_ with a greater degree of DNA damage following exposure to the ATR inhibitor VE-821 than a PTEN-proficient line (58). Furthermore, assessment of ATR inhibitor–based treatment in PTEN-deficient immunocompetent models will also be important, as PTEN loss has been associated with immunosuppression mediated in part by tumor-associated macrophages (59). Considering the improved outcomes of patients with PTEN-deficient tumors in the berzosertib and ceralasertib trials, it is likely that ATR inhibitor–based combinations contribute to favorable reprogramming of the immune microenvironment (60).

In summary, our data indicate that PTEN deficiency may define a population of patients with HGSOC as particularly appropriate for ATR inhibitor–based treatments. As advancement of approaches for PARP inhibitor– and platinum-resistant disease represent priorities of high unmet medical need, our results suggest that ATR inhibition could be further developed using PTEN status in a biomarker-driven strategy that can be validated in prospective clinical trials.

## Methods

### Sex as a biologic variable.

All animal experiments were conducted using female mice, as ovarian cancer occurs exclusively in women.

### Reagents and antibodies.

All reagents and antibodies used are listed in Supplemental Table 1.

### Cancer cell lines and cell culture.

All cell lines were purchased from the American Type Culture Collection (ATCC) or the Japanese Collection of Research Biosources. Cell line authenticity was verified by short tandem repeat (STR) profiling. To prevent mycoplasma contamination, cells were routinely tested using a PCR-based mycoplasma detection kit (ABM, catalog G238).

Cells were cultured at 37°C with 5% CO_2_. OVCAR8 cells were maintained in DMEM (Gibco, Thermo Fisher Scientific, catalog 11965) supplemented with 10% FBS and 1% penicillin/streptomycin (10,000 units/mL penicillin and 10,000 μg/mL streptomycin; Gibco, Thermo Fisher Scientific). All other cell lines were grown in RPMI-1640 medium (Gibco, Thermo Fisher Scientific, catalog 11875) with 10% FBS and 1% penicillin/streptomycin.

PTEN-KO cell lines, including OVCAR8, TYKNU, and PEO4, were generated using the CRISPR/Cas9 system. Cells were transfected with sgRNA (Thermo Fisher Scientific, catalog A35533) and Cas9 (Thermo Fisher Scientific, catalog A36497) using Lipofectamine CRISPRMAX Cas9 Transfection Reagent (Thermo Fisher Scientific, catalog CMAX00003), following the manufacturer’s instructions.

For PTEN reexpression, cells were transduced with lentiviral vectors carrying either WT or mutant PTEN. Stable cell lines were selected and maintained in the presence of puromycin.

### Patient-derived and cell line xenograft studies.

All ascites-derived ovarian PDX models were developed, luciferized, and maintained at the DFCI, as previously described (32).

For PDX tumor studies, 8-week-old female NSG (NOD.Cg-*Prkdc^scid^ Il2rg^tm1Wjl^*/SzJ) mice were obtained from The Jackson Laboratory. Mice received i.p. injections of approximately 5 × 10^6^ luciferized tumor cells. Tumors were allowed to establish over 4–5 weeks, and animals demonstrating increasing bioluminescent imaging (BLI) signals were randomized into 4 treatment groups with 8 mice per group. Disease progression in the peritoneal cavity was monitored weekly via BLI. Niraparib was formulated in 0.5% methylcellulose (4,000 cP) and 0.5% Tween 80 in water and administered orally at 45 mg/kg daily for 4 weeks. Tuvusertib, prepared in 15% Captisol in water, was given orally at 25 mg/kg daily for the same duration.

For the OVCAR8 xenograft model, 6- to 8-week-old female nude mice (NU/J, The Jackson Laboratory, stock no. 002019) were used. A total of 1 × 10^6^ OVCAR8 cells were suspended in 100 μL of a 1:1 Matrigel-PBS mixture and injected s.c. Once tumors reached approximately 100 mm³ in size, mice were randomized into 4 treatment cohorts with 10 mice per group. Tumor growth was measured twice weekly using calipers. Drug formulations and dosing were identical to the PDX studies.

### PDX organoid studies.

Tumor cells from PDXs were pelleted by centrifugation at 400*g* for 5 minutes. In cases of RBC contamination, cells were treated with ACK lysis buffer, rinsed with basal culture media, and recentrifuged. The processed cell pellets were subsequently mixed with growth factor–reduced Matrigel. PDXOs were grown according to the protocol described previously (61).

### Cell viability assay.

A total of 500–1,000 cells per well were plated in 100 μL media in a 96-well plate and allowed to adhere overnight before treatment with DMSO or drugs. Following 5 days of treatment, viability was assessed using the CellTiter-Glo assay (Promega) according to the manufacturer’s instructions. Relative cell viability was normalized to the DMSO-treated control. Bliss synergy and antagonism analysis were performed using Combenefit software.

### 3D cell viability assay for ovarian PDXOs.

The drug sensitivity of PDXO models was assessed using the Cell Titer-Glo 3D Cell Viability Assay following a 7-day drug treatment period. The detailed protocol for the 3D viability assay has been described previously (61, 62).

### Colony formation assay.

Cells (500–2,000 per well) were plated in 6-well plates and treated as indicated. After approximately 14 days of incubation, colonies were fixed with cold methanol for 20 minutes, stained with 1% crystal violet in water for 30 minutes, and rinsed with tap water. Plates were scanned, and colony quantification was performed using ImageJ software. Dose-response curves and IC_50_ values were generated using GraphPad Prism (GraphPad Software).

### Western blot analysis.

Cells were scraped, pelleted, and lysed in RIPA buffer (Boston BioProducts) or cell lysis buffer (Cell Signaling Technology, 9803S) containing protease and phosphatase inhibitor cocktails (Calbiochem). Lysates were incubated on ice for 30 minutes and centrifuged at 15,000*g* for 15 minutes. Protein concentration was determined using the Pierce BCA assay kit (Thermo Fisher Scientific). Proteins were separated by gel electrophoresis and transferred onto PVDF membranes. Membranes were blocked with 5% milk and incubated overnight at 4°C with primary antibodies. After washing with TBST, membranes were incubated with HRP-conjugated anti-mouse or anti-rabbit secondary antibodies for 1 hour at room temperature. Blots were washed with TBST, and chemiluminescence was detected using x-ray films or the Luminescent Image Analyzer (Fujifilm).

### Immunofluorescence.

Cells were plated on sterilized coverslips and cultured for 24 hours before drug treatment. After treatment, cells were fixed with 4% paraformaldehyde for 20 minutes and permeabilized with 0.1% Triton X-100 for 15 minutes. For pre-extraction, cells were treated with 0.2% Triton X-100 for 1 minute before fixation. Subsequently, cells were blocked with 5% normal goat serum in 5% BSA for 1 hour and then incubated with primary antibodies overnight at 4°C. The next day, cells were washed 3 times with PBS and stained with FITC- and Texas Red–conjugated secondary antibodies (Jackson Immunoresearch Laboratories), along with DAPI diluted in 5% BSA. Coverslips were mounted using VECTASHIELD Mounting Medium (Vector Laboratories) and sealed with nail polish. Cells were visualized using a THUNDER fluorescence microscope (Leica Microsystems). Cell segmentation and fluorescence intensity quantification were performed using CellProfiler image analysis software.

### Flow cytometry.

Trypsinized cells were washed with PBS and fixed in 4% formalin in PBS for 15 minutes at room temperature. Following fixation, cells were washed 3 times with 1% BSA in PBS and permeabilized with 70% ethanol at –20°C for 30 minutes or overnight. After permeabilization, cells were washed 3 times with 1% BSA in PBS. To expose incorporated BrdU, samples were incubated with 200 μg/mL DNase I in DPBS for 1 hour at 37°C. Subsequently, cells were washed 3 times with 1% BSA in PBS and incubated overnight at 4°C with conjugated antibodies diluted in 1% BSA in PBS. After an additional 3 washes with 1% BSA in PBS, DNA staining was performed by incubating the samples in the dark at room temperature for 1 hour with a staining solution containing 1 μg/mL DAPI and 100 ng/mL RNase A. Samples were analyzed using a BD Fortessa flow cytometer, capturing 50,000 events per sample. Flow cytometric data were processed and analyzed with FlowJo software.

### DNA fiber assay in cell culture samples.

DNA fiber assays were performed using the Genomic Vision FiberPrep DNA Extraction Kit, FiberComb Molecular Combing System, and FiberVision Scanner. Replication fork stability in OVCAR8 cells was assessed as previously described (61, 62). Briefly, 200,000 cells per well were plated in 6-well plates and cultured for 24 hours prior to sequential labeling with 5-chloro-2′-deoxyuridine (CldU) for 45 minutes, followed by 5-iodo-2′-deoxyuridine (IdU) for 45 minutes. After labeling, cells were treated with the indicated drugs for 4 hours, with 3 PBS washes between steps. DNA fiber preparation was conducted according to the Genomic Vision FiberPrep DNA extraction protocol. Cells were embedded in low-melting-point agarose plugs, treated with proteinase K, and subjected to agarose digestion. The resulting DNA samples were combed onto silanized coverslips using the FiberComb Molecular Combing System. CldU and IdU incorporation was detected using a rat anti-BrdU antibody and a mouse anti-BrdU antibody, respectively, followed by secondary staining with a goat anti–rat Cy5 antibody and a goat anti–mouse Cy3 antibody respectively. DNA fibers were visualized using the FiberVision Scanner and quantified with ImageJ software.

### DNA fiber assay in organoid samples.

To assess replication fork stability in organoid samples, viable organoids were processed for partial Matrigel removal. Collected organoids were incubated on ice for 15 minutes to facilitate Matrigel melting, followed by centrifugation at 400*g* for 5 minutes at 4°C. If Matrigel removal was incomplete, samples underwent an additional 10-minute ice incubation, followed by repeat centrifugation with further Matrigel removal by discarding the supernatant. Organoids were sequentially labeled with CldU for 1 hour and IdU for 1 hour, followed by drug treatment for 4 hours. The DNA fiber assay was performed as previously described for monolayer cultures (61, 62).

Post-replicative ssDNA gaps were detected by a DNA fiber assay using the ssDNA-specific enzyme S1 nuclease, as described previously (61, 62). For the S1 nuclease assay, Matrigel was partially removed as described above. Organoids were then labeled with CldU for 1 hour, followed by IdU for 4 hours concurrently with drug treatment. After each labeling step, samples were washed 3 times with PBS. To remove residual Matrigel, samples were incubated on ice for 10 minutes before the final PBS wash. Samples were centrifuged at 400*g* and resuspended in 45 μL PBS. Plug preparation and overnight incubation were conducted according to the monolayer protocol. On the following day, samples were washed twice with washing buffer, followed by 1 wash with S1 buffer and an additional wash with S1 buffer with or without S1 nuclease. The remaining steps were performed as described above for monolayer samples.

### IHC.

Tumor cells derived from ascites in PDX models, and s.c. tumors from OVCAR8 xenograft models were collected, rinsed with saline, and fixed in 4% neutral buffered formalin for either 10 minutes or 24 hours. After fixation, cells were washed with PBS, embedded in Histogel (Richard Allen Scientific), processed into paraffin blocks, and sectioned for IHC analysis. Tumor chunks were embedded in paraffin and sectioned. Paraffin-embedded blocks from archival tissue and pretreatment biopsy specimens from patients enrolled in the CAPRI trial were similarly sectioned for IHC. Processing, staining, image scanning and quantification of preclinical and clinical samples were optimized and conducted at the Brigham and Women’s Hospital Specialized Histopathology Core.

### Statistics.

GraphPad Prism (GraphPad Software) was used to generate graphs and conduct statistical analyses for viability, growth curves, DepMap data, DNA fiber assays, immunofluorescence and immunohistochemical quantifications, flow cytometry, and clinical data. All data are represented as the mean ± SD unless indicated otherwise. Significance was tested using 1-way ANOVA with Tukey’s post hoc test unless indicated otherwise. A *P* value of less than 0.05 was considered statistically significant. The specific statistical tests and *P* values are provided in the figure legends.

### Study approval.

All animal experiments were conducted at DFCI with IACUC approval and in compliance with AAALAC accreditation standards. All procedures adhered to the ethics guidelines established by DFCI’s IACUC. The CAPRI study (NCT03462342) was approved by the University of Pennsylvania IRB as the IRB of record, with Dana-Farber and Johns Hopkins IRBs deferring to the University of Pennsylvania IRB via reliance agreements. The gemcitabine/berzosertib study (CTEP 9944; NCT02595892) was approved by the National Cancer Institute Central IRB (CIRB) to which all participating sites in the Experimental Therapeutics Clinical Trials Network (ETCTN) defer. All patients provided written informed consent for participation in these clinical trials and for procurement of protocol-related biopsies or archival tissue.

### Data availability.

Values for all data points in graphs are reported in the Supporting Data Values file. Data are available upon request.

## Author contributions

JH and GIS designed the project and wrote the manuscript. KP, ADDA, and GIS supervised experiments. JH performed most experiments and biological analyses. BK developed and performed histopathological assessments. OSS developed organoid cultures, and PCG maintained and treated PDX models. TBB, RL, HJ, AABAdC, YJ, JZY, DBM, GS, and SM assisted with the conduct of experiments. SJS, SCC, and DN provided biostatistical support. IH assisted with project management and data curation. PAK led the gemcitabine/berzosertib clinical trial. JFL, SG, RLG, LM, JT, NL, and FS recruited patients to the CAPRI clinical trial, led by FS. All authors reviewed, edited and approved the manuscript. GIS was responsible for the overall conduct of the study.

## Conflict of interest

PAK has served on Advisory Boards for AstraZeneca, GSK, Merck, Immunogen, EMD Serono, Scorpion, Schrodinger, Nimbus, and Mural Oncology. He has roles on an AstraZeneca Scientific Steering Committee and a Gilead Data Monitoring Committee. He has institutional funding as Principal Investigator on trials sponsored by AstraZeneca, Bayer, Eli Lilly, GSK, Merck & Co., Merck KGaA/EMD Serono, and Pfizer. ADD consults for AstraZeneca, Bayer AG, Bristol Myers Squibb, EMD Serono, GlaxoSmithKline, Impact Therapeutics, PrimeFour Therapeutics, Tango Therapeutics, Deerfield Management Company, Servier Bio-Innovation LLC, Roche Pharma, and Covant Therapeutics. He is an advisory board member for Impact Therapeutics and reports receiving commercial research grants from Merck KGaA/EMD Serono, Bristol Myers Squibb, Moderna, and Tango Therapeutics. GIS reports research funding from Merck KGaA/EMD-Serono, Tango Therapeutics, Bristol Myers Squibb, Merck & Co., Artios, Eli Lilly, and Pfizer. He has served on advisory boards for Merck KGaA/EMD-Serono, Circle Pharmaceuticals, Concarlo Therapeutics, Schrodinger, FoRx Therapeutics, and Xinthera. GIS also holds patents entitled “Dosage regimen for sapacitabine and seliciclib” and “Compositions and Methods for Predicting Response and Resistance to CDK4/6 inhibition.”

## Funding support

This work is the result of NIH funding, in whole or in part, and is subject to the NIH Public Access Policy. Through acceptance of this federal funding, the NIH has been given a right to make the work publicly available in PubMed Central.

- Dana-Farber/Harvard Cancer Center Specialized Program of Research Excellence (SPORE) in Ovarian Cancer NIH grant P50 CA240243 (to ADD and GIS).
- Johns Hopkins University/University of Pennsylvania SPORE in Ovarian Cancer NIH grant P50 CA228991 (to FS).
- Ludwig Center at Harvard (to GIS).
- NIH grant UM1 CA186709 (to GIS).
- Research Agreement between the DFCI and EMD Serono (CrossRef Funder ID: 10.13039/100004755, to ADD and GIS).

## Supplementary Material

Supplemental data

Unedited blot and gel images

Supporting data values

## Version 1 07/07/2026

In-Press Preview

## Version 2 08/17/2026

Electronic publication

## Version 3 08/17/2026

Corrected funding sources
