# Supplementary material for: PTEN deficiency confers sensitivity to ATR inhibitor–based treatment in high-grade serous ovarian cancer
Source: J Clin Invest. 2026 Jul 7;136(16):e197645. doi: 10.1172/JCI197645 (PMC13476146; doi:10.1172/JCI197645)
Supplement: Supplementary file 1 [file jci-136-197645-s008.pdf]

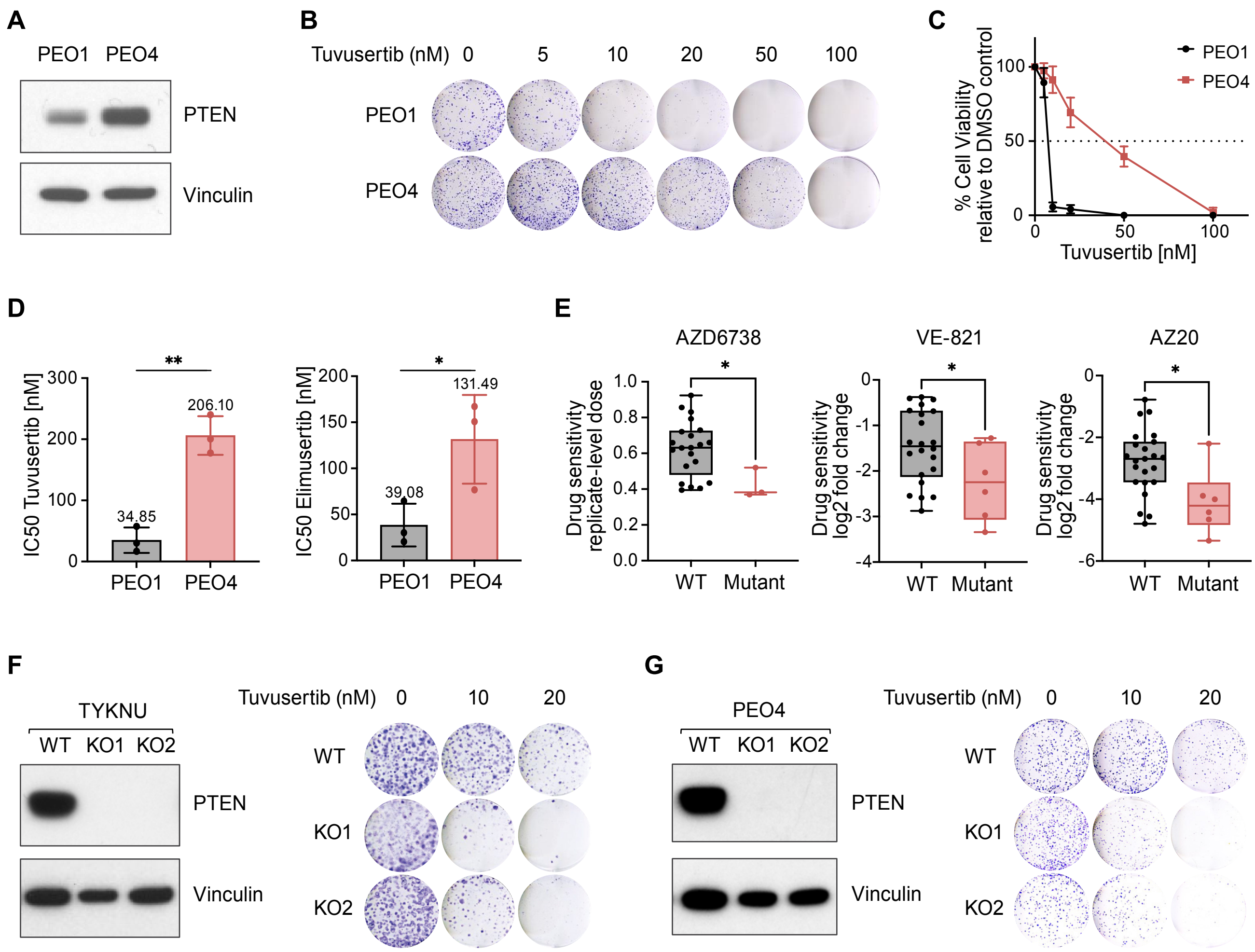

Supplemental Figure 1. ATR inhibitor sensitivity correlates with PTEN expression, and PTEN deficiency confers sensitivity to ATR inhibition. (**A**) Western blot analysis of PTEN expression in PEO1 and PEO4 cells. (**B** and **C**) Colony formation assays demonstrating that PTEN-high PEO4 cells are more resistant to tuvusertib than PTEN-low PEO1 cells. (**D**) CellTiter-Glo (CTG) assay showing increased sensitivity of PEO1 cells compared with PEO4 cells to the ATR inhibitors tuvusertib and elimusertib. Data are presented as mean ± SD. Statistical significance was determined using an unpaired Student's t test (**P* < 0.05, ***P* < 0.01). (**E**) Drug sensitivity of ovarian cancer cell lines to multiple ATR inhibitors using data from the DepMap database. Ovarian cancer cell lines harboring PTEN alterations exhibited increased sensitivity to ATR inhibition. Statistical significance was determined using an unpaired Student's t test (**P* < 0.05). (**F**) Western blot analysis confirming loss of PTEN expression in CRISPR-generated TYKNU PTEN-KO cells. Colony formation assays demonstrate increased sensitivity of PTEN-KO cells to tuvusertib. (**G**) Western blot analysis confirming loss of PTEN expression in CRISPR-generated PEO4 PTEN-KO cells. Colony formation assays demonstrate increased sensitivity of PTEN-KO cells to tuvusertib.

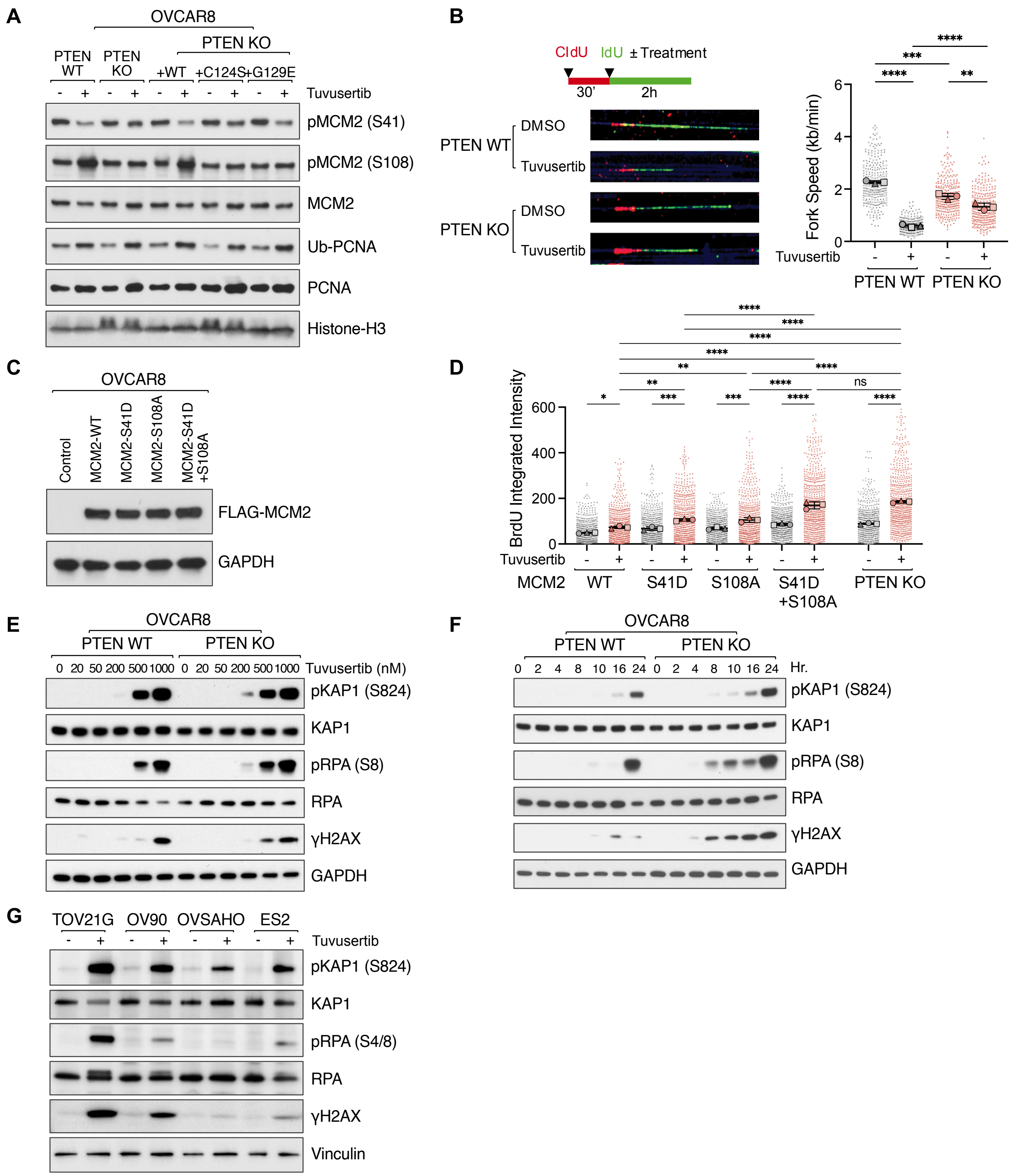

Supplemental Figure 2. PTEN deficiency enhances replication stress and DNA damage following ATR inhibition. (**A**) Western blot analysis of chromatin-bound fractions from OVCAR8 PTEN-WT, PTEN-KO, and PTEN-KO cells reconstituted with WT PTEN, C124S, or G129E PTEN mutants following ATR inhibitor treatment. Expression levels of phosphorylated MCM2 (pMCM2 S41 and pMCM2 S108) and ubiquitinated PCNA (Ub-PCNA) are shown. (**B**) DNA fiber assay measuring replication fork speed in PTEN WT and PTEN KO cells. Cells were labeled with CldU for 30 minutes, followed by IdU in the presence or absence of drug treatment for 2 hours. IdU tract length was measured to calculate fork speed. Tuvusertib-induced fork slowing was less pronounced in PTEN-deficient cells. (**C**) Western blot analysis confirming expression of exogenous FLAG-tagged WT MCM2 and MCM2 mutants in OVCAR8 cells. (**D**) Quantification of native BrdU fluorescence intensity in OVCAR8 cells expressing WT or mutant MCM2 following tuvusertib treatment. Each dot represents an individual DNA fiber (B) or cell (D) from three independent experiments. Symbols represent the mean value of each biological replicate. Data are presented as mean ± SD (n = 3 independent experiments per group). At least 100 DNA fibers or cells were analyzed per experiment. Statistical analyses were performed using biological replicate means and assessed by one-way ANOVA with Tukey's multiple-comparisons test. (**P* < 0.05, ***P* < 0.01, ****P* < 0.001, *****P* < 0.0001). (**E**) Dose-dependent changes in replication stress and DNA damage markers following ATR inhibition, as determined by Western blot analysis. (**F**) Time-course analysis of replication stress and DNA damage markers in OVCAR8 cells following ATR inhibition. (**G**) Western blot analysis of lysates from ovarian cancer cell lines treated with tuvusertib, assessing replication stress markers (pKAP1 and pRPA) and the DNA damage marker γH2AX.

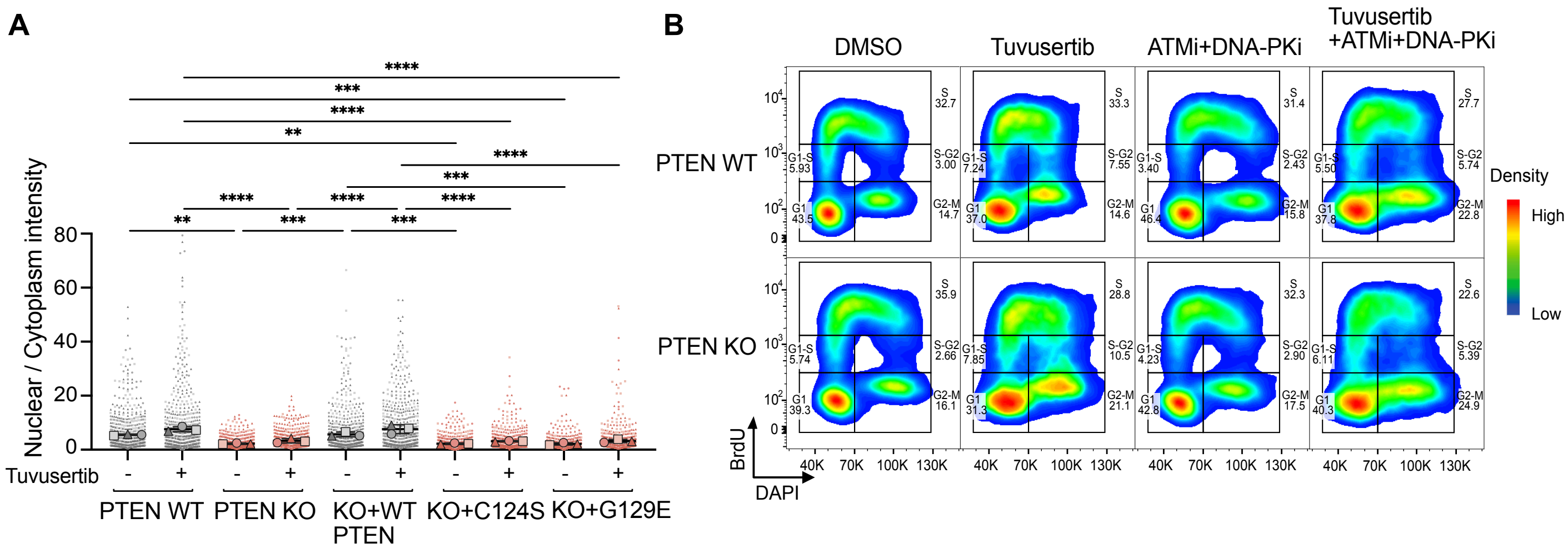

Supplemental Figure 3. PTEN lipid phosphatase activity regulates CHK1 localization, while ATM and DNA-PK enforce the S/G2 checkpoint under ATR inhibition. (**A**) Quantification of CHK1 nuclear-to-cytoplasmic fluorescence intensity ratios using CellProfiler in PTEN-WT, PTEN-KO, and PTEN-KO cells reconstituted with WT, C124S, or G129E PTEN mutants. Each dot represents an individual cell from three independent experiments, with at least 200 cells analyzed per experiment. Symbols represent the mean of each biological replicate. Data are presented as mean ± SD (n = 3 independent experiments per group). Statistical significance was assessed using biological replicate means by one-way ANOVA with Tukey's multiple-comparisons test. (**$P < 0.01$, ***$P < 0.001$, ****$P < 0.0001$). (**B**) PTEN-proficient and PTEN-deficient cells were treated with tuvusertib (1 μM), with or without ATM and DNA-PK inhibitors (ATMi + DNA-PKi), for 24 hours and pulsed with BrdU (10 μM) during the final 30 minutes. Representative flow cytometry plots of BrdU incorporation and DAPI staining are shown.

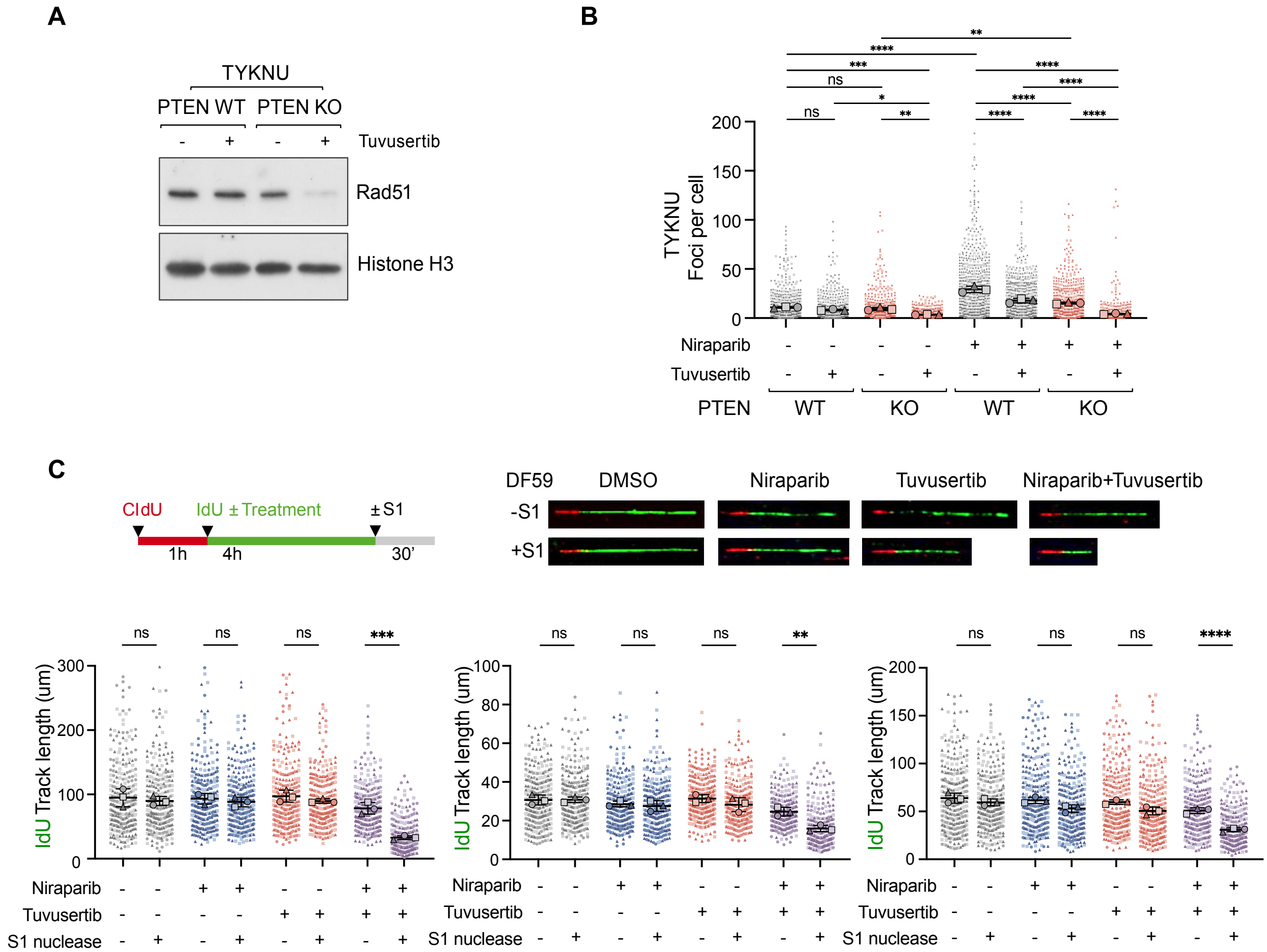

Supplemental Figure 4. PTEN deficiency impairs RAD51 chromatin loading, and combined ATR and PARP inhibition induces ssDNA gaps. (**A**) Western blot analysis of chromatin-bound fractions from PTEN-WT and PTEN-KO cells following ATR inhibitor treatment, showing reduced RAD51 accumulation in PTEN-deficient cells. (**B**) Quantification of RAD51 foci per cell in TYKNU PTEN-WT and PTEN-KO cells treated with niraparib, with or without tuvusertib. Tuvusertib markedly reduces RAD51 foci formation in PTEN-deficient cells. (**C**) DNA fiber assays with and without S1 nuclease treatment in patient-derived organoids. DNA labeling was performed sequentially with the nucleotide analogs CldU and IdU in the presence or absence of drugs, followed by treatment with or without S1 nuclease. Lower panels show quantification of IdU tract lengths to assess the presence or absence of ssDNA gaps. Each dot represents an individual cell (B) or DNA fiber (C) from three independent experiments. Symbols represent the mean value of each biological replicate. Data are presented as mean ± SD (n = 3 independent experiments per group). At least 200 cells (B) or 100 DNA fibers (C) were analyzed per experiment. Statistical analyses were performed using biological replicate means and assessed by one-way ANOVA with Tukey's multiple-comparisons test. (*$P < 0.05$, **$P < 0.01$, ***$P < 0.001$, ****$P < 0.0001$).

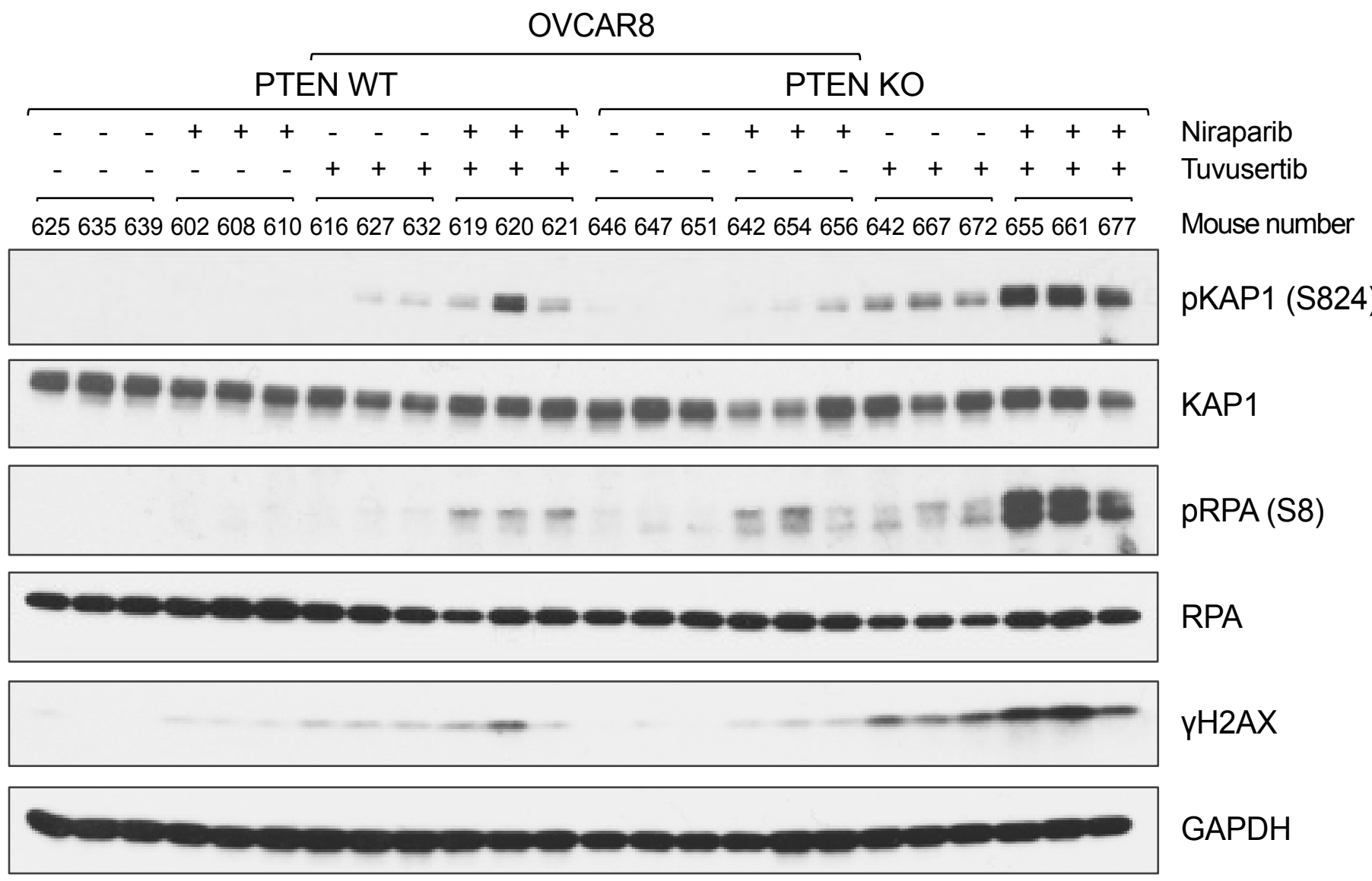

Supplemental Figure 5. ATR inhibitor monotherapy or combination treatment leads to increased replication stress and DNA damage in PTEN-deficient tumors. Western blot analysis of lysates from OVCAR8 and OVCAR8-PTEN-KO xenograft tumors after 7 days of treatment, showing increased replication stress and DNA damage with ATR inhibition alone and in combination with PARP inhibition in PTEN-deficient tumors.

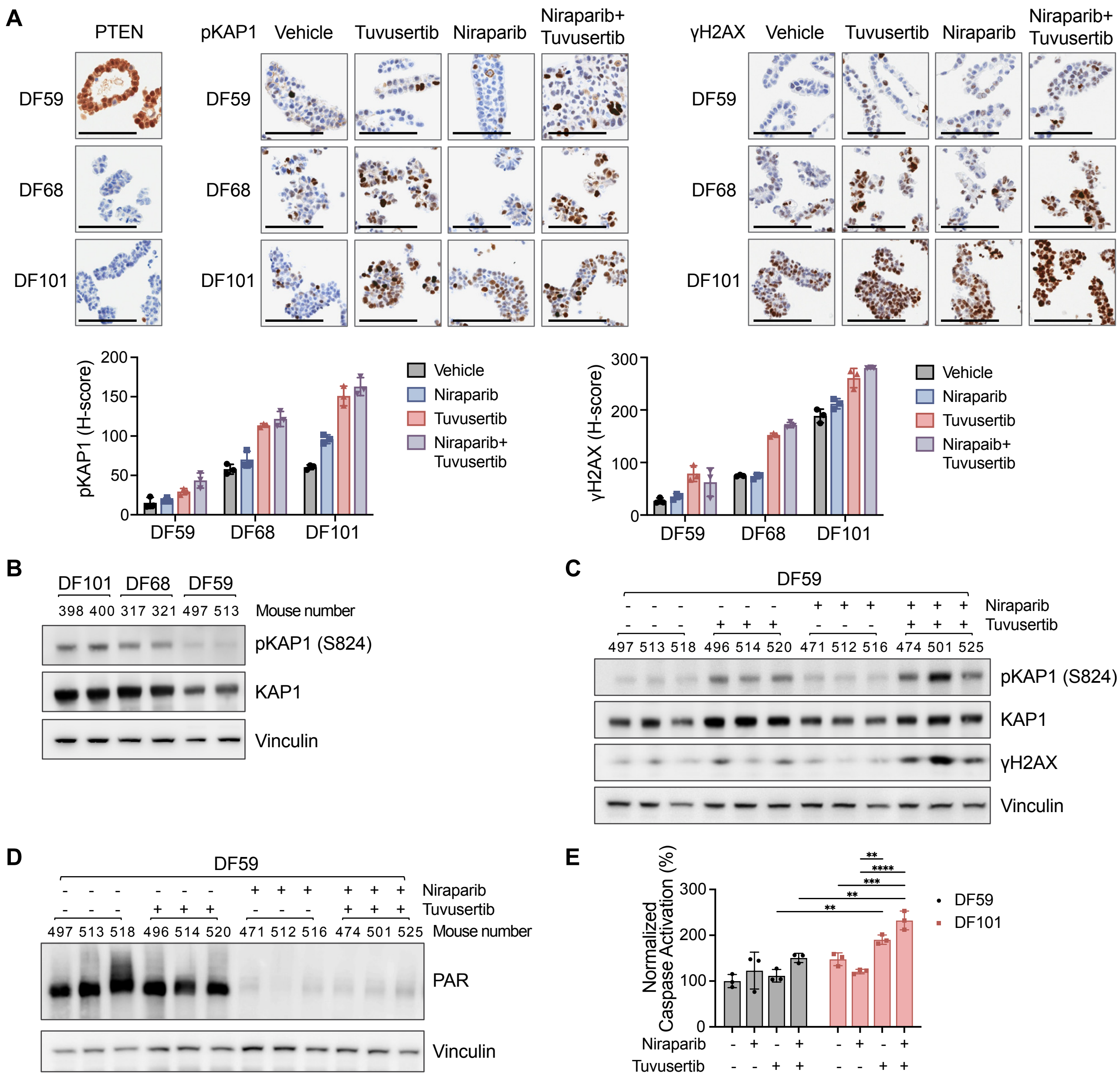

Supplemental Figure 6. Combined ATR and PARP inhibition in PTEN-deficient models induces extensive replication stress, DNA damage and apoptosis. (**A**) Immunohistochemical (IHC) staining of PDX tumors for pKAP1 and γH2AX. PTEN-deficient models displayed higher basal levels of replication stress (pKAP1) and DNA damage (γH2AX), which were further elevated upon ATR inhibition or combination therapy. Scale bars: 100 µm. Data represent mean ± SD from 3 samples per treatment group. (**B**) Western blot analysis of untreated tumor lysates from three PDX models, demonstrating higher basal levels of pKAP1 in PTEN-deficient models (DF101, DF68). (**C**) Western blot analysis of DF59 tumor lysates after treatment, indicating increased pKAP1 and γ-H2AX levels with combination therapy compared to monotherapies. (**D**) Western blot analysis of tumor lysates from the DF59 model post-treatment, showing a significant reduction in PAR levels following PARP inhibition. (**E**) Caspase-Glo® 3/7 assay measuring apoptosis in tumor samples from PTEN-proficient (DF59) and PTEN-deficient (DF101) models. Tuvusertib induced greater apoptosis in PTEN-deficient cells, further enhanced by PARP inhibition. Data represent mean ± SD. Statistical significance was determined using one-way ANOVA with Tukey's post hoc test (** $P < 0.01$, *** $P < 0.001$, **** $P < 0.0001$).

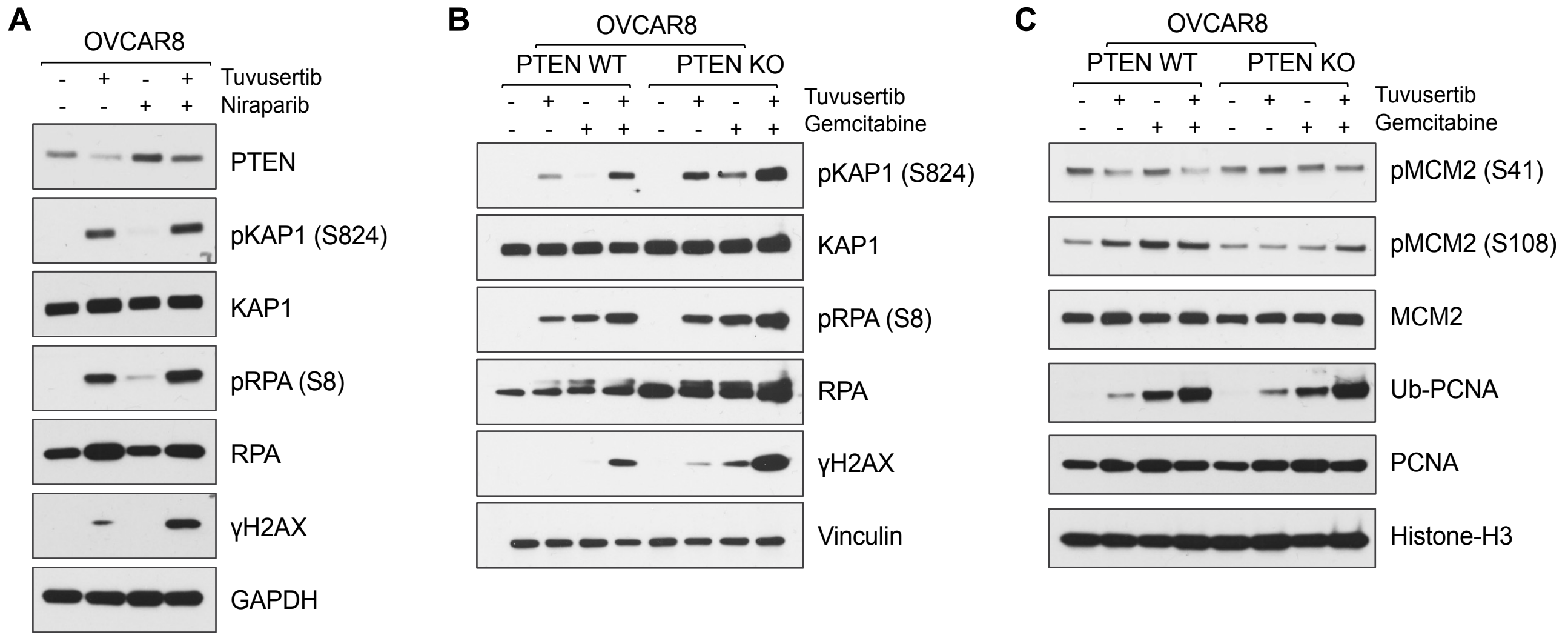

Supplemental Figure 7. PTEN deficiency confers increased sensitivity to ATR inhibition combined with gemcitabine. (**A**) Western blot analysis of lysates from OVCAR8 cells treated with tuvusertib and niraparib for 24 hours. ATR inhibition alone and in combination with niraparib induced increased replication stress and DNA damage in PTEN-deficient cells. Notably, niraparib and combination treatment upregulated PTEN expression. (**B**) Western blot analysis of lysates from OVCAR8 and OVCAR8 PTEN-KO cells treated with tuvusertib and gemcitabine for 24 hours, demonstrating increased replication stress and DNA damage in PTEN-deficient cells. (**C**) Western blot analysis of chromatin-bound fractions from WT and PTEN-KO cells treated with ATR inhibitor and gemcitabine, showing altered levels of phosphorylated MCM2 (pMCM2) and ubiquitinated PCNA (Ub-PCNA).

Supplemental Table 1

REAGENT	SOURCE	CATALOGUE NUMBER
Antibodies		
Phospho-ATM (Ser1981)	Cell Signaling Technology	4526
ATM	Cell Signaling Technology	2873
Phospho-ATR (Thr1989)	Cell Signaling Technology	30632
ATR	Cell Signaling Technology	13934
Phospho-Akt (Ser473)	Cell Signaling Technology	9271
BrdU	Santa Cruz Biotechnology	sc-32323
BrdU	Abcam	ab6326
BrdU	BD Biosciences	347580
APC anti-BrdU	BioLegend	364114
Phospho-Chk1 (Ser345)	Cell Signaling Technology	2348
CHK1	Cell Signaling Technology	2360
CHK1	Santa Cruz Biotechnology	sc-7898
Cleaved PARP (Asp214)	Cell Signaling Technology	5625
Cleaved Caspase-3 (Asp175)	Cell Signaling Technology	9664
Phospho-DNA-PKcs (Ser2056)	Abcam	ab18192
DNA-PKcs	Cell Signaling Technology	4602
GAPDH	Cell Signaling Technology	2118
Phospho-Histone H2A.X (Ser139)	Cell Signaling Technology	2577
Phospho-Histone H2A.X (Ser139)	EMD Millipore Sigma	05-636
Phospho-Histone H2A.X (Ser139)	Abcam	ab26350
Histone H3	Cell Signaling Technology	4499
Alexa Fluor® 488 anti-Histone H3 Phospho (Ser10)	BioLegend	650804
Phospho-KAP-1 (Ser824)	Cell Signaling Technology	4127
Phospho-KAP-1 (Ser824)	Abcam	ab70369

Phospho-KAP-1 (Ser824)	Abcam	ab133440
KAP-1	Cell Signaling Technology	4123
KAP-1	Abcam	ab10484
Phospho-MCM2 (S41)	BETHYL	A300-117A
Phospho-MCM2 (S108)	BETHYL	A300-094A
MCM2	Cell Signaling Technology	4007
Ubiquityl-PCNA (Lys164)	Cell Signaling Technology	13439
PAR/pADPr	Bio-Techne Corporation	4335-MC-100
PCNA	Cell Signaling Technology	2586
PTEN	Cell Signaling Technology	9552
Rad51	Cell Signaling Technology	8875
Phospho-RPA32/RPA2 (Ser8)	Cell Signaling Technology	54762
Phospho-RPA32/RPA2 (Ser4, Ser8)	BETHYL	A300-245A
RPA32/RPA2	Cell Signaling Technology	52448
RPA32	BETHYL	A300-244A
ssDNA	DSHB	AB_10805144
Vinculin	Santa Cruz Biotechnology	sc-25336
Vinculin	Abcam	ab91459
Amersham ECL Mouse IgG, HRP-linked whole Ab	Cytiva Life Sciences	NA931-1ML
Amersham ECL Rabbit IgG, HRP-linked whole Ab	Cytiva Life Sciences	NA934-1ML
Fluorescein (FITC) AffiniPure™ Donkey Anti-Rabbit IgG (H+L)	Jackson ImmunoResearch Laboratories	711-095-152
Texas Red AffiniPure™ Donkey Anti-Mouse IgM mu chain	Jackson ImmunoResearch Laboratories	715-075-020
Brilliant Violet 480™ AffiniPure™ Goat Anti-Mouse IgG (H+L)	Jackson ImmunoResearch Laboratories	115-685-166
Goat Anti-Rat IgG H&L (Cy5 ®) preadsorbed	Abcam	ab6565
Goat Anti-Mouse IgG H&L (Cy3 ®) preadsorbed	Abcam	ab97035
Experimental models: Cell lines		
JHOS2	Parmar et al. (14)	N/A

OVCAR3	Parmar et al. (14)	N/A
KURAMOCHI	Parmar et al. (14)	N/A
TYKNU	Laboratory of Dipanjan Chowdhury	N/A
COV362	Parmar et al. (14)	N/A
ES2	Parmar et al. (14)	N/A
OVCAR8	Laboratory of Dipanjan Chowdhury	N/A
TOV21G	Parmar et al. (14)	N/A
PEO1	Parmar et al. (14)	N/A
PEO4	Parmar et al. (14)	N/A
OV90	Parmar et al. (14)	N/A
OVSAHO	Parmar et al. (14)	N/A
Experimental models: Organisms/strains		
Patient-derived xenografts (PDX)	Liu et al. (32)	N/A
NOD.Cg-*Prkdc*^{scid} *Il2rg*^{tm1Wjl}/SzJ (NSG mice)	The Jackson Laboratory	005557
NU/J (Nude mice)	The Jackson Laboratory	002019
Chemicals, peptides, and recombinant proteins		
Tuvusertib (M1774)	EMD Serono	N/A
Elimusertib (BAY-1895344)	Selleck Chemicals	S8666
Niraparib (MK-4827)	MedChemExpress	HY-10619
Gemcitabine	Selleck Chemicals	S1714
5-bromo-2’-deoxyuridine (BrdU)	Sigma-Aldrich	B5002
4′,6-diamidino-2-phenylindole (DAPI)	Invitrogen	D3571
DNaseI	Sigma Aldrich	D4513-1VL
5-Iodo-2’-deoxyuridine (IdU)	Sigma Aldrich	I7125
5-Chloro-2’-deoxyuridine (CldU)	Sigma Aldrich	C6891
Recombinant DNA		
psPAX2	Addgene	12260

VSV.G	Addgene	14888
WT PTEN_pGenlenti	GenScript	SC1010
C124S_pGenlenti	GenScript	SC1441
G129E_pGenlenti	GenScript	SC1441
Critical Commercial Assays		
CellTiter-Glo® Luminescent Cell Viability Assay	Promega	G7573
Caspase-Glo® 3/7 Assay System	Promega	G8090
FiberPrep DNA extraction kit	Genomic Vision	IFU-M&S13-c
Oligonucleotides: sgRNA sequences		
sgPTEN-1	Life Technologies	CRISPR766883_SGM
sgPTEN-2	Life Technologies	CRISPR766902_SGM
