# Supplementary material for: PTEN deficiency confers sensitivity to ATR inhibitor–based treatment in high-grade serous ovarian cancer
Source: J Clin Invest. 2026 Jul 7;136(16):e197645. doi: 10.1172/JCI197645 (PMC13476146; doi:10.1172/JCI197645)

Full unedited blot/gel for Figure 1

Figure 1B

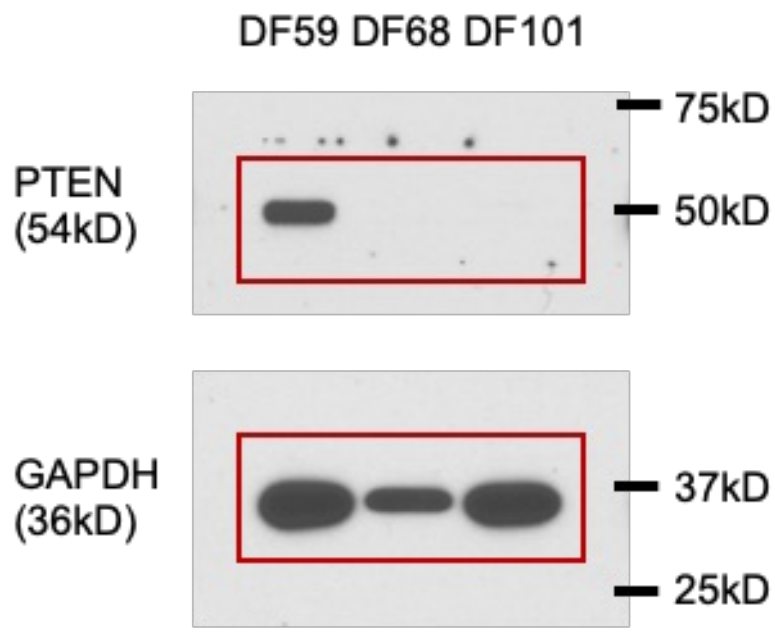

Figure 1C

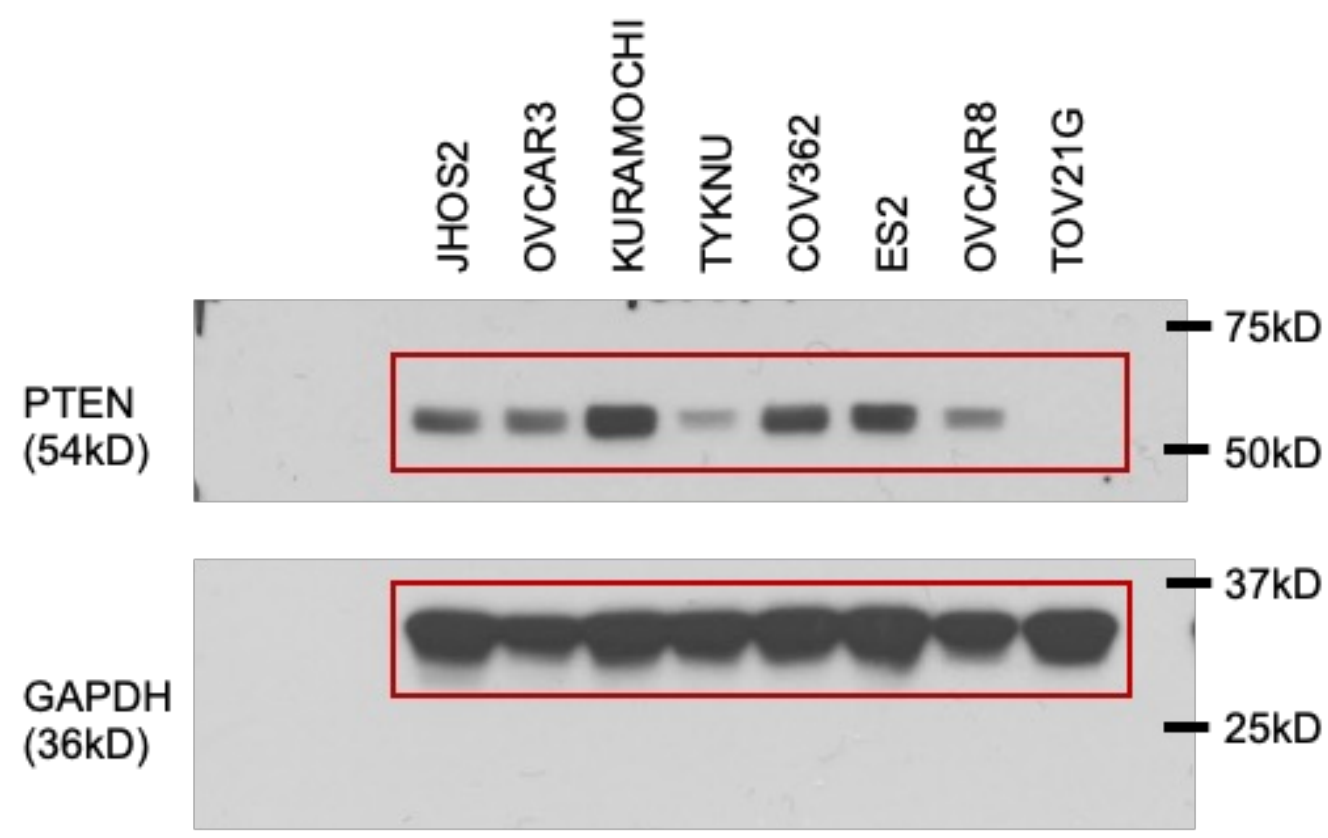

Figure 1E

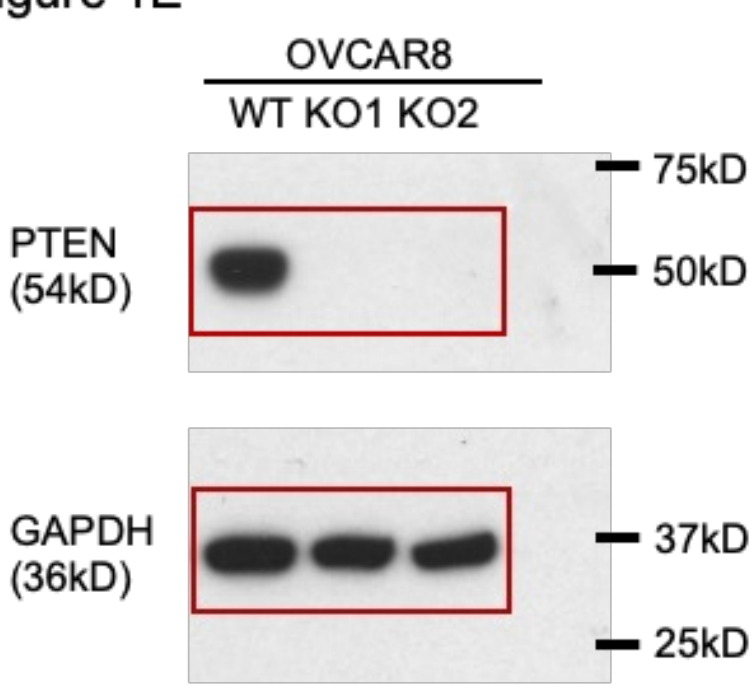

Figure 1G

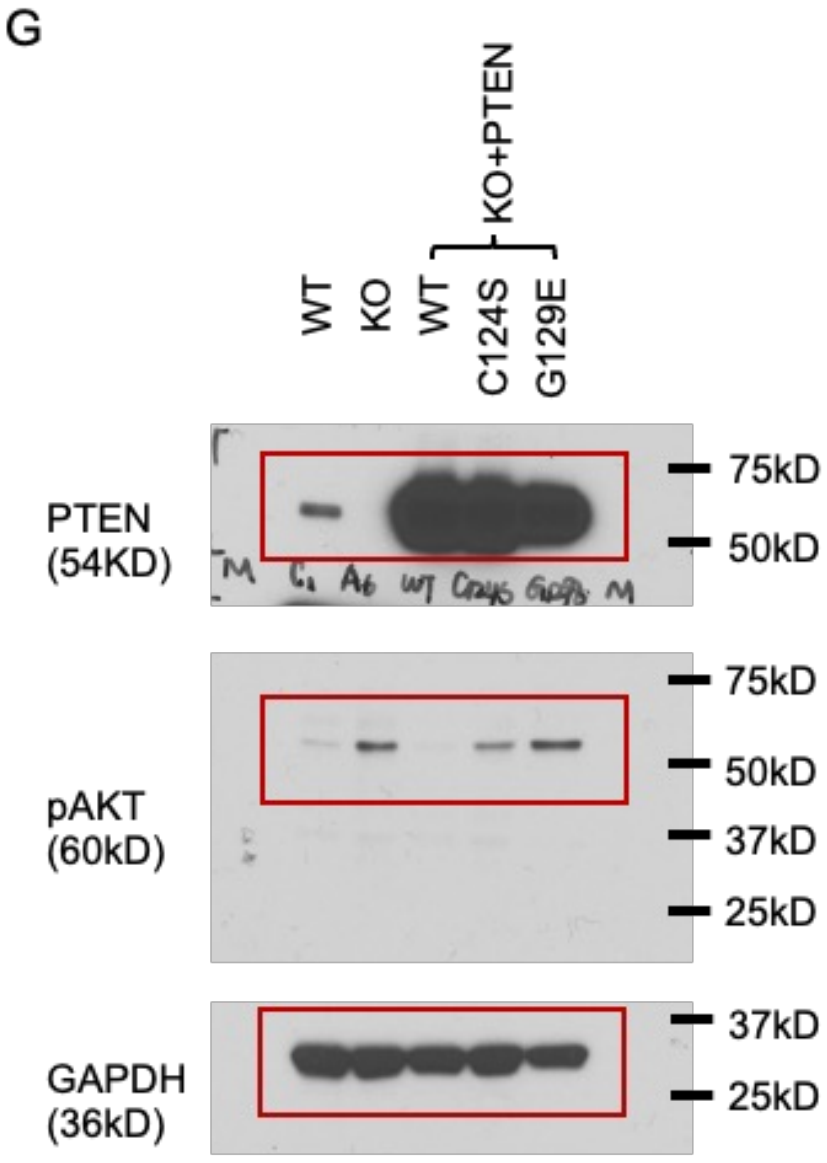

Full unedited blot/gel for Figure 2

Figure 2A

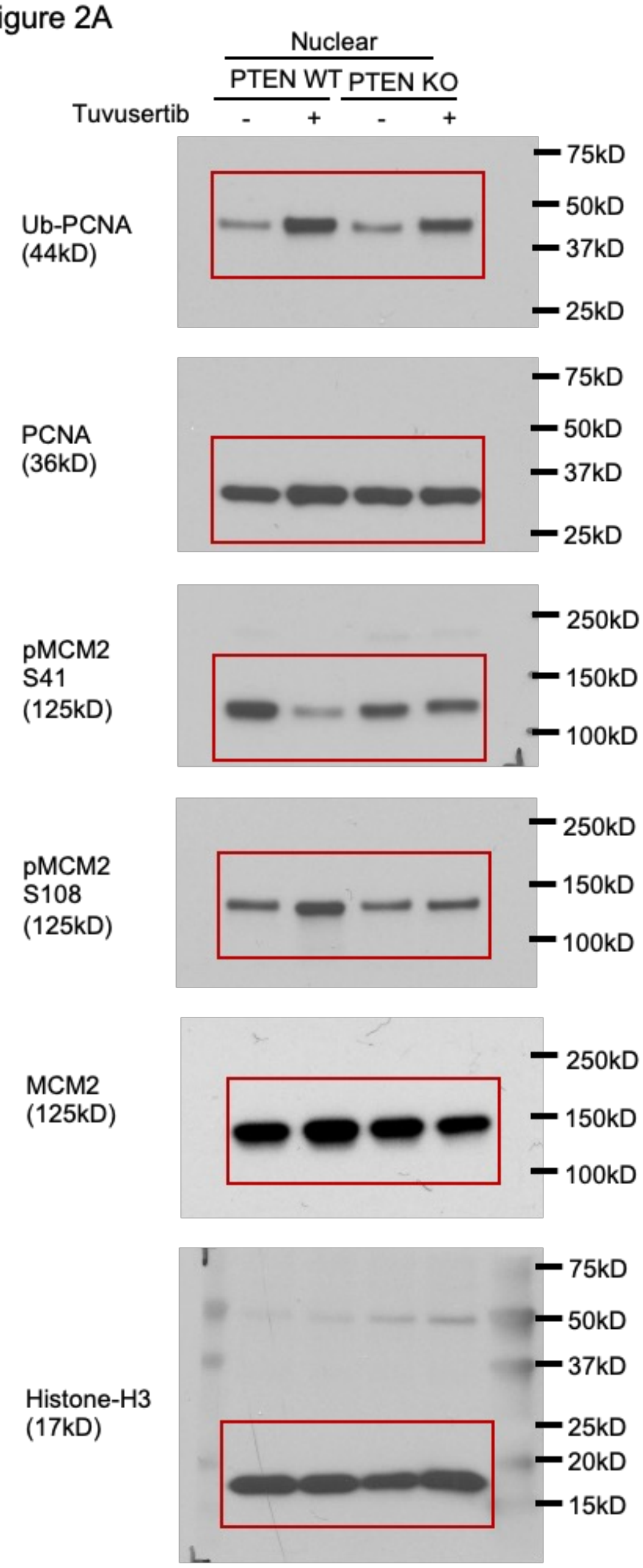

Figure 2G

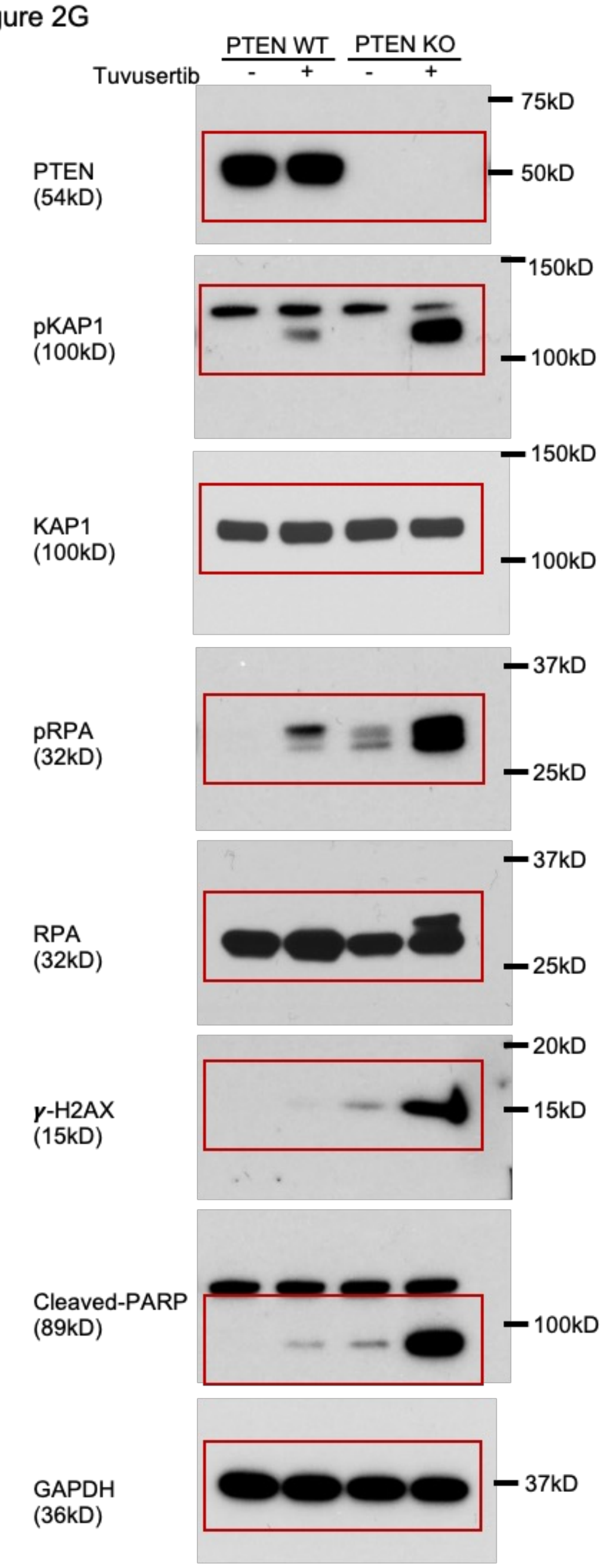

Full unedited blot/gel for Figure 3

Figure 3A

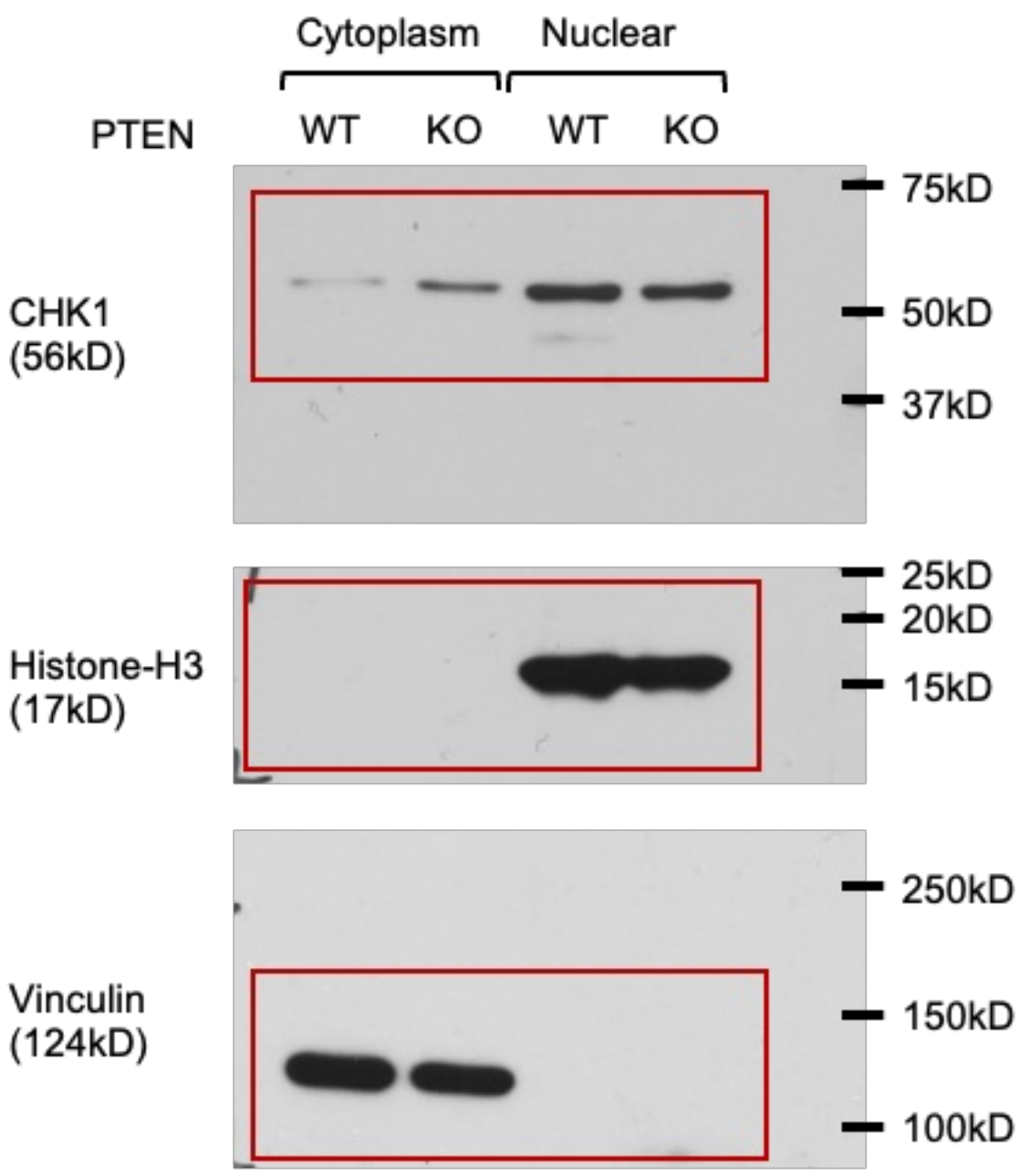

Figure 3G

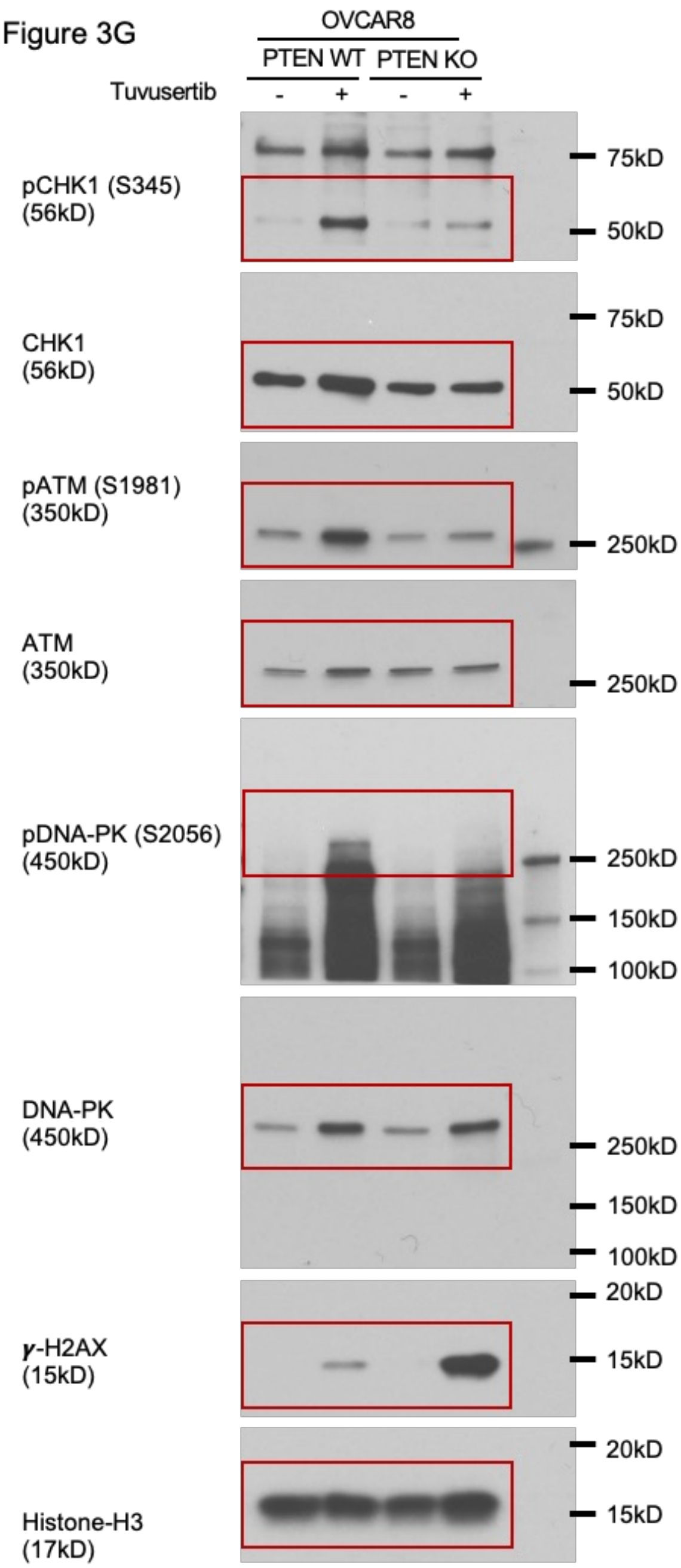

Full unedited blot/gel for Figure 3

Figure 3H

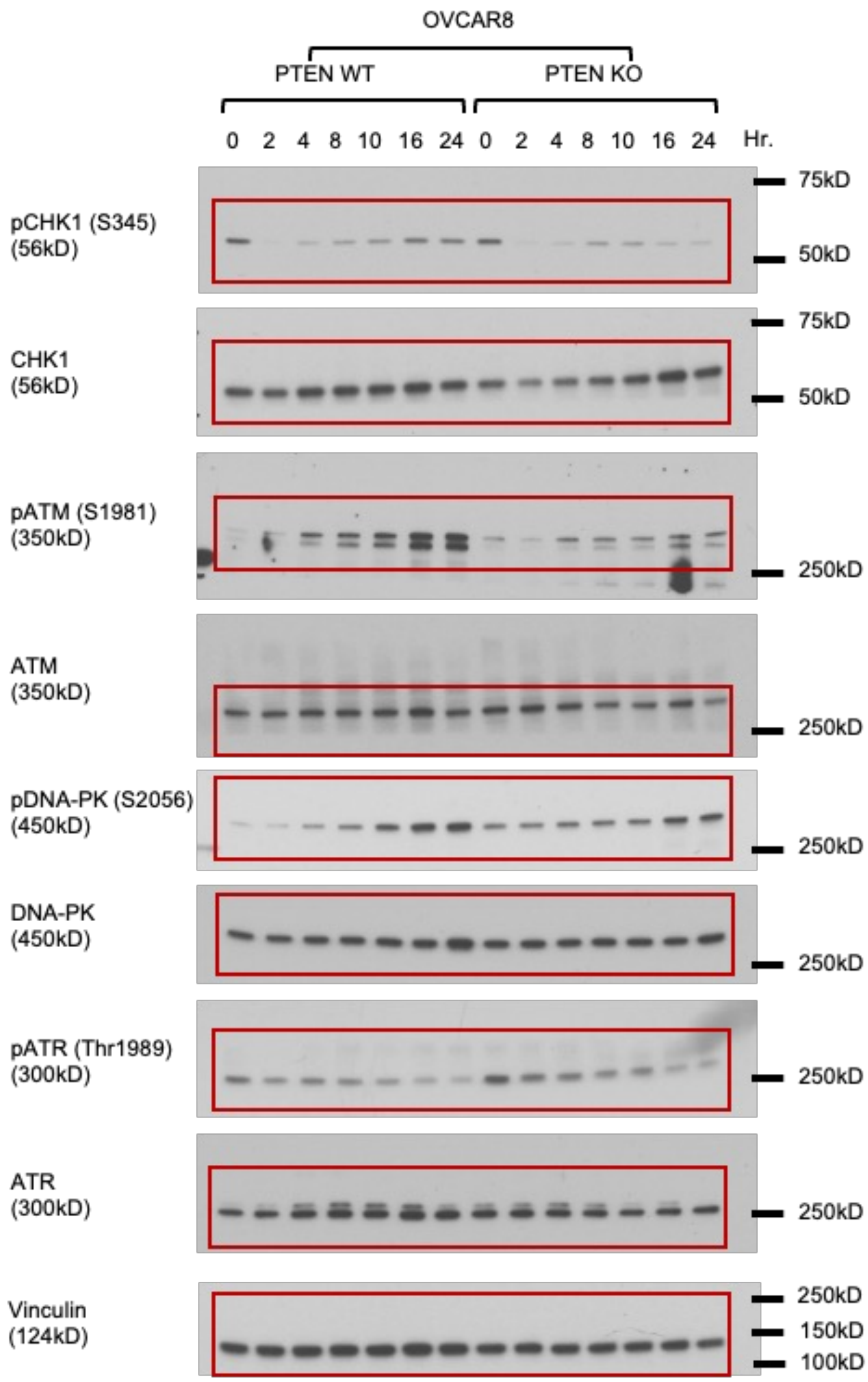

Full unedited blot/gel for Figure 4

Figure 4C

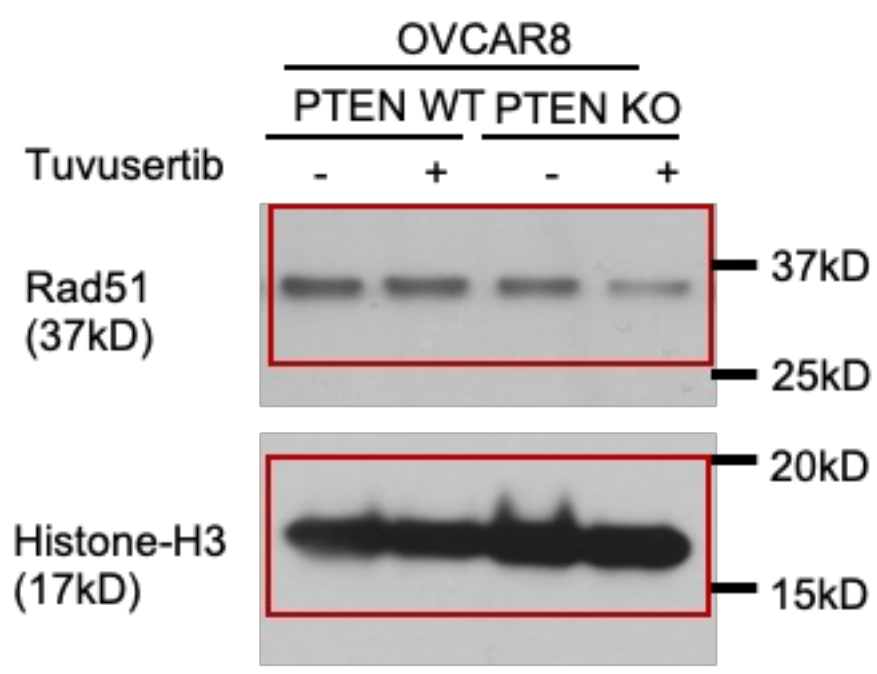

Full unedited blot/gel for Supplemental Figure 1

Supplemental Figure 1A

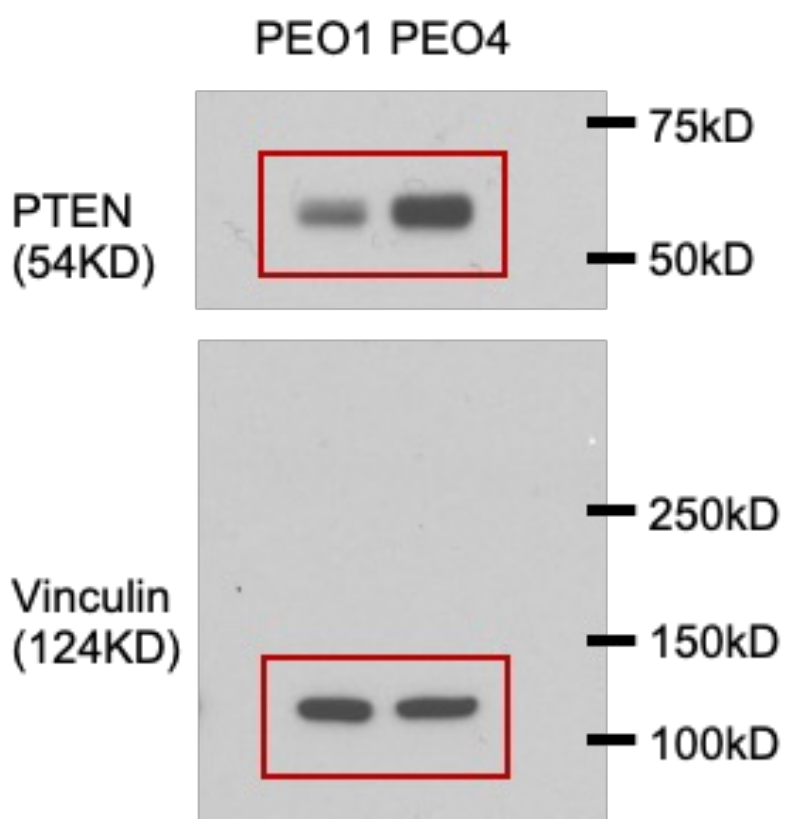

Supplemental Figure 1F

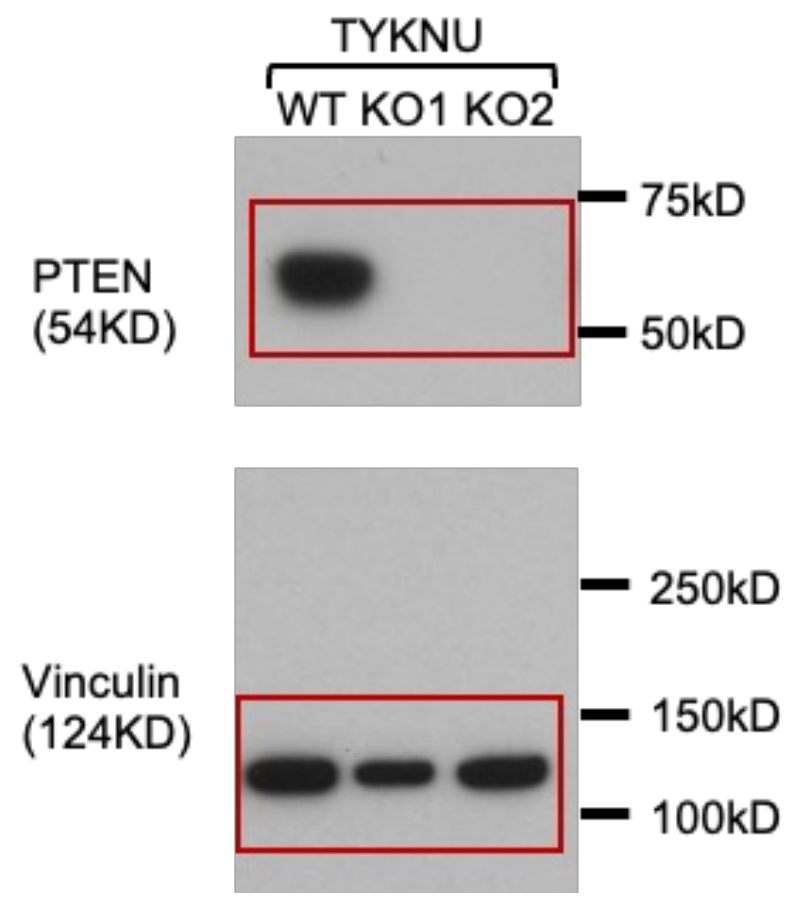

Supplemental Figure 1G

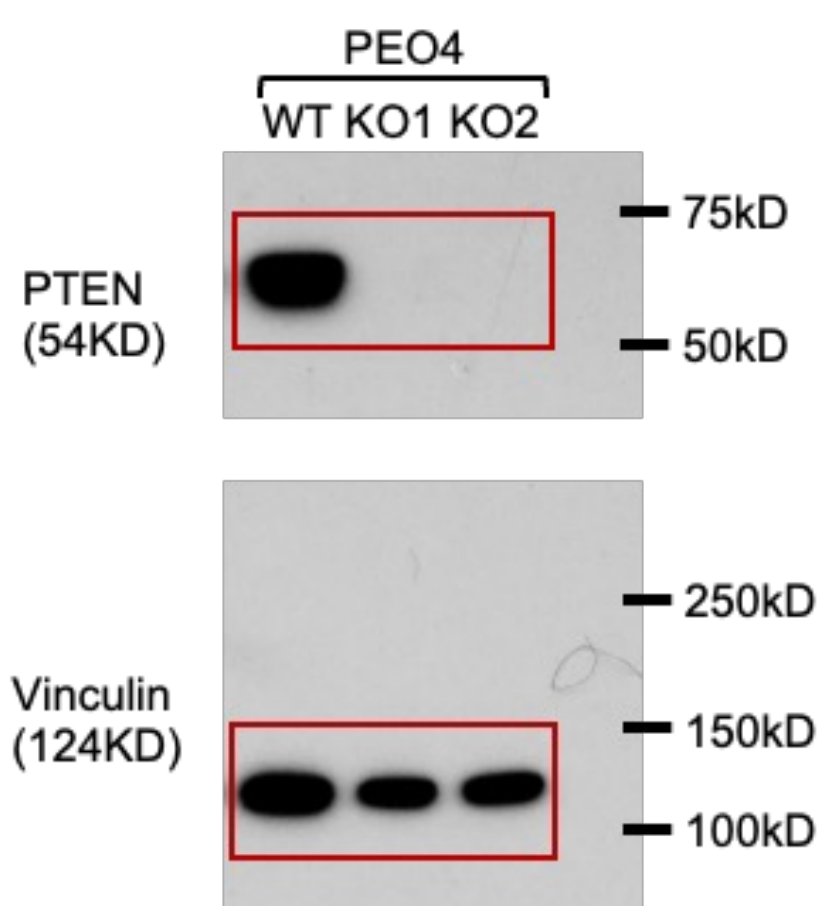

Full unedited blot/gel for Supplemental Figure 2

Supplemental Figure 2A

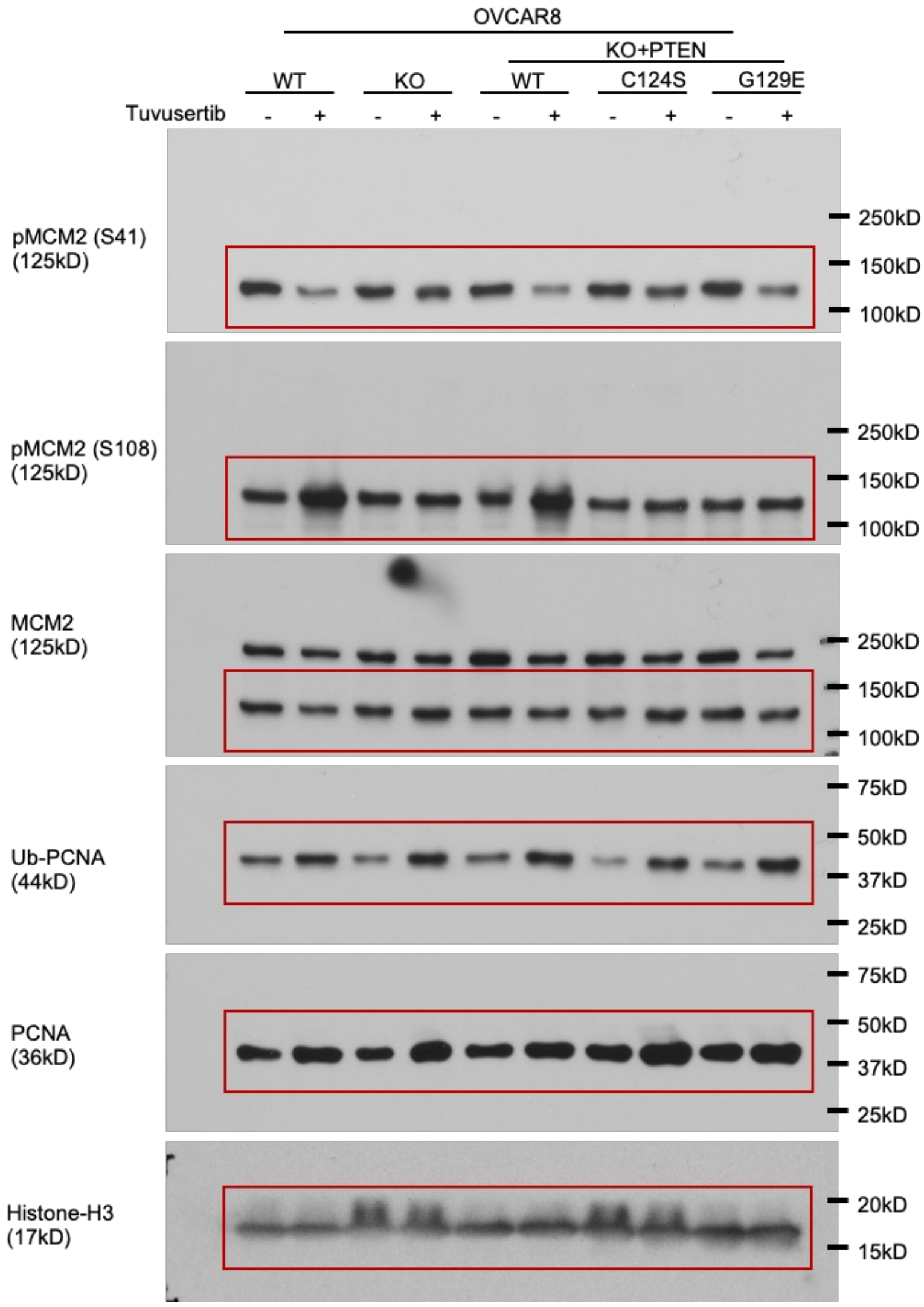

Supplemental Figure 2C

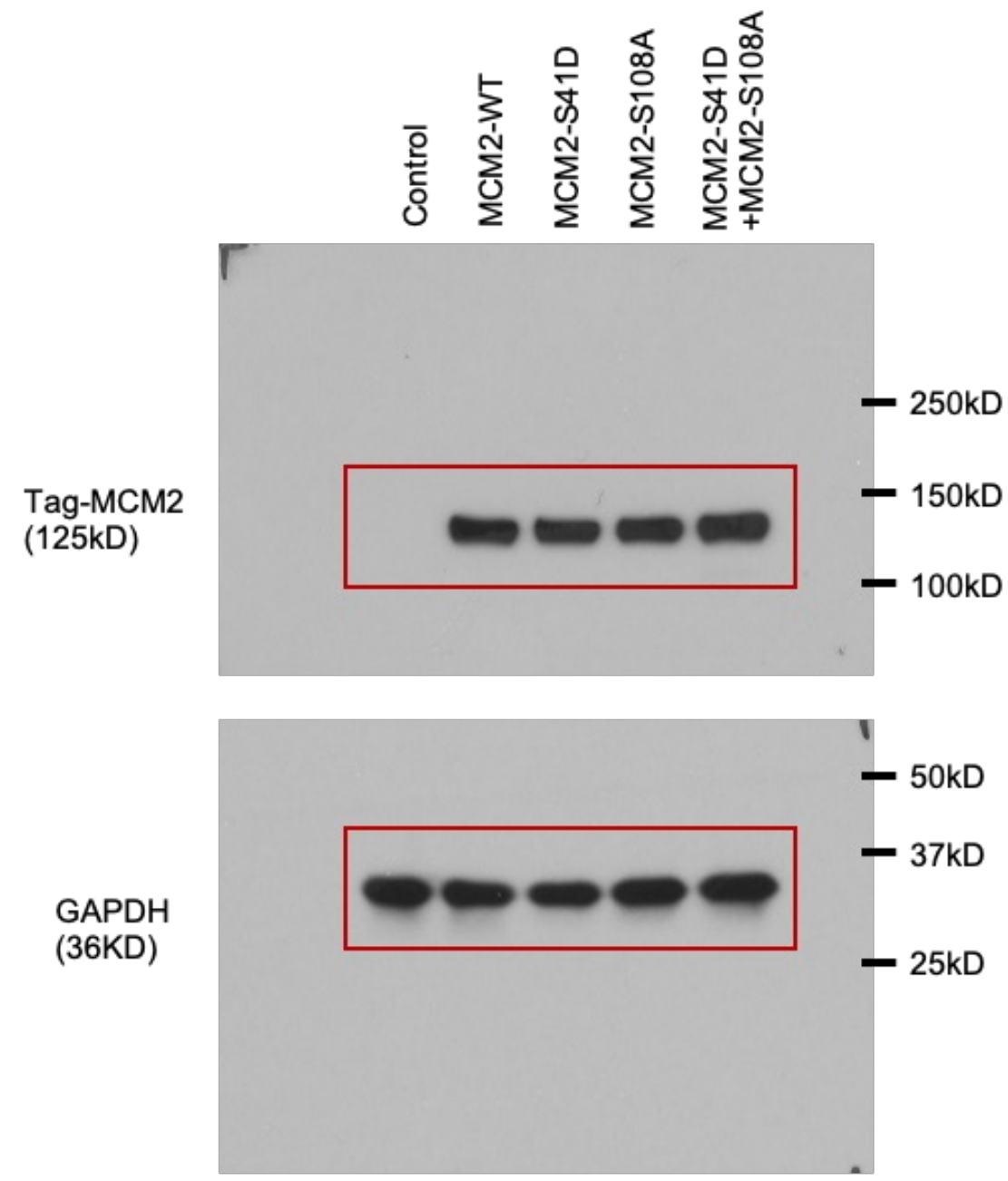

Full unedited blot/gel for Supplemental Figure 2

Supplemental Figure 2E

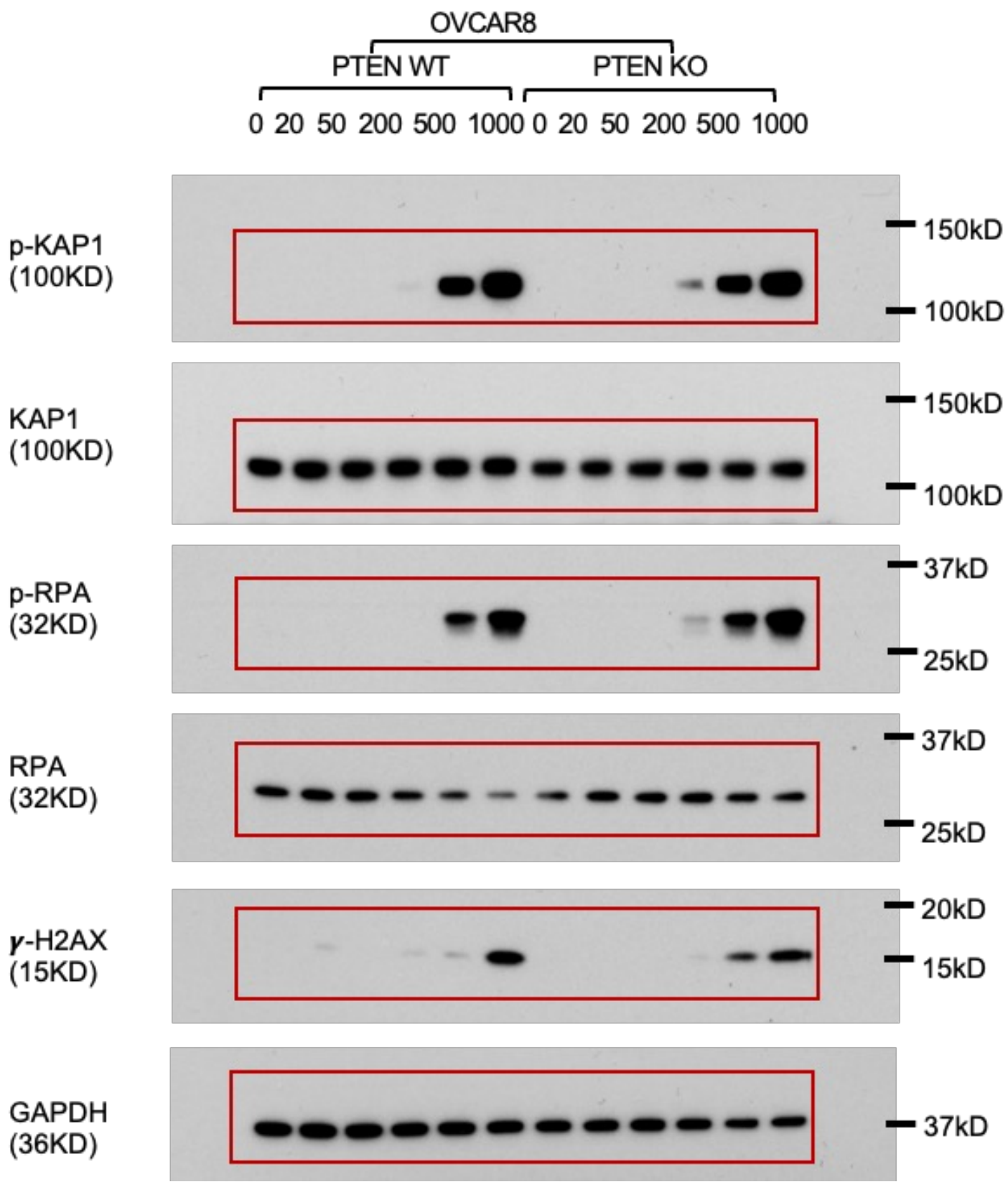

Supplemental Figure 2F

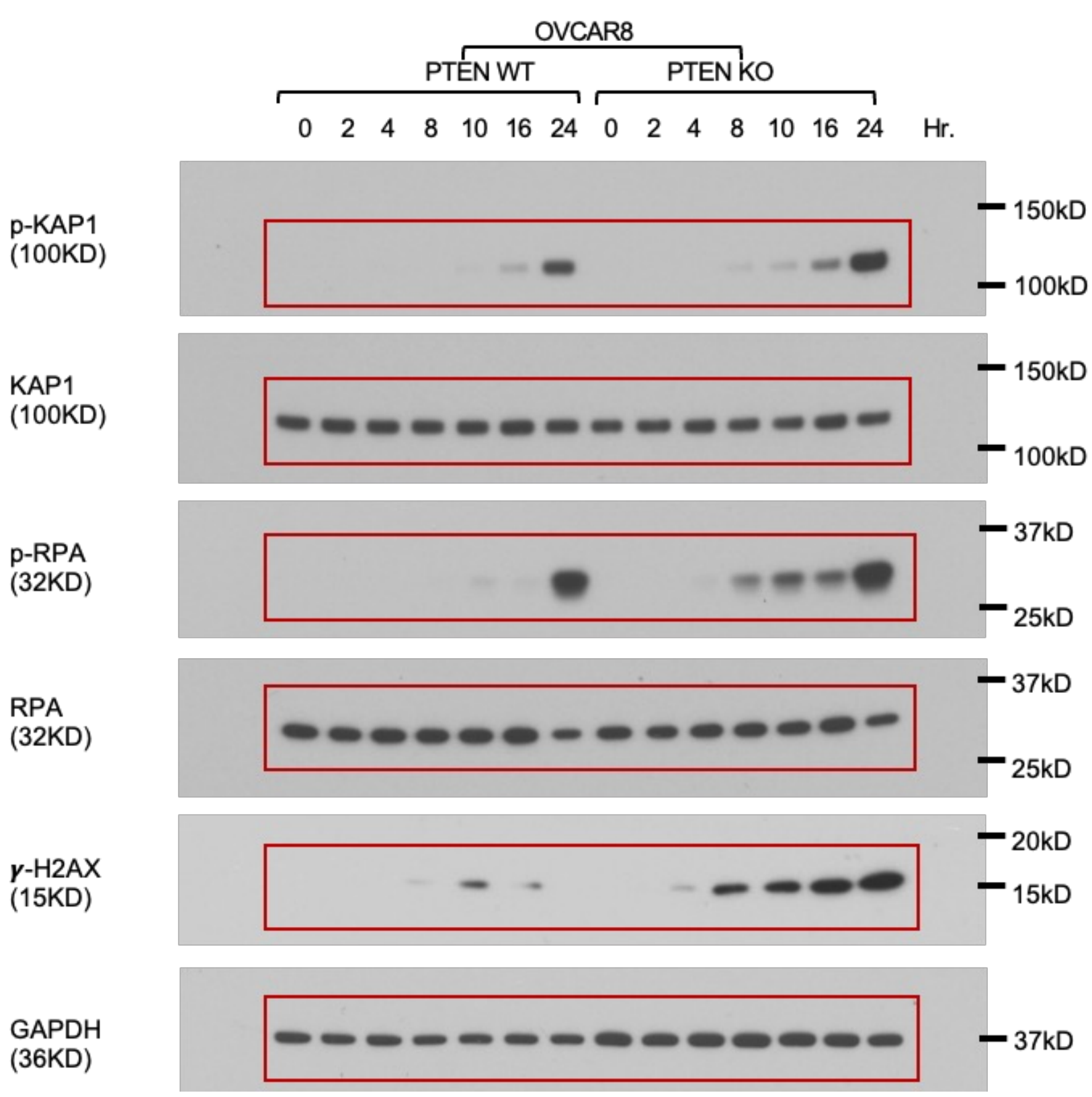

Full unedited blot/gel for Supplemental Figure 2

Supplemental Figure 2G

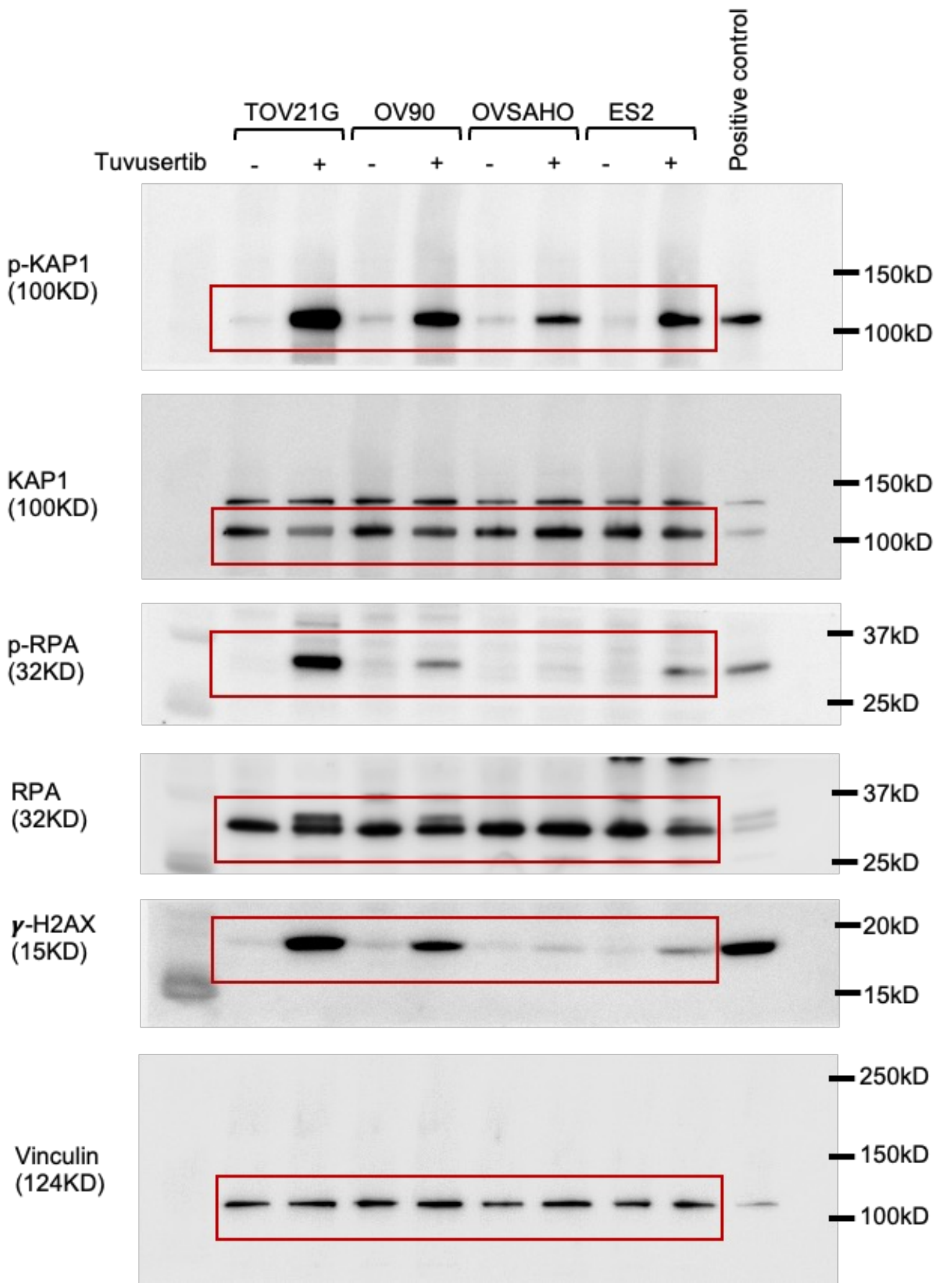

Full unedited blot/gel for Supplemental Figure 4

Supplemental Figure 4A

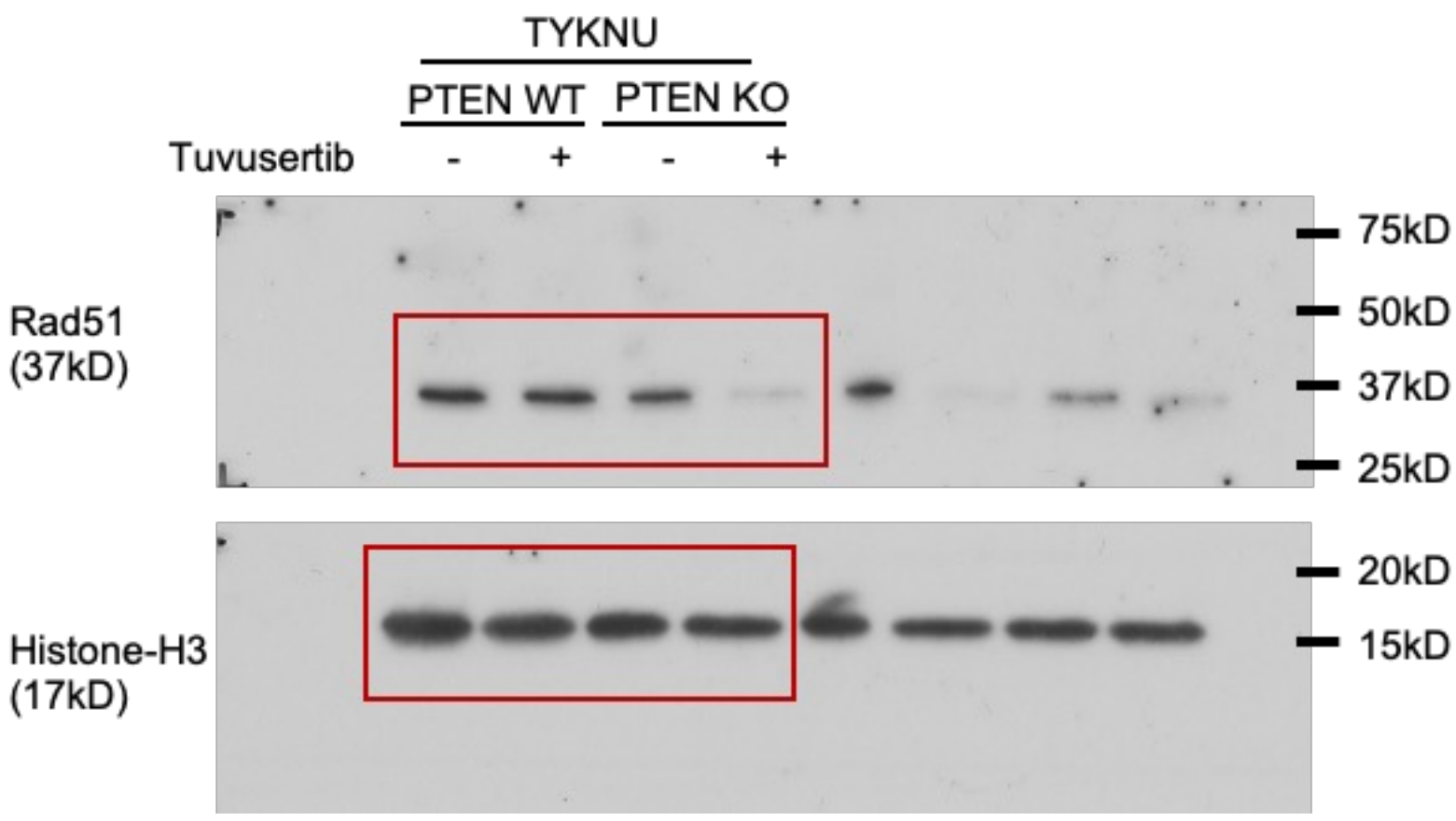

Full unedited blot/gel for Supplemental Figure 5

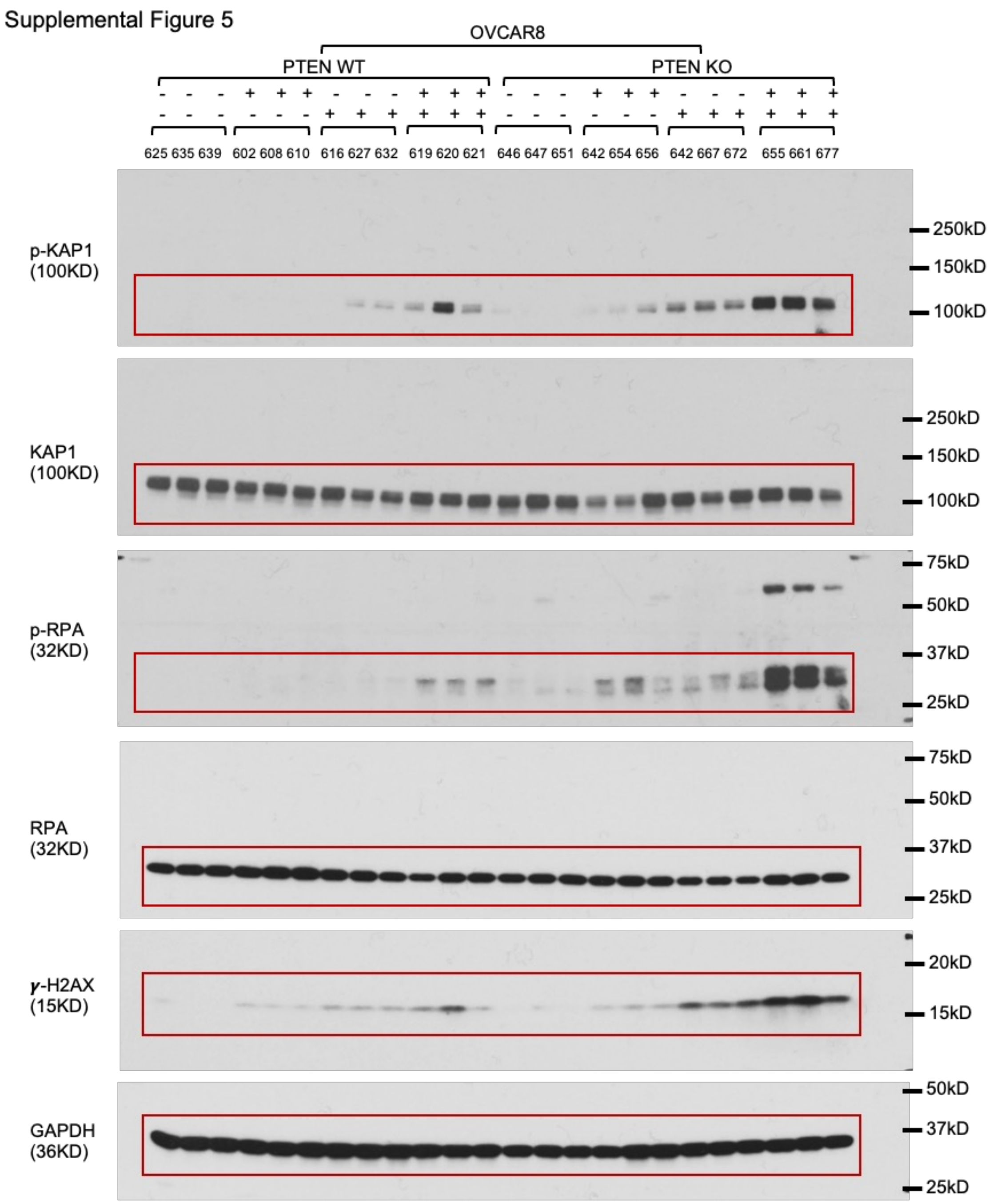

Full unedited blot/gel for Supplemental Figure 6

Supplemental Figure 6B

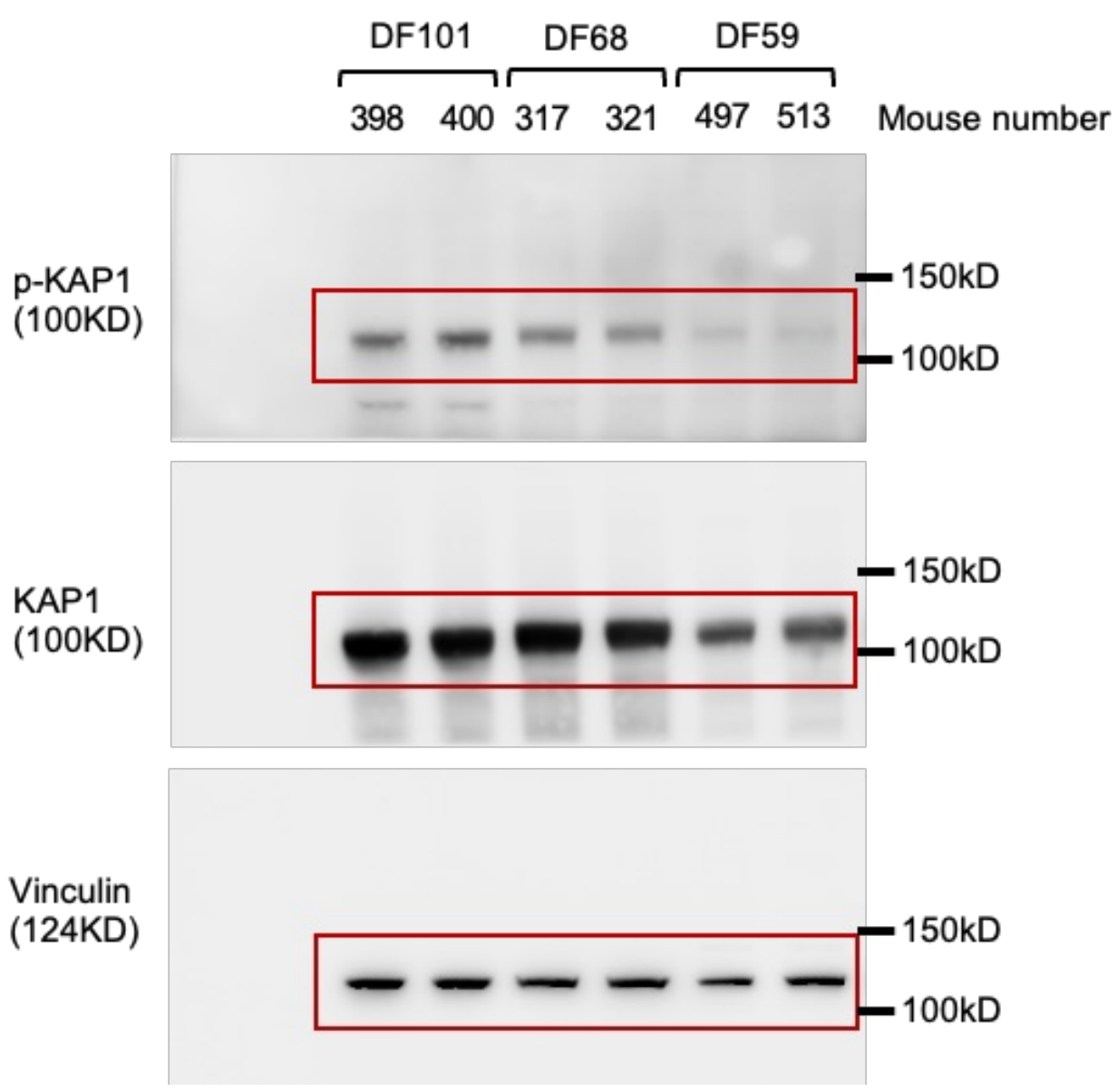

Supplemental Figure 6C

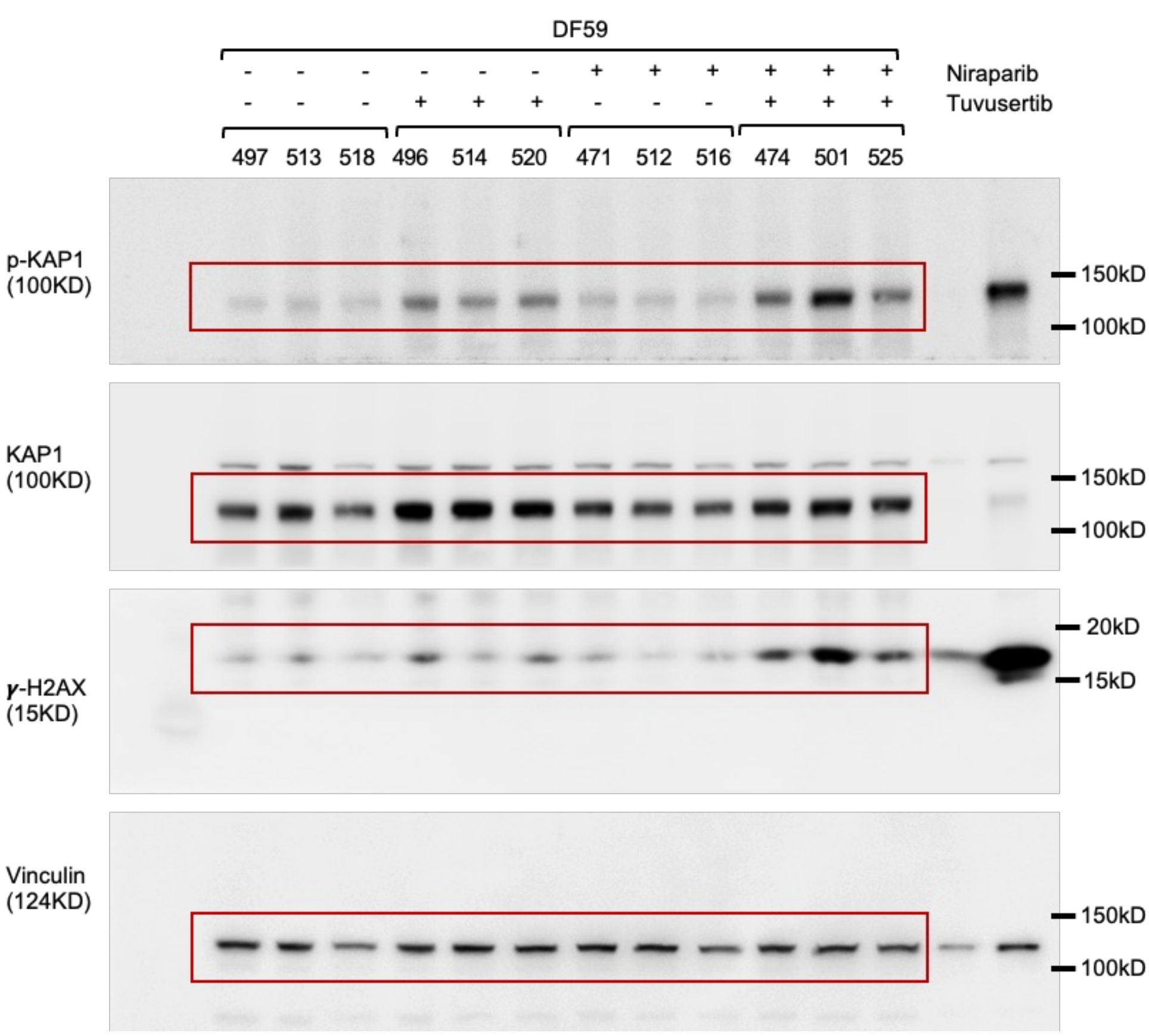

Full unedited blot/gel for Supplemental Figure 6

Supplemental Figure 6D

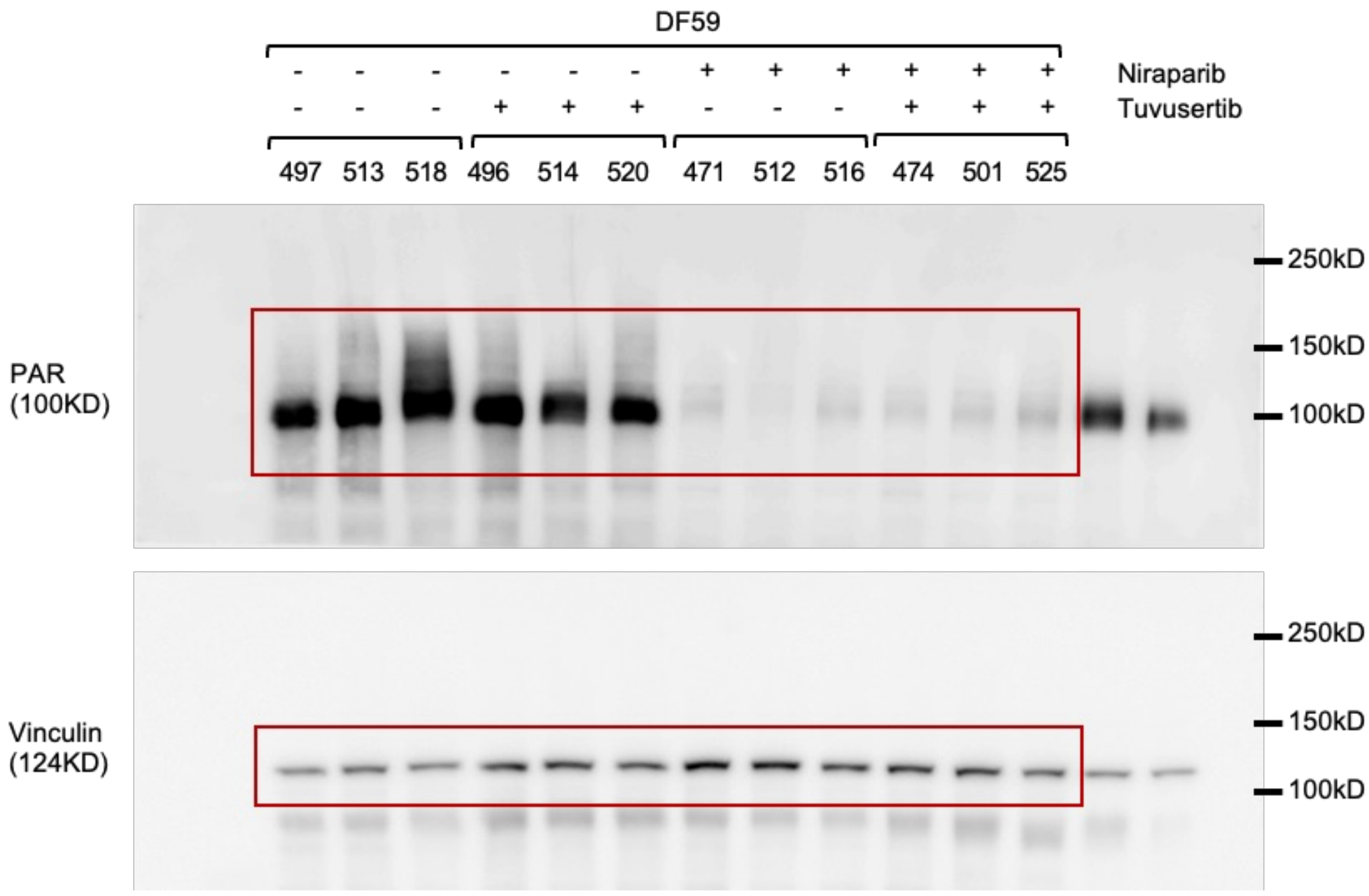

Full unedited blot/gel for Supplemental Figure 7

Supplemental Figure 7A

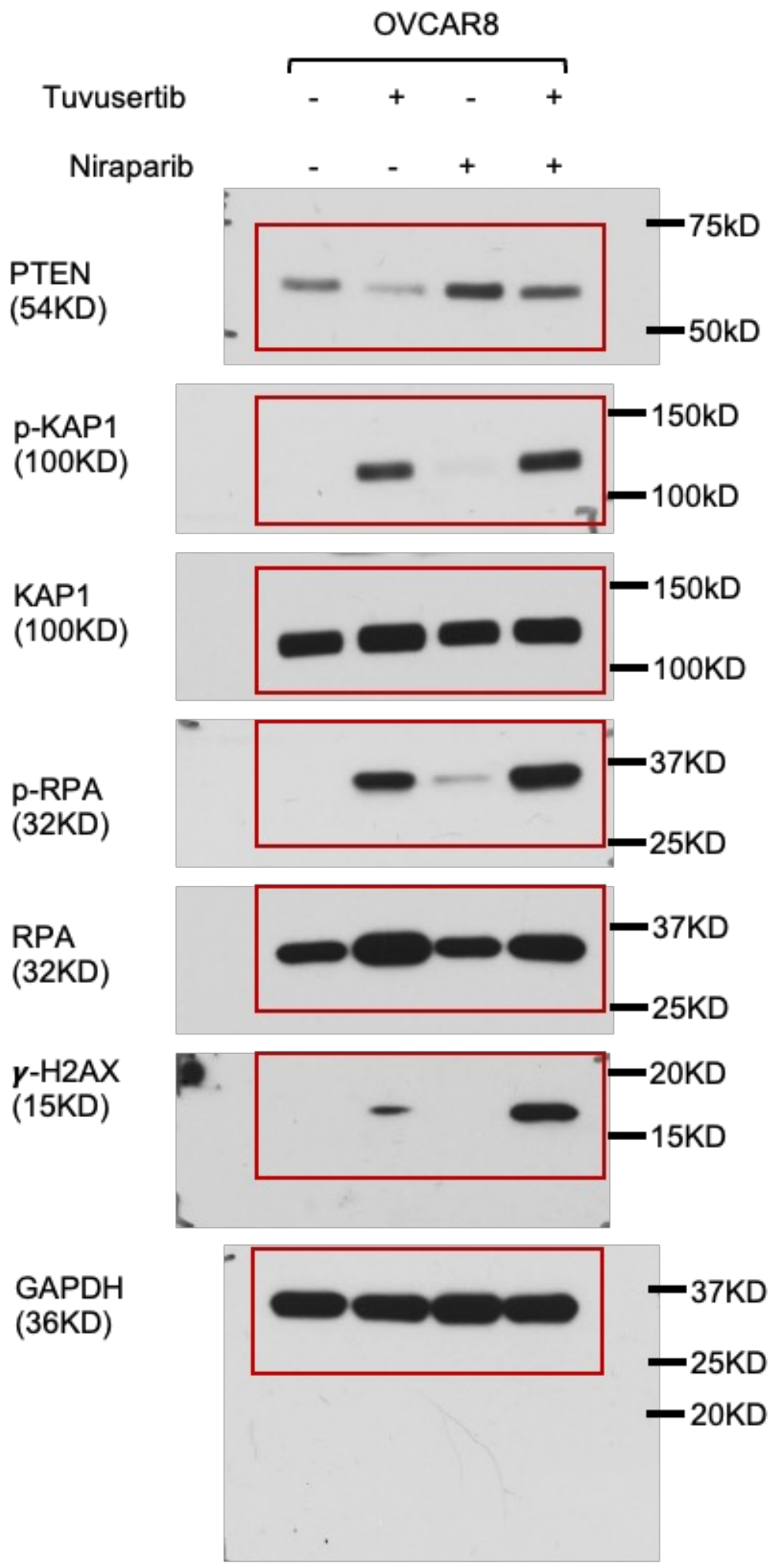

Supplemental Figure 7B

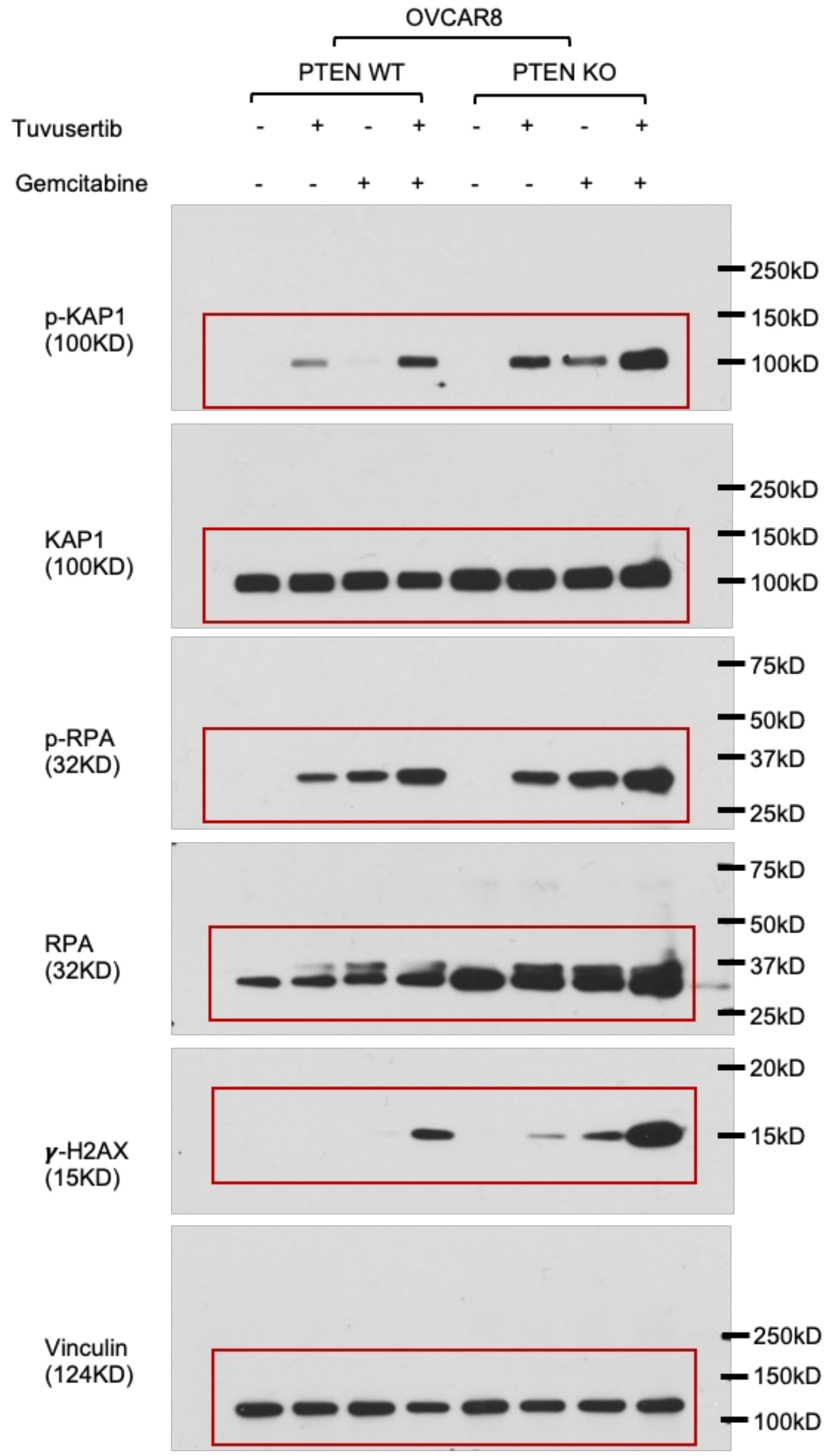

Full unedited blot/gel for Supplemental Figure 7

Supplemental Figure 7C

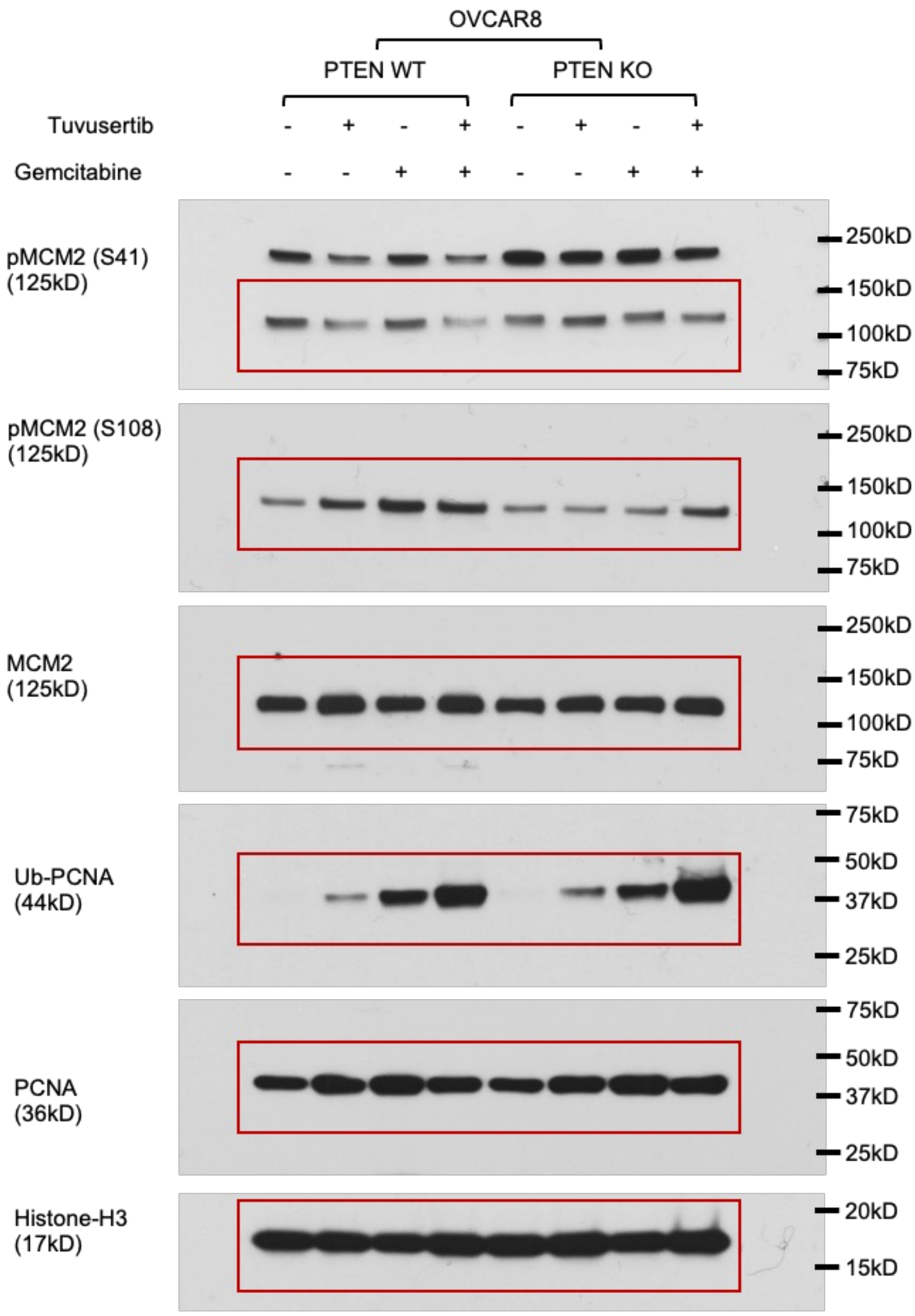

Supplement: Supplementary file 2 [file jci-136-197645-s009.pdf]
